# Supplementary material for: Genome-Wide Insight into Profound Effect of Carbon Catabolite Repressor (Cre1) on the Insect-Pathogenic Lifecycle of Beauveria bassiana
Source: J Fungi (Basel). 2021 Oct 23;7(11):895. doi: 10.3390/jof7110895 (PMC8622151; doi:10.3390/jof7110895)
Supplement: Supplementary file 1 [file jof-07-00895-s001.zip › jof-1425492-supplementary.pdf]

Supplementary Material

Genome-Wide Insight into Profound Effect of Carbon Catabolite Repressor (Cre1) on the Insect-Pathogenic Lifecycle of *Beauveria bassiana*

Rehab Abdelmonem Mohamed, Kang Ren, Ya-Ni Mou, Sheng-Hua Ying, Ming-Guang Feng*

MOE Laboratory of Biosystems Homeostasis & Protection, College of Life Sciences, Zhejiang University, Hangzhou, Zhejiang, 310058, China

* Correspondence to Ming-Guang Feng, mgfeng@zju.edu.cn

Contents:

Figure S1. Generation and identification of *cre1* mutants in *B. bassiana*, page 2

Table S1. Paired primers used for targeted gene manipulation of *cre1* in *B. bassiana*, page 2

Table S2. Paired primers used for transcriptional profiling of genes for insight into altered phenotypes of Δ*cre1* and/or validity of its transcriptome in *B. bassiana*, page 3

Table S3. A list of 1881 genes dysregulated in the Δ*cre1* mutant of *B. bassiana*, pages 4 to 34

Table S4. Counts of Δ*cre1*-specific dysregulated genes enriched to GO terms in three function classes of *B. bassiana* at the significant level of $p < 0.05$, pages 35 to 36

Table S5. Counts of Δ*cre1*-specific dysregulated genes enriched to the KEGG pathways of *B. bassiana* at the significant level of $p < 0.05$, page 37

Table S6. Lists of Δ*cre1*-specific dysregulated genes associated with phenotypic changes and involved in crucial cellular processes and events of *B. bassiana*, pages 38 to 50

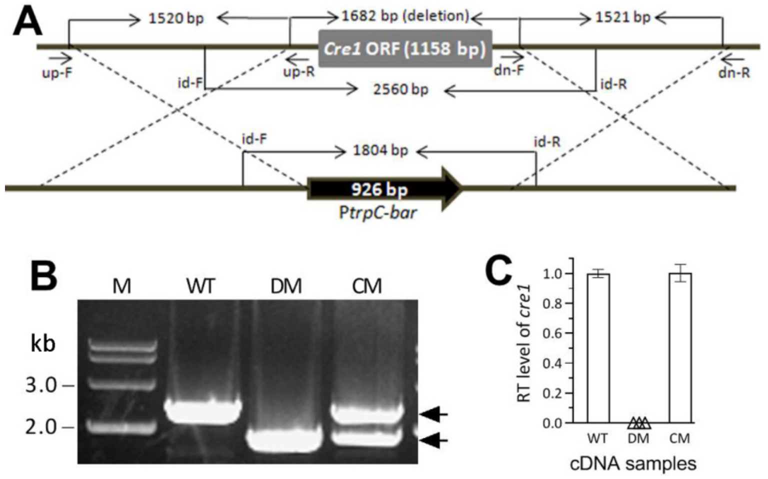

Figure S1. Generation and identification of *cre1* mutants in *B. bassiana*. (**A**) Schematic diagram for the strategy of *cre1* deletion. Note that a 1682-bp fragment containing the full-length coding nucleotide sequence (ORF) of cre1 and its flanking regions (524 bp) was expected to be deleted from the fungal wild-type strain (WT). (**B, C**) The *cre1* mutants (DM: deletion mutant Δ*cre1*; CM: complementation mutant Δ*cre1::cre1*) identified through PCR and qPCR analyses with paired primers (Table S1) respectively. M, DNA ladder. The arrows indicate PCR-detected fragments of 2560 and 1804 bp for WT and DM, respectively and both of them for CM. The PCR-detected fragments demonstrate that *cre1* was deleted as expected (2560 + 926 − 1804 = 1682 bp). The qPCR analysis of cDNA samples derived from 3-day-old SDAY cultures confirms that transcriptional expression of cre1 is completely abolished in DM and restored to the WT level in CM generated through targeted gene complementation. Error bars: standard deviations of the means from three independent cDNA samples analyzed via qPCR.

Table S1. Paired primers used for targeted gene manipulation of *cre1* in *B. bassiana*.

Primers	Paired sequences (5′–3′) [a]	Purpose [b]
CreA-G-F/R	CATGGACGAGCTGTACAAGTAAATGGCTTCCGCCGCC / GTAACGTTAAGTGGATCGTCGACCTAAGCGCGATCCATCAAGTC	Cloning *cre1* cDNA (1158 bp) for fusion to *GFP*
CreAup-F/R	ACGAGCTGTACAAGTAACCCGGGGCGGTGTTGACTCGTTGA / TGGCTGCAGGTCGACGGATCCGAGAGCGTGCGTTGTAATG	Cloning *cre1* 5′ fragment (1520 bp) for recombination/deletion
CreAdn-F/R	GACCCATGGCTCGAGTCTAGAAGGAACGAATTAGGGACGAA / GGTGGTGGTGGCTAGCGTTAACGGGCACGGCACTGAAGTA	Cloning *cre1* 3′ fragment (1521 bp) for recombination/deletion
CreAcm-F/R	ATCCGTCGACCTGCAGCCAAGCTT CATCCATCTAGCCATCCAT / ACACTAGTCAGATCTTCTAGTGT TGTCTGACCGACGCTTTC	Cloning full-length *cre1* sequence for complementation (3639 bp)
pCreA-F/R	GCAATCAGGTCCTCATCC / TCTGACCGACGCTTTCTA	PCR detecting *cre1*
qCreA-F/R	AGTGCCCTCTGTGCGATAAG / ACCGCTGTGGGCATTGTAG	qPCR detecting *cre1*
qACT-F/R	GGCAACATTGTCATGTCTGG / TTTGCTGGAAGGTGGATAGG	qPCR detecting β-actin gene

[a] Underlined regions denote restriction enzyme sites for *cre1* fused to N-terminus of *GFP* (*Bam*HI/*Xma*I), homogenous recombination of the *bar*-separated 5′ and 3′ fragments to delete *cre1* (*Eco*RI/*Hin*dIII and *Xba*I/*Hpa*I), or the gateway exchange fragments for targeted gene complementation.

[b] PCR detection aimed at the respective fragments of 2560 bp for WT and 1808 bp for Δ*cre1*, and both fragments for Δ*cre1::cre1*.

Table S2. Paired primers used for transcriptional profiling of genes for insight into altered phenotypes of Δ*cre1* and/or validity of its transcriptome in *B. bassiana*.

Gene	Tag locus*	Annotation	Sequences (5′–3′) of paired primers
**Involved in asexual development (conidiation)**			
brlA	BBA_07544	Developmental activator BrlA	GATGGATGACAAGTGCGATG / AAACTCGCACGAGAAACGAT
abaA	BBA_00300	Developmental activator AbaA	GCAAGTCTCCAGCCATAT / CTCCTCTTCGTCATAGTAGTC
wetA	BBA_06126	Developmental activator WetA	ATGCGGTACTACAGCCAAGG / GAGTTCCTGCTGGCTACTGG
vosA	BBA_01023	Developmental activator VosA	ACTCATGGGCTCATTGGTGG / CCGGCAAGAGAGATCCGAAA
frq1	BBA_01528	Frequency protein Frq1	AACTCAATGGCATCAGAT / TATCCTTGTCCGTCATAG
vvd	BBA_02876	Blue-light receptor VVD	ATTGCCAACTACAAGAAGA / GGAAGCCAACAGAGTATT
**Involved in conidial hydrophobicity and adherence**			
hyd1	BBA_03015	Class I hydrophobin Hyd1	ATGGTGGAAAGGATCTGCAC / TGGGAAAGAAGACCATCAGC
Hyd2	BBA_06599	Class II hydrophobin Hyd2	TGTCAAGACTGGCGACATTT / ATTGGGACAAGCTGGTTGAG
hyd3	BBA_00530	Hydrophobin-like protein Hyd3	CTGGCCACCCTACTTCTGTC / TCTGGCTAGGGGTAGAGCAA
hyd4	BBA_03071	Hydrophobin-like protein Hyd4	AGTGCTGTGCCACTGACATC / GGGGTCATGCAAAAGAGACT
hyd5	BBA_02999	Hydrophobin-like protein Hyd5	GAGGCTCGCACTGATAAAGC / CAACCTTGGCACAAATTTCC
**Involved in antioxidant response**			
cat1	BBA_06186	Spore-specific catalase CatA	CAACAACATCCCCGTCTTCT / ACACCAAATCCCTGCATCAT
cat2	BBA_05603	Secreted catalase CatB	CTCGTACTTGGACACGCAGA / TTGTTGAGGGTGTTTGGTGA
cat3	BBA_09109	Cytoplasmic catalase CatC	CGCACAAGAGAACCTTCACA / AATGGTTTGTGTGTCGCTGA
cat4	BBA_09760	Secreted peroxidase/catalase CatD	TCTCTGTCCTGGGCTGATCT / CTTGCTGGGGACAAACTCAT
cat5	BBA_09338	Peroxisomal catalase CatP	CAAGGATTCTTCTGGCAAGC / AGCAATGAGAGCAACGGTCT
cat6	BBA_06567	Catalase-like protein, heme-dependent	TTTCCGTGAGGACATTCACA / AGGACTTTTCGTCCCCATCT
sod1	BBA_02311	Cytosolic Cu/ZnSOD	GCGGCTTCCACATCCACACCTTTG / GGTCCAGCGTTGCCAGTCTTGAG
sod2	BBA_09706	Cytosolic MnSOD	CCAGTGTTTGGCATTGACATG / TCAGCCGTCTTCCAGTTGATG
sod3	BBA_09382	Mitochondrial MnSOD	TCTCCGGCAAGATTATGGAGC / TTGGCGTCATTCTTGGCCT
sod4	BBA_04317	Mitochondrial FeSOD	CGAGATGGTCCTTACGGCTTCAG / GCTCCCAGGTGTTGAGGCATAG
sod5	BBA_01984	Cell wall-anchored Cu/ZnSOD	CGGCGACCTCAGCGGCAAGTAC / GCCAGCAACAACAGGGACCGTAGG
**Involved in cell wall composition and transcriptional regulation**			
cfp	BBA_02121	Cysteine-free protein CFP	ATGAAGTTCTCATCTGCTCTCCTTG / AGGCGGTTGGCAATATCGT
mkk1	BBA_01095	MAP kinase kinase 1 Mkk1	GGTGAAGCCAACACCTTCAT / GCAAGTCAATCAGACCAGCA
CWP	BBA_09174	cell wall protein CWP	CTCTGAACATGCCCGGAGTT / CCAAAGGGACCAGCGAAA
TF1	BBA_08967	fungal zinc cluster transcription factor	CTCTAGAGTTCTCGACGAGCAGC/GCCCTCGTATCCGTCCTGTTG
TF2	BBA_05290	fungal specific transcription factor	ATAATGACGACTTGCGGGCTAA /GGATTGGTGGGTGAGTTGGAC

* Gene accession codes of *B. bassiana* genome under the NCBI accession NL_ADAH00000000.

Table S3. A list of 1881 genes dysregulated in the Δ*cre1* mutant of *B. bassiana*.

Gene_ID	Genomic tag_locus	Δ*cre1* vs. WT			Regu-lated	Annotation
		log_2 *R*	*p* value	*q* value		
19892792	BBA_09780	-18.52	3.30E-129	5.38E-127	down	hypothetical protein BBA_09780
19891689	BBA_08677	-15.50	9.93E-36	1.99E-34	down	hypothetical protein BBA_08677
19891196	BBA_08184	-15.30	1.94E-56	6.73E-55	down	glutathione-s-transferase
19893112	BBA_10100	-14.83	4.20E-51	1.27E-49	down	hypothetical protein BBA_10100
19889278	BBA_06266	-14.20	7.93E-57	2.80E-55	down	Cytochrome P450 CYP570H1
19889649	BBA_06637	-14.07	1.10E-50	3.27E-49	down	hypothetical protein BBA_06637
19892923	BBA_09911	-12.81	1.68E-33	3.11E-32	down	hypothetical protein BBA_09911
19885642	BBA_02630	-12.23	1.10E-76	6.19E-75	down	bassianolide nonribosomal peptide synthetase
19890306	BBA_07294	-11.96	0.00E+00	0.00E+00	down	protease S8 tripeptidyl peptidase I (cln2)
19888148	BBA_05136	-10.45	0.00E+00	0.00E+00	down	carbon catabolite repressor
19890303	BBA_07291	-10.10	0.00E+00	0.00E+00	down	peptidase A4 family protein
19891234	BBA_08222	-9.98	6.54E-37	1.35E-35	down	nonribosomal peptide synthase, putative
19893262	BBA_10250	-9.65	3.40E-55	1.16E-53	down	hypothetical protein BBA_10250
19891192	BBA_08180	-9.48	0.00E+00	0.00E+00	down	MFS multidrug resistance transporter, putative
19891484	BBA_08472	-8.79	3.73E-82	2.43E-80	down	steroid monooxygenase (CpmA), putative
19889580	BBA_06568	-8.66	3.42E-152	7.33E-150	down	hypothetical protein BBA_06568
19892699	BBA_09687	-8.63	2.21E-164	5.50E-162	down	hypothetical protein BBA_09687
19892788	BBA_09776	-8.26	8.98E-228	5.65E-225	down	hypothetical protein BBA_09776
19886646	BBA_03634	-8.22	1.25E-186	4.22E-184	down	hypothetical protein BBA_03634
19885425	BBA_02413	-8.22	7.42E-60	2.83E-58	down	hypothetical protein BBA_02413
19885469	BBA_02457	-8.17	9.97E-212	5.54E-209	down	agglutinin isolectin VI precursor
19891803	BBA_08791	-8.16	0.00E+00	0.00E+00	down	alkaline serine protease AorO
19890649	BBA_07637	-8.05	8.44E-156	1.90E-153	down	hypothetical protein BBA_07637
19892188	BBA_09176	-8.01	1.67E-219	9.85E-217	down	alkaline serine protease AorO
19889313	BBA_06301	-7.96	4.05E-71	2.04E-69	down	hypothetical protein BBA_06301
19889591	BBA_06579	-7.84	8.16E-110	8.75E-108	down	hypothetical protein BBA_06579
19888236	BBA_05224	-7.64	7.15E-62	2.89E-60	down	protein phosphatase 2C
19887217	BBA_04205	-7.56	6.72E-178	2.12E-175	down	major facilitator superfamily transporter
19890517	BBA_07505	-7.48	1.90E-170	5.12E-168	down	peptidase A4 family protein
19892546	BBA_09534	-7.26	5.90E-53	1.88E-51	down	hypothetical protein BBA_09534
19893111	BBA_10099	-7.22	1.45E-66	6.41E-65	down	hypothetical protein BBA_10099
19890652	BBA_07640	-7.11	1.66E-161	4.02E-159	down	tyrosinase-like protein
19890702	BBA_07690	-7.10	8.95E-107	9.49E-105	down	hypothetical protein BBA_07690
19890648	BBA_07636	-6.98	9.50E-68	4.40E-66	down	C6 transcription factor
19886825	BBA_03813	-6.60	1.34E-208	6.31E-206	down	late sexual development protein
19891730	BBA_08718	-6.58	3.67E-44	9.30E-43	down	ankyrin repeat protein
19886647	BBA_03635	-6.52	1.46E-229	9.82E-227	down	IDI-2 precursor
19884195	BBA_01183	-6.31	1.20E-30	2.04E-29	down	protease inhibitor (Tfs1)
19892567	BBA_09555	-6.20	4.51E-82	2.92E-80	down	hypothetical protein BBA_09555
19892091	BBA_09079	-6.19	1.30E-66	5.81E-65	down	major facilitator superfamily protein
19892308	BBA_09296	-6.05	1.22E-112	1.35E-110	down	hypothetical protein BBA_09296
19883095	BBA_00083	-5.80	1.29E-114	1.48E-112	down	hypothetical protein BBA_00083
19891804	BBA_08792	-5.66	1.68E-177	5.10E-175	down	DUF300 domain protein, putative
19890626	BBA_07614	-5.57	1.46E-38	3.18E-37	down	hypothetical protein BBA_07614
19884913	BBA_01901	-5.55	1.35E-49	3.91E-48	down	RBP protein
19891758	BBA_08746	-5.54	1.42E-91	1.22E-89	down	glutathione-S-transferase theta, GST
19888192	BBA_05180	-5.49	2.30E-176	6.78E-174	down	hypothetical protein BBA_05180
19891233	BBA_08221	-5.42	9.10E-67	4.11E-65	down	4-coumarate-CoA ligase
19890651	BBA_07639	-5.42	5.24E-120	6.69E-118	down	hypothetical protein BBA_07639
19885938	BBA_02926	-5.37	1.54E-184	5.03E-182	down	cupin domain protein
19891384	BBA_08372	-5.32	1.53E-207	6.86E-205	down	hypothetical protein BBA_08372
19886719	BBA_03707	-5.29	1.26E-24	1.72E-23	down	multiple ankyrin repeats single kh domain protein
19886794	BBA_03782	-5.28	1.53E-166	3.91E-164	down	putative hydrolase
19889451	BBA_06439	-5.18	2.98E-102	3.02E-100	down	CipC protein
19886828	BBA_03816	-5.18	4.42E-74	2.36E-72	down	lectin-like protein
19887987	BBA_04975	-5.14	3.62E-211	1.90E-208	down	AMP-binding enzyme

19890650	BBA_07638	-5.12	3.79E-68	1.80E-66	down	hypothetical protein BBA_07638
19886483	BBA_03471	-5.10	4.56E-41	1.07E-39	down	
19884249	BBA_01237	-5.07	3.29E-193	1.24E-190	down	Cytochrome P450 CYP620C2
19890679	BBA_07667	-5.07	9.30E-51	2.79E-49	down	reverse transcriptase
19889933	BBA_06921	-5.02	3.14E-122	4.36E-120	down	tripeptidyl peptidase A
19887385	BBA_04373	-4.98	8.78E-34	1.64E-32	down	chalcone synthase B
19888017	BBA_05005	-4.97	8.49E-61	3.36E-59	down	hypothetical protein BBA_05005
19889600	BBA_06588	-4.93	1.66E-95	1.51E-93	down	hypothetical protein BBA_06588
19885639	BBA_02627	-4.92	1.01E-66	4.54E-65	down	hypothetical protein BBA_02627
19892621	BBA_09609	-4.88	1.68E-91	1.43E-89	down	hypothetical protein BBA_09609
19886228	BBA_03216	-4.88	5.61E-95	4.99E-93	down	helix-turn-helix domain-containing protein
19888186	BBA_05174	-4.83	3.44E-193	1.25E-190	down	hypothetical protein BBA_05174
19883029	BBA_00017	-4.81	7.63E-45	1.97E-43	down	ABC transporter
19887956	BBA_04944	-4.76	8.30E-138	1.54E-135	down	phytoene synthase/lycopene cyclase
19892311	BBA_09299	-4.75	2.01E-60	7.77E-59	down	sarcosine oxidase
19886458	BBA_03446	-4.70	4.53E-27	6.77E-26	down	MFS transporter
19891488	BBA_08476	-4.69	1.33E-208	6.31E-206	down	serine carboxypeptidase S28
19887667	BBA_04655	-4.65	1.85E-71	9.45E-70	down	hypothetical protein BBA_04655
19893373	BBA_10362	-4.61	2.84E-32	5.08E-31	down	PDZ domain-containing protein
19893334	BBA_10322	-4.53	2.48E-34	4.68E-33	down	hypothetical protein BBA_10322
19884826	BBA_01814	-4.52	1.52E-156	3.49E-154	down	oxidoreductase, 2OG-Fe(II) oxygenase family
19883096	BBA_00084	-4.48	3.29E-50	9.67E-49	down	Peptidase M64, IgA
19891402	BBA_08390	-4.46	9.79E-124	1.40E-121	down	hypothetical protein BBA_08390
19892309	BBA_09297	-4.46	3.24E-30	5.39E-29	down	hypothetical protein BBA_09297
19886895	BBA_03883	-4.44	3.77E-176	1.08E-173	down	phytase, putative
19885641	BBA_02629	-4.42	4.13E-27	6.22E-26	down	Putative conidiospore surface protein
19890302	BBA_07290	-4.41	2.98E-41	7.04E-40	down	hypothetical protein BBA_07290
19884995	BBA_01983	-4.40	8.21E-202	3.37E-199	down	hypothetical protein BBA_01983
19892787	BBA_09775	-4.39	7.08E-119	8.92E-117	down	dimethylaniline monooxygenase
19889358	BBA_06346	-4.36	7.89E-144	1.59E-141	down	oxaloacetate acetylhydrolase
19888185	BBA_05173	-4.35	1.19E-77	6.90E-76	down	Cytochrome P450 CYP52G6
19889368	BBA_06356	-4.34	3.08E-190	1.08E-187	down	hypothetical protein BBA_06356
19888894	BBA_05882	-4.26	1.32E-138	2.50E-136	down	hypothetical protein BBA_05882
19892234	BBA_09222	-4.20	1.94E-130	3.27E-128	down	metallo-beta-lactamase superfamily protein
19886214	BBA_03202	-4.20	6.81E-124	1.00E-121	down	phospholipase C PLC-C
19892577	BBA_09565	-4.19	3.75E-53	1.21E-51	down	hypothetical protein BBA_09565
19886807	BBA_03795	-4.15	3.02E-48	8.53E-47	down	hypothetical protein BBA_03795
19890783	BBA_07771	-4.09	7.21E-71	3.62E-69	down	feebly protein
19890756	BBA_07744	-4.08	9.79E-73	5.14E-71	down	FAD dependent oxidoreductase
19890043	BBA_07031	-4.07	4.10E-42	9.95E-41	down	MFS multidrug transporter
19890863	BBA_07851	-4.05	1.12E-23	1.46E-22	down	hypothetical protein BBA_07851
19891909	BBA_08897	-4.02	8.67E-56	2.97E-54	down	longiborneol synthase
19890625	BBA_07613	-4.01	6.42E-95	5.67E-93	down	Cytochrome P450 CYP684A2
19889493	BBA_06481	-3.99	3.92E-132	6.73E-130	down	hypothetical protein BBA_06481
19891073	BBA_08061	-3.99	5.72E-51	1.73E-49	down	UPF0075 domain protein
19890150	BBA_07138	-3.98	1.30E-95	1.20E-93	down	conidial wall protein
19888187	BBA_05175	-3.97	2.17E-126	3.31E-124	down	LCCL domain-containing protein
19886864	BBA_03852	-3.94	2.70E-105	2.84E-103	down	penicillin-binding protein
19889428	BBA_06416	-3.91	6.21E-40	1.42E-38	down	membrane transporter
19884427	BBA_01415	-3.89	2.30E-70	1.15E-68	down	hypothetical protein BBA_01415
19887401	BBA_04389	-3.89	1.94E-19	2.08E-18	down	ATP synthase F1
19890113	BBA_07101	-3.85	1.54E-81	9.82E-80	down	hypothetical protein BBA_07101
19891383	BBA_08371	-3.84	3.78E-117	4.58E-115	down	hypothetical protein BBA_08371
19892467	BBA_09455	-3.84	1.67E-45	4.36E-44	down	hypothetical protein BBA_09455
19883804	BBA_00792	-3.80	1.18E-132	2.07E-130	down	beta-1, 3 exoglucanase precursor
19890745	BBA_07733	-3.78	8.19E-85	5.77E-83	down	Mating-type protein MAT1-1-1
19890897	BBA_07885	-3.78	3.88E-80	2.38E-78	down	hypothetical protein BBA_07885
19893011	BBA_09999	-3.76	5.28E-65	2.26E-63	down	hypothetical protein BBA_09999
19891259	BBA_08247	-3.75	4.67E-78	2.77E-76	down	carboxypeptidase-like protein
19888365	BBA_05353	-3.75	1.48E-15	1.28E-14	down	class III chitinase ChiA2
19893022	BBA_10010	-3.74	7.17E-36	1.44E-34	down	Cytochrome P450 CYP542B2

19893108	BBA_10096	-3.74	5.12E-45	1.33E-43	down	metalloreductase transmembrane component
19883552	BBA_00540	-3.72	2.60E-85	1.85E-83	down	MFS transporter, putative
19885629	BBA_02617	-3.70	6.33E-39	1.41E-37	down	Ankyrin repeat protein
19886225	BBA_03213	-3.68	6.89E-54	2.25E-52	down	putative aspartic protease
19893322	BBA_10310	-3.67	6.24E-34	1.17E-32	down	hypothetical protein BBA_10310
19887386	BBA_04374	-3.65	1.21E-30	2.05E-29	down	polyketide synthase, putative
19889295	BBA_06283	-3.65	7.71E-70	3.77E-68	down	hypothetical protein BBA_06283
19883540	BBA_00528	-3.65	1.14E-142	2.24E-140	down	short chain dehydrogenase
19886718	BBA_03706	-3.64	2.47E-23	3.18E-22	down	hypothetical protein BBA_03706
19886489	BBA_03477	-3.63	4.06E-23	5.15E-22	down	Cytochrome P450 CYP682H1
19889720	BBA_06708	-3.62	2.59E-104	2.69E-102	down	hypothetical protein BBA_06708
19885130	BBA_02118	-3.59	1.90E-38	4.12E-37	down	phosphatidylcholine-hydrolyzing phospholipase C
19889808	BBA_06796	-3.57	5.72E-42	1.39E-40	down	hypothetical protein BBA_06796
19885167	BBA_02155	-3.57	2.51E-115	2.93E-113	down	Cytochrome P450 CYP6003A1
19883017	BBA_00005	-3.55	3.65E-122	5.00E-120	down	ABC transporter transmembrane region
19892618	BBA_09606	-3.51	2.07E-15	1.78E-14	down	hypothetical protein BBA_09606
19893233	BBA_10221	-3.50	2.48E-56	8.57E-55	down	hypothetical protein BBA_10221
19885414	BBA_02402	-3.47	7.75E-22	9.29E-21	down	hypothetical protein BBA_02402
19892307	BBA_09295	-3.46	1.08E-63	4.51E-62	down	glutathione-dependent formaldehyde-activating enzyme
19883647	BBA_00635	-3.45	2.66E-10	1.58E-09	down	Secretory lipase family protein
19890757	BBA_07745	-3.45	1.09E-68	5.27E-67	down	Glycoside hydrolase, catalytic core
19885408	BBA_02396	-3.41	1.29E-137	2.34E-135	down	NADPH-dependent FMN reductase
19886826	BBA_03814	-3.41	1.12E-62	4.55E-61	down	chitinase-like protein
19890135	BBA_07123	-3.40	1.53E-23	1.98E-22	down	hypothetical protein BBA_07123
19891755	BBA_08743	-3.39	2.16E-127	3.34E-125	down	sarcosine oxidase
19885603	BBA_02591	-3.39	2.04E-95	1.84E-93	down	C6 zinc finger domain protein
19889068	BBA_06056	-3.37	1.06E-116	1.27E-114	down	allantoate permease
19892606	BBA_09594	-3.36	8.80E-28	1.35E-26	down	putative peptidylarginine deiminase
19888845	BBA_05833	-3.34	1.12E-51	3.43E-50	down	major facilitator superfamily transporter
19887962	BBA_04950	-3.34	3.17E-63	1.30E-61	down	nacht and ankyrin domain containing protein
19883628	BBA_00616	-3.34	3.04E-121	4.04E-119	down	glycoside hydrolase family 35
19883266	BBA_00254	-3.34	2.30E-34	4.35E-33	down	hypothetical protein BBA_00254
19887795	BBA_04783	-3.32	4.40E-29	6.99E-28	down	UDP-N-acetylmuramate--L-alanine ligase
19886484	BBA_03472	-3.32	5.57E-77	3.17E-75	down	Ankyrin repeat protein
19885234	BBA_02222	-3.31	8.83E-39	1.96E-37	down	F-box domain-containing protein
19892473	BBA_09461	-3.29	8.26E-124	1.20E-121	down	methyltransferase-like protein
19892476	BBA_09464	-3.29	1.48E-65	6.48E-64	down	hypothetical protein BBA_09464
19892405	BBA_09393	-3.29	1.17E-54	3.90E-53	down	sugar transport protein
19892791	BBA_09779	-3.26	5.88E-30	9.67E-29	down	hypothetical protein BBA_09779
19886219	BBA_03207	-3.22	3.12E-82	2.05E-80	down	amidase-like protein
19885957	BBA_02945	-3.21	3.88E-68	1.83E-66	down	LysM domain-containing protein
19886645	BBA_03633	-3.21	1.85E-24	2.52E-23	down	hypothetical protein BBA_03633
19888941	BBA_05929	-3.19	1.04E-113	1.18E-111	down	WSC domain-containing protein
19888565	BBA_05553	-3.19	1.33E-66	5.91E-65	down	copper amine oxidase
19893363	BBA_10351	-3.18	1.14E-29	1.85E-28	down	serine/threonine protein kinase Japonica Group
19892404	BBA_09392	-3.16	6.37E-89	5.14E-87	down	Alkaline phosphatase-like protein
19886560	BBA_03548	-3.16	3.95E-35	7.77E-34	down	hypothetical protein BBA_03548
19892720	BBA_09708	-3.14	7.16E-28	1.10E-26	down	hypothetical protein BBA_09708
19889721	BBA_06709	-3.13	4.31E-61	1.72E-59	down	carboxylesterase-like protein
19892564	BBA_09552	-3.13	1.43E-07	6.44E-07	down	hypothetical protein BBA_09552
19892344	BBA_09332	-3.10	9.67E-37	1.99E-35	down	hypothetical protein BBA_09332
19884196	BBA_01184	-3.10	2.62E-53	8.51E-52	down	FAD binding domain protein
19889754	BBA_06742	-3.09	2.53E-112	2.78E-110	down	HET domain-containing protein
19887283	BBA_04271	-3.08	3.36E-79	2.02E-77	down	pheromone receptor
19886831	BBA_03819	-3.08	2.42E-87	1.87E-85	down	l-lactate dehydrogenase
19891516	BBA_08504	-3.07	1.66E-59	6.21E-58	down	hypothetical protein BBA_08504
19892905	BBA_09893	-3.06	1.47E-14	1.21E-13	down	hypothetical protein BBA_09893
19883537	BBA_00525	-3.03	1.14E-72	5.94E-71	down	GPI anchored cell wall protein
19891514	BBA_08502	-3.02	5.74E-87	4.34E-85	down	hypothetical protein BBA_08502
19887701	BBA_04689	-3.02	2.06E-24	2.78E-23	down	hypothetical protein BBA_04689
19889486	BBA_06474	-3.02	1.41E-44	3.62E-43	down	cucumopine synthase

19885134	BBA_02122	-3.01	2.75E-71	1.39E-69	down	hypothetical protein BBA_02122
19890801	BBA_07789	-3.01	6.12E-56	2.10E-54	down	hypothetical protein BBA_07789
19891195	BBA_08183	-3.01	2.24E-54	7.40E-53	down	laccase 2
19889340	BBA_06328	-2.99	1.59E-87	1.24E-85	down	methyltransferase-like protein
19883805	BBA_00793	-2.99	7.37E-68	3.45E-66	down	hypothetical protein BBA_00793
19893279	BBA_10267	-2.98	6.65E-77	3.76E-75	down	amidohydrolase-like protein
19889485	BBA_06473	-2.98	1.10E-93	9.50E-92	down	Cytochrome P450 CYP684A2
19885236	BBA_02224	-2.98	6.90E-70	3.40E-68	down	hypothetical protein BBA_02224
19892538	BBA_09526	-2.98	2.63E-12	1.82E-11	down	MFS transporter
19892439	BBA_09427	-2.96	8.21E-103	8.42E-101	down	serine carboxypeptidase
19891432	BBA_08420	-2.94	1.48E-50	4.38E-49	down	abhydrolase domain-containing protein
19892547	BBA_09535	-2.94	1.01E-50	3.02E-49	down	fatty acid desaturase
19888302	BBA_05290	-2.93	2.65E-46	7.01E-45	down	fungal specific transcription factor
19886194	BBA_03182	-2.93	2.16E-08	1.07E-07	down	hypothetical protein BBA_03182
19887098	BBA_04086	-2.91	1.65E-53	5.36E-52	down	Zn2C6-type transcription factor
19885008	BBA_01996	-2.91	1.33E-70	6.67E-69	down	GPI anchored protein, putative
19892937	BBA_09925	-2.91	6.93E-50	2.02E-48	down	tetratricopeptide repeat domain protein
19890218	BBA_07206	-2.91	1.01E-90	8.39E-89	down	hypothetical protein BBA_07206
19888563	BBA_05551	-2.90	4.31E-83	2.93E-81	down	Beta-lactamase-type transpeptidase
19889394	BBA_06382	-2.89	4.49E-40	1.03E-38	down	hypothetical protein BBA_06382
19887395	BBA_04383	-2.87	3.11E-89	2.55E-87	down	Enoyl-CoA Hydratase family member
19885682	BBA_02670	-2.86	1.06E-46	2.85E-45	down	hypothetical protein BBA_02670
19890755	BBA_07743	-2.86	1.70E-88	1.35E-86	down	serine peptidase, putative
19885168	BBA_02156	-2.85	7.76E-87	5.77E-85	down	sporulation associated protein
19892585	BBA_09573	-2.85	2.00E-66	8.84E-65	down	Cytochrome P450 CYP542B3
19889752	BBA_06740	-2.85	2.40E-83	1.64E-81	down	hypothetical protein BBA_06740
19892208	BBA_09196	-2.83	1.06E-41	2.55E-40	down	hypothetical protein BBA_09196
19885245	BBA_02233	-2.82	7.67E-13	5.57E-12	down	Glycoside hydrolase, catalytic core
19890098	BBA_07086	-2.82	6.77E-68	3.18E-66	down	hypothetical protein BBA_07086
19892310	BBA_09298	-2.81	3.45E-48	9.66E-47	down	Zn(II)2Cys6 transcription factor
19885463	BBA_02451	-2.80	2.88E-86	2.09E-84	down	oligopeptide transporter
19891185	BBA_08173	-2.79	5.64E-12	3.83E-11	down	hypothetical protein BBA_08173
19891255	BBA_08243	-2.78	2.00E-77	1.15E-75	down	calcineurin-like phosphoesterase
19891433	BBA_08421	-2.77	8.33E-04	2.08E-03	down	volvatoxin A2 precursor
19888664	BBA_05652	-2.77	7.59E-53	2.40E-51	down	hypothetical protein BBA_05652
19891629	BBA_08617	-2.76	3.36E-34	6.30E-33	down	hypothetical protein BBA_08617
19892738	BBA_09726	-2.76	4.42E-15	3.76E-14	down	2-dehydropantoate 2-reductase, putative
19890066	BBA_07054	-2.75	6.30E-59	2.32E-57	down	C6 zinc finger domain-containing protein
19891447	BBA_08435	-2.74	7.15E-47	1.94E-45	down	FAD binding domain-containing protein
19891715	BBA_08703	-2.73	2.14E-32	3.86E-31	down	hypothetical protein BBA_08703
19887302	BBA_04290	-2.73	8.88E-51	2.67E-49	down	hypothetical protein BBA_04290
19892474	BBA_09462	-2.72	6.06E-87	4.54E-85	down	methyltransferase-like protein
19886448	BBA_03436	-2.71	2.71E-22	3.32E-21	down	hypothetical protein BBA_03436
19890126	BBA_07114	-2.71	3.93E-87	3.02E-85	down	aldo/keto reductase
19887988	BBA_04976	-2.71	3.72E-68	1.78E-66	down	enoyl-CoA hydratase/isomerase family protein
19886459	BBA_03447	-2.70	2.84E-34	5.36E-33	down	extracellular protein
19889487	BBA_06475	-2.70	2.30E-81	1.45E-79	down	adenylate-forming enzyme AfeA
19884130	BBA_01118	-2.68	3.30E-54	1.08E-52	down	hypothetical protein BBA_01118
19892708	BBA_09696	-2.67	7.81E-69	3.78E-67	down	tetratricopeptide repeat domain protein
19893182	BBA_10170	-2.67	2.98E-82	1.97E-80	down	RNA interference and silencing protein (Qde2)
19886774	BBA_03762	-2.67	4.18E-21	4.88E-20	down	protein kinase subdomain-containing protein
19886292	BBA_03280	-2.67	1.41E-76	7.87E-75	down	peroxin 11C
19885351	BBA_02339	-2.66	1.93E-38	4.17E-37	down	taurine catabolism dioxygenase TauD
19893260	BBA_10248	-2.66	1.01E-13	7.83E-13	down	aminotransferase class-III
19890769	BBA_07757	-2.65	2.48E-18	2.50E-17	down	maleylacetoacetate isomerase
19888404	BBA_05392	-2.65	4.92E-67	2.26E-65	down	O-methyltransferase-like protein
19885714	BBA_02702	-2.64	1.55E-49	4.49E-48	down	salicylate hydroxylase
19890142	BBA_07130	-2.63	1.32E-40	3.06E-39	down	kinesin light chain 1 and
19892298	BBA_09286	-2.63	4.81E-36	9.77E-35	down	oxygenase-like protein
19886921	BBA_03909	-2.62	8.70E-78	5.14E-76	down	filamentous hemagglutinin / adhesin
19886320	BBA_03308	-2.62	1.16E-10	7.13E-10	down	fatty acid hydroxylase superfamily protein

19892375	BBA_09363	-2.62	4.97E-11	3.16E-10	down	hypothetical protein BBA_09363
19888721	BBA_05709	-2.61	8.15E-38	1.74E-36	down	hypothetical protein BBA_05709
19884930	BBA_01918	-2.61	4.97E-77	2.85E-75	down	
19886218	BBA_03206	-2.60	2.81E-52	8.63E-51	down	acid phosphatase
19886234	BBA_03222	-2.60	1.48E-31	2.57E-30	down	serine/threonine protein kinase
19891478	BBA_08466	-2.60	5.17E-15	4.35E-14	down	hypothetical protein BBA_08466
19892778	BBA_09766	-2.58	5.54E-27	8.25E-26	down	hypothetical protein BBA_09766
19889770	BBA_06758	-2.58	1.78E-64	7.59E-63	down	Na/K ATPase alpha 1 subunit, putative
19892855	BBA_09843	-2.57	2.49E-11	1.63E-10	down	hypothetical protein BBA_09843
19891232	BBA_08220	-2.57	2.12E-25	2.99E-24	down	trichothecene 3-O-acetyltransferase
19883863	BBA_00851	-2.57	6.41E-24	8.43E-23	down	small oligopeptide transporter, OPT family
19885300	BBA_02288	-2.56	3.31E-29	5.29E-28	down	homeobox domain-containing protein
19890828	BBA_07816	-2.56	5.25E-75	2.87E-73	down	major facilitator superfamily transporter
19885746	BBA_02734	-2.56	1.84E-73	9.73E-72	down	formate dehydrogenase
19887469	BBA_04457	-2.56	3.33E-79	2.01E-77	down	hypothetical protein BBA_04457
19893074	BBA_10062	-2.56	5.60E-29	8.86E-28	down	hypothetical protein BBA_10062
19893319	BBA_10307	-2.56	8.89E-12	5.97E-11	down	hypothetical protein BBA_10307
19885396	BBA_02384	-2.55	2.20E-79	1.34E-77	down	Cytochrome P450 CYP542B3
19889445	BBA_06433	-2.55	2.90E-80	1.80E-78	down	hypothetical protein BBA_06433
19887394	BBA_04382	-2.54	1.06E-74	5.71E-73	down	xylitol dehydrogenase
19892857	BBA_09845	-2.54	1.90E-12	1.34E-11	down	hypothetical protein BBA_09845
19885494	BBA_02482	-2.54	7.02E-55	2.37E-53	down	carboxypeptidase Y
19891072	BBA_08060	-2.54	6.12E-21	7.09E-20	down	MFS monosaccharide transporter, putative
19889578	BBA_06566	-2.50	1.44E-06	5.72E-06	down	ankyrin repeat protein
19887627	BBA_04615	-2.49	2.01E-52	6.21E-51	down	hypothetical protein BBA_04615
19888461	BBA_05449	-2.48	1.33E-63	5.49E-62	down	metabolite transport protein GIT1
19892580	BBA_09568	-2.47	8.57E-09	4.44E-08	down	pol-like protein
19886835	BBA_03823	-2.47	2.61E-13	1.96E-12	down	naphthalene 1,2-dioxygenase subunit alpha
19891658	BBA_08646	-2.46	4.88E-37	1.01E-35	down	hypothetical protein BBA_08646
19889590	BBA_06578	-2.46	3.74E-14	2.97E-13	down	heterokaryon incompatibility protein
19889484	BBA_06472	-2.45	2.51E-43	6.29E-42	down	Cytochrome P450 CYP5099A1
19887096	BBA_04084	-2.45	5.16E-59	1.90E-57	down	putative aspartic protease
19885429	BBA_02417	-2.44	7.21E-48	2.01E-46	down	secreted aspartic proteinase
19889570	BBA_06558	-2.42	2.77E-16	2.49E-15	down	ABC transporter, putative
19889309	BBA_06297	-2.42	1.17E-38	2.57E-37	down	chitinase-like protein
19887220	BBA_04208	-2.42	3.35E-59	1.24E-57	down	hypothetical protein BBA_04208
19888101	BBA_05089	-2.41	5.85E-57	2.08E-55	down	putative signal peptide-containing protein
19883871	BBA_00859	-2.41	5.78E-67	2.63E-65	down	Putative Zn(II)2Cys6 transcription factor
19887675	BBA_04663	-2.40	3.16E-59	1.17E-57	down	capsular associated protein
19891746	BBA_08734	-2.40	8.96E-43	2.22E-41	down	cyanide hydratase
19892301	BBA_09289	-2.39	7.70E-53	2.43E-51	down	Pectin lyase fold/virulence factor
19893117	BBA_10105	-2.39	1.43E-54	4.76E-53	down	nonribosomal peptide synthase
19893376	BBA_10366	-2.39	9.35E-24	1.22E-22	down	PDZ domain-containing protein
19892856	BBA_09844	-2.39	2.32E-15	1.99E-14	down	vacuolar calcium ion transporter /H(+) exchanger
19891253	BBA_08241	-2.39	3.14E-54	1.03E-52	down	general alpha-glucoside permease
19892319	BBA_09307	-2.39	5.71E-50	1.67E-48	down	acidic chitinase
19887774	BBA_04762	-2.38	3.51E-30	5.82E-29	down	hypothetical protein BBA_04762
19888172	BBA_05160	-2.37	6.88E-69	3.35E-67	down	glycolipid anchored surface protein
19884817	BBA_01805	-2.37	8.00E-25	1.11E-23	down	MFS transporter
19891553	BBA_08541	-2.37	5.10E-28	7.88E-27	down	short-chain dehydrogenase
19883474	BBA_00462	-2.37	5.62E-53	1.81E-51	down	APSES transcription factor Xbp1
19886806	BBA_03794	-2.36	1.92E-06	7.44E-06	down	hypothetical protein BBA_03794
19885710	BBA_02698	-2.34	2.96E-66	1.30E-64	down	Cytochrome P450 CYP5080B3
19890006	BBA_06994	-2.34	1.63E-60	6.36E-59	down	putative apoptosis-inducing factor (AIF)
19887174	BBA_04162	-2.33	2.91E-47	7.95E-46	down	ZIP zinc/iron transporter
19883925	BBA_00913	-2.33	1.73E-41	4.14E-40	down	hypothetical protein BBA_00913
19886839	BBA_03827	-2.33	6.99E-10	4.02E-09	down	Riboflavin synthase-like beta-barrel
19885663	BBA_02651	-2.31	3.71E-64	1.57E-62	down	LPXTG-domain-containing protein
19892247	BBA_09235	-2.31	1.58E-55	5.39E-54	down	putative dual specificity protein kinase pom1
19886844	BBA_03832	-2.31	6.58E-63	2.69E-61	down	PAN domain containing protein
19887999	BBA_04987	-2.29	8.65E-57	3.05E-55	down	hypothetical protein BBA_04987

19887421	BBA_04409	-2.28	4.10E-11	2.62E-10	down	hypothetical protein BBA_04409
19890347	BBA_07335	-2.27	1.51E-15	1.30E-14	down	Cytochrome P450 CYP655C1
19891193	BBA_08181	-2.27	1.46E-27	2.23E-26	down	fungal specific transcription factor
19888189	BBA_05177	-2.26	8.34E-39	1.85E-37	down	enoyl reductase
19888373	BBA_05361	-2.26	7.05E-55	2.37E-53	down	amidohydrolase-like protein
19885541	BBA_02529	-2.26	3.31E-58	1.20E-56	down	DNase1 protein
19887011	BBA_03999	-2.26	1.92E-32	3.47E-31	down	hypothetical protein BBA_03999
19891146	BBA_08134	-2.26	1.93E-10	1.16E-09	down	hypothetical protein BBA_08134
19886404	BBA_03392	-2.26	1.14E-59	4.29E-58	down	membrane protein, putative
19892797	BBA_09785	-2.26	1.83E-56	6.39E-55	down	3-hydroxybutyryl-CoA dehydratase
19889756	BBA_06744	-2.25	5.11E-60	1.96E-58	down	hypothetical protein BBA_06744
19891513	BBA_08501	-2.25	8.46E-60	3.21E-58	down	putative SAM-dependent methyltransferase
19886940	BBA_03928	-2.25	4.80E-31	8.18E-30	down	hypothetical protein BBA_03928
19883902	BBA_00890	-2.24	1.79E-20	2.02E-19	down	Cytochrome P450 CYP5060A1
19885009	BBA_01997	-2.24	3.45E-31	5.91E-30	down	hypothetical protein BBA_01997
19887938	BBA_04926	-2.24	1.43E-37	3.04E-36	down	steroid monooxygenase
19890343	BBA_07331	-2.24	1.77E-57	6.37E-56	down	N,O-diacetyl muramidase, putative
19885107	BBA_02095	-2.24	7.85E-15	6.56E-14	down	hypothetical protein BBA_02095
19887462	BBA_04450	-2.23	1.63E-31	2.83E-30	down	aspartic-type endopeptidase
19888009	BBA_04997	-2.23	1.37E-39	3.10E-38	down	hypothetical protein BBA_04997
19890686	BBA_07674	-2.23	4.01E-49	1.16E-47	down	Peptidase S33, tripeptidyl-peptidase
19885347	BBA_02335	-2.22	8.17E-62	3.28E-60	down	Cytochrome P450 CYP584E2
19889729	BBA_06717	-2.22	2.93E-16	2.64E-15	down	putative YFW family protein 5
19886777	BBA_03765	-2.22	2.14E-16	1.94E-15	down	thiamin biosynthesis protein (Thi-4)
19887957	BBA_04945	-2.22	2.57E-32	4.62E-31	down	phytoene dehydrogenase
19891915	BBA_08903	-2.22	6.55E-15	5.50E-14	down	hypothetical protein BBA_08903
19885468	BBA_02456	-2.21	1.03E-22	1.29E-21	down	carotenoid ester lipase precursor
19887702	BBA_04690	-2.21	8.71E-60	3.29E-58	down	Na,H/K antiporter P-type ATPase
19886233	BBA_03221	-2.21	2.08E-42	5.08E-41	down	arginase-like protein
19892581	BBA_09569	-2.21	1.58E-14	1.30E-13	down	hypothetical protein BBA_09569
19884511	BBA_01499	-2.20	1.35E-48	3.86E-47	down	nitrate assimilation regulatory protein nirA
19889316	BBA_06304	-2.20	9.24E-47	2.49E-45	down	lipocalin-like domain-containing protein
19886841	BBA_03829	-2.20	1.18E-51	3.59E-50	down	peptidase family M3
19885464	BBA_02452	-2.19	4.94E-48	1.38E-46	down	ferulic acid esterase (FaeA), putative
19886130	BBA_03118	-2.18	2.61E-37	5.50E-36	down	von Willebrand factor type A domain-containing protein
19892626	BBA_09614	-2.17	2.72E-41	6.45E-40	down	hypothetical protein BBA_09614
19888837	BBA_05825	-2.17	9.83E-27	1.45E-25	down	secreted glucosidase
19883856	BBA_00844	-2.17	4.10E-46	1.08E-44	down	fungal specific transcription factor
19887237	BBA_04225	-2.17	6.26E-53	1.99E-51	down	alpha-tubulin
19887670	BBA_04658	-2.16	1.90E-57	6.78E-56	down	methyltransferase-like protein
19888303	BBA_05291	-2.16	1.56E-15	1.35E-14	down	thiol-specific monooxygenase
19889753	BBA_06741	-2.16	1.50E-20	1.70E-19	down	hypothetical protein BBA_06741
19885845	BBA_02833	-2.16	2.96E-44	7.53E-43	down	phosphoenolpyruvate carboxykinase
19892165	BBA_09153	-2.15	3.78E-57	1.35E-55	down	subtilisin-like serine protease PR1C
19887839	BBA_04827	-2.15	4.40E-35	8.62E-34	down	peptide synthetase
19883393	BBA_00381	-2.15	6.88E-42	1.66E-40	down	pH-response regulator protein palF/RIM8
19887982	BBA_04970	-2.15	7.74E-58	2.79E-56	down	dihydroxyacetone kinase
19885777	BBA_02765	-2.15	1.08E-29	1.76E-28	down	DJ-1/PfpI family protein
19890571	BBA_07559	-2.14	1.13E-47	3.11E-46	down	putative aspartate protease
19888821	BBA_05809	-2.13	5.61E-27	8.33E-26	down	MFS allantoate transporter, putative
19892573	BBA_09561	-2.13	7.18E-28	1.11E-26	down	F-box domain, Skp2
19887843	BBA_04831	-2.13	2.02E-16	1.84E-15	down	hypothetical protein BBA_04831
19892586	BBA_09574	-2.13	7.55E-30	1.24E-28	down	hypothetical protein BBA_09574
19887461	BBA_04449	-2.12	9.65E-25	1.33E-23	down	helix-loop-helix DNA-binding domain-containing protein
19888814	BBA_05802	-2.12	7.20E-53	2.28E-51	down	ankyrin repeat protein
19889067	BBA_06055	-2.12	6.04E-32	1.07E-30	down	dihydroflavonal-4-reductase protein
19883676	BBA_00664	-2.12	7.02E-23	8.84E-22	down	
19892790	BBA_09778	-2.11	7.38E-32	1.29E-30	down	hypothetical protein BBA_09778
19887078	BBA_04066	-2.11	2.04E-29	3.28E-28	down	PLC-like phosphodiesterase
19893324	BBA_10312	-2.11	1.17E-22	1.46E-21	down	hypothetical protein BBA_10312
19883374	BBA_00362	-2.11	6.57E-39	1.46E-37	down	hypothetical protein BBA_00362

19890816	BBA_07804	-2.11	2.38E-03	5.38E-03	down	putative salivary secreted peptide
19889077	BBA_06065	-2.11	9.18E-09	4.75E-08	down	hypothetical protein BBA_06065
19891485	BBA_08473	-2.11	1.17E-17	1.14E-16	down	siderophore iron transporter mirA
19889755	BBA_06743	-2.11	1.22E-27	1.86E-26	down	hypothetical protein BBA_06743
19891386	BBA_08374	-2.10	5.35E-46	1.41E-44	down	glyoxalase-like protein
19887990	BBA_04978	-2.10	9.11E-39	2.01E-37	down	tripeptidyl peptidase precursor
19889583	BBA_06571	-2.10	2.51E-44	6.40E-43	down	acyl-CoA dehydrogenase domain-containing protein
19889132	BBA_06120	-2.10	2.04E-44	5.23E-43	down	C2H2 type zinc finger domain protein
19893021	BBA_10009	-2.10	3.68E-19	3.88E-18	down	6-hydroxy-D-nicotine oxidase
19886979	BBA_03967	-2.10	2.65E-19	2.81E-18	down	hypothetical protein BBA_03967
19889684	BBA_06672	-2.09	2.54E-12	1.76E-11	down	hypothetical protein BBA_06672
19886678	BBA_03666	-2.08	4.68E-18	4.64E-17	down	ABC bile acid transporter, putative
19886129	BBA_03117	-2.08	3.88E-22	4.71E-21	down	Acyl-CoA N-acyltransferase
19883071	BBA_00059	-2.08	9.58E-18	9.37E-17	down	FAD binding domain containing protein
19889093	BBA_06081	-2.08	2.69E-39	6.07E-38	down	allantoate permease
19883601	BBA_00589	-2.08	3.64E-36	7.41E-35	down	pyruvate dehydrogenase
19883534	BBA_00522	-2.08	3.12E-30	5.20E-29	down	Urease, alpha subunit
19889233	BBA_06221	-2.06	9.71E-35	1.88E-33	down	putative HFM1/MER3 protein
19884393	BBA_01381	-2.06	1.32E-08	6.74E-08	down	hypothetical protein BBA_01381
19891729	BBA_08717	-2.06	1.03E-16	9.57E-16	down	hypothetical protein BBA_08717
19886969	BBA_03957	-2.05	8.40E-48	2.33E-46	down	integral membrane protein
19892616	BBA_09604	-2.05	5.75E-04	1.48E-03	down	synaptobrevin-like protein
19885069	BBA_02057	-2.04	3.18E-20	3.55E-19	down	heat shock protein 30
19883432	BBA_00420	-2.04	1.93E-31	3.34E-30	down	hypothetical protein BBA_00420
19886630	BBA_03618	-2.03	4.76E-21	5.55E-20	down	hypothetical protein BBA_03618
19890314	BBA_07302	-2.03	3.26E-22	3.98E-21	down	GMP synthase
19892440	BBA_09428	-2.03	9.17E-10	5.21E-09	down	peptidase family M3
19883926	BBA_00914	-2.02	3.63E-41	8.51E-40	down	SMP-30/Gluconolaconase/LRE-like region
19886530	BBA_03518	-2.02	1.76E-20	1.99E-19	down	major facilitator superfamily transporter
19887981	BBA_04969	-2.01	6.37E-29	1.00E-27	down	ribose 5-phosphate isomerase
19887650	BBA_04638	-2.01	5.27E-15	4.43E-14	down	flavohemoglobin-like protein
19885406	BBA_02394	-2.01	1.97E-19	2.10E-18	down	methyltransferase-like protein
19891642	BBA_08630	-2.01	8.19E-30	1.34E-28	down	kinesin family protein
19890400	BBA_07388	-2.01	1.62E-46	4.31E-45	down	Glycoside hydrolase, catalytic core
19892485	BBA_09473	-2.00	1.57E-25	2.22E-24	down	dethiobiotin synthetase
19892908	BBA_09896	-2.00	7.55E-25	1.05E-23	down	Pfs, NACHT, and Ankyrin domain protein
19886837	BBA_03825	-2.00	1.81E-42	4.44E-41	down	Cytochrome P450 CYP6001C8
19891816	BBA_08804	-2.00	2.74E-11	1.79E-10	down	hypothetical protein BBA_08804
19888773	BBA_05761	-2.00	1.73E-47	4.76E-46	down	phosphate permease
19888615	BBA_05603	-1.99	3.03E-48	8.55E-47	down	Catalase-like domain, heme-dependent
19892789	BBA_09777	-1.99	6.56E-10	3.79E-09	down	hypothetical protein BBA_09777
19892934	BBA_09922	-1.97	1.66E-42	4.08E-41	down	hypothetical protein BBA_09922
19890515	BBA_07503	-1.97	1.69E-35	3.36E-34	down	2-C-methyl-D-erythritol 2,4-cyclodiphosphate synthase
19885656	BBA_02644	-1.97	9.36E-35	1.82E-33	down	Ncp1-like protein
19888824	BBA_05812	-1.97	3.11E-41	7.33E-40	down	hexose transporter
19889757	BBA_06745	-1.97	2.95E-33	5.42E-32	down	putative peroxisomal membrane protein
19892783	BBA_09771	-1.96	1.95E-37	4.11E-36	down	FAD dependent oxidoreductase
19883717	BBA_00705	-1.96	3.66E-16	3.27E-15	down	hypothetical protein BBA_00705
19891049	BBA_08037	-1.96	3.27E-12	2.25E-11	down	putative RhoGEF group protein
19886838	BBA_03826	-1.95	2.38E-48	6.78E-47	down	sporulation associated protein
19892906	BBA_09894	-1.94	1.30E-17	1.26E-16	down	hypothetical protein BBA_09894
19888643	BBA_05631	-1.94	1.54E-34	2.93E-33	down	glucosamine-6-phosphate isomerase
19884669	BBA_01657	-1.93	8.67E-47	2.34E-45	down	prolyl oligopeptidase
19892697	BBA_09685	-1.93	3.51E-04	9.39E-04	down	metalloprotease-like protein
19883669	BBA_00657	-1.93	2.75E-24	3.68E-23	down	purine-cytosine permease FCY21
19885394	BBA_02382	-1.92	7.38E-35	1.44E-33	down	Cytochrome P450 CYP52T1
19887605	BBA_04593	-1.92	3.55E-17	3.36E-16	down	hypothetical protein BBA_04593
19885042	BBA_02030	-1.92	1.12E-42	2.77E-41	down	aldehyde dehydrogenase
19885165	BBA_02153	-1.91	5.09E-21	5.92E-20	down	putative endochitinase CHI3
19892472	BBA_09460	-1.91	1.87E-20	2.11E-19	down	SAM dependent carboxyl methyltransferase
19885197	BBA_02185	-1.91	6.32E-24	8.32E-23	down	phosphatidylserine decarboxylase-like protein

19891269	BBA_08257	-1.90	1.13E-05	3.87E-05	down	choline transport protein
19885140	BBA_02128	-1.90	5.62E-08	2.65E-07	down	Glycoside hydrolase, family 31
19892460	BBA_09448	-1.90	4.75E-13	3.50E-12	down	hypothetical protein BBA_09448
19892174	BBA_09162	-1.89	3.27E-32	5.82E-31	down	LipA and NB-ARC domain protein
19891925	BBA_08913	-1.89	2.09E-29	3.36E-28	down	hypothetical protein BBA_08913
19893175	BBA_10163	-1.88	5.90E-25	8.23E-24	down	hypothetical protein BBA_10163
19892930	BBA_09918	-1.88	1.76E-24	2.40E-23	down	protein kinase domain-containing protein
19883787	BBA_00775	-1.88	4.82E-43	1.20E-41	down	glutamate/Leucine/Phenylalanine/Valine dehydrogenase
19887417	BBA_04405	-1.88	8.34E-31	1.42E-29	down	LEA domain protein
19887874	BBA_04862	-1.88	2.31E-30	3.87E-29	down	HET domain protein
19888723	BBA_05711	-1.88	9.03E-33	1.64E-31	down	CFEM domain-containing protein
19891777	BBA_08765	-1.88	4.47E-05	1.39E-04	down	tyrosinase-like protein
19883244	BBA_00232	-1.87	7.49E-16	6.60E-15	down	hypothetical protein BBA_00232
19884113	BBA_01101	-1.87	8.17E-11	5.09E-10	down	C6 zinc finger domain-containing protein
19889365	BBA_06353	-1.87	4.01E-12	2.75E-11	down	Cytochrome P450 CYP660A2
19887113	BBA_04101	-1.87	1.26E-22	1.56E-21	down	3'5'-cyclic nucleotide phosphodiesterase
19886150	BBA_03138	-1.87	5.31E-16	4.71E-15	down	LysM domain-containing protein
19892936	BBA_09924	-1.86	3.53E-11	2.27E-10	down	zinc finger protein
19893292	BBA_10280	-1.86	3.20E-14	2.56E-13	down	DnaJ domain containing protein
19888732	BBA_05720	-1.86	1.63E-14	1.34E-13	down	
19888591	BBA_05579	-1.86	3.56E-40	8.20E-39	down	UPF0311 protein
19889234	BBA_06222	-1.86	1.41E-02	2.69E-02	down	protealysin-like protein
19889647	BBA_06635	-1.86	2.19E-06	8.40E-06	down	hypothetical protein BBA_06635
19888372	BBA_05360	-1.86	8.26E-38	1.76E-36	down	tyrosine phosphatase
19883582	BBA_00570	-1.86	1.39E-16	1.28E-15	down	retrograde regulation protein
19889217	BBA_06205	-1.85	4.99E-36	1.01E-34	down	ankyrin repeat protein
19885719	BBA_02707	-1.85	1.34E-06	5.32E-06	down	hypothetical protein BBA_02707
19883357	BBA_00345	-1.85	5.96E-14	4.66E-13	down	iron-sulfur cluster-binding protein
19893287	BBA_10275	-1.85	2.08E-06	7.99E-06	down	hypothetical protein BBA_10275
19889931	BBA_06919	-1.84	7.86E-37	1.62E-35	down	SH3 domain-containing protein
19893181	BBA_10169	-1.84	1.37E-22	1.70E-21	down	ATPase protein
19888609	BBA_05597	-1.83	5.05E-18	4.99E-17	down	RBP protein
19886582	BBA_03570	-1.83	1.04E-17	1.01E-16	down	hypothetical protein BBA_03570
19893239	BBA_10227	-1.83	2.20E-05	7.17E-05	down	hypothetical protein BBA_10227
19891476	BBA_08464	-1.83	4.86E-08	2.31E-07	down	serine proteinase-like protein
19885194	BBA_02182	-1.83	9.54E-42	2.29E-40	down	cholinesterase-like protein
19891756	BBA_08744	-1.83	7.83E-29	1.23E-27	down	aspartokinase-like protein
19885420	BBA_02408	-1.83	3.31E-21	3.89E-20	down	Ankyrin repeat protein
19885819	BBA_02807	-1.82	1.11E-40	2.58E-39	down	hypothetical protein BBA_02807
19886450	BBA_03438	-1.82	2.16E-36	4.43E-35	down	Acyl-CoA N-acyltransferase
19891306	BBA_08294	-1.82	1.27E-27	1.93E-26	down	hypothetical protein BBA_08294
19885667	BBA_02655	-1.82	1.19E-12	8.49E-12	down	carbon-nitrogen hydrolase
19889392	BBA_06380	-1.82	2.04E-25	2.89E-24	down	glucanase B
19892693	BBA_09681	-1.82	1.58E-38	3.44E-37	down	sarcosine oxidase
19891821	BBA_08809	-1.81	3.53E-29	5.63E-28	down	carboxypeptidase Y
19883780	BBA_00768	-1.81	1.93E-37	4.09E-36	down	major facilitator superfamily transporter
19887253	BBA_04241	-1.80	2.93E-32	5.22E-31	down	phosphoesterase-like protein
19884827	BBA_01815	-1.80	2.55E-12	1.77E-11	down	hypothetical protein BBA_01815
19885948	BBA_02936	-1.79	1.80E-17	1.73E-16	down	peptidase family protein
19886213	BBA_03201	-1.79	9.78E-14	7.57E-13	down	aminotransferase class-V
19885954	BBA_02942	-1.79	3.21E-11	2.07E-10	down	LysM domain containing protein
19888259	BBA_05247	-1.79	6.43E-26	9.22E-25	down	hypothetical protein BBA_05247
19883946	BBA_00934	-1.79	2.06E-24	2.78E-23	down	sterigmatocystin 8-O-methyltransferase
19888252	BBA_05240	-1.79	2.40E-39	5.43E-38	down	BolA-like protein
19885797	BBA_02785	-1.79	3.94E-38	8.47E-37	down	hypothetical protein BBA_02785
19891910	BBA_08898	-1.78	1.62E-31	2.81E-30	down	Peptidase cysteine/serine, trypsin
19889924	BBA_06912	-1.78	5.12E-16	4.54E-15	down	hypothetical protein BBA_06912
19884438	BBA_01426	-1.78	3.21E-34	6.03E-33	down	piwi domain-containing protein
19887540	BBA_04528	-1.77	1.21E-04	3.51E-04	down	geranylgeranyl pyrophosphate synthetase
19888235	BBA_05223	-1.77	1.29E-18	1.32E-17	down	fungal specific transcription factor
19888764	BBA_05752	-1.77	4.80E-32	8.48E-31	down	hypothetical protein BBA_05752

19889035	BBA_06023	-1.77	2.57E-10	1.53E-09	down	hypothetical protein BBA_06023
19885171	BBA_02159	-1.77	9.79E-30	1.59E-28	down	homoserine O-acetyltransferase
19891535	BBA_08523	-1.77	5.38E-14	4.21E-13	down	fungal specific transcription factor domain protein
19892847	BBA_09835	-1.76	4.10E-14	3.25E-13	down	hypothetical protein BBA_09835
19884667	BBA_01655	-1.76	2.62E-36	5.36E-35	down	sulfate permease
19884739	BBA_01727	-1.75	3.38E-06	1.26E-05	down	hypothetical protein BBA_01727
19890996	BBA_07984	-1.75	8.31E-25	1.15E-23	down	hypothetical protein BBA_07984
19890148	BBA_07136	-1.75	2.41E-06	9.20E-06	down	major facilitator superfamily transporter
19890798	BBA_07786	-1.75	1.05E-34	2.02E-33	down	calcium-translocating P-type ATPase
19884668	BBA_01656	-1.74	1.42E-08	7.22E-08	down	amine oxidase
19886456	BBA_03444	-1.74	1.82E-34	3.46E-33	down	POT family protein
19884540	BBA_01528	-1.74	8.49E-27	1.26E-25	down	frequency clock protein
19888853	BBA_05841	-1.74	1.56E-35	3.09E-34	down	ABC transporter, putative
19885579	BBA_02567	-1.74	3.27E-38	7.05E-37	down	nitrogen assimilation transcription factor nirA
19884822	BBA_01810	-1.73	1.15E-23	1.49E-22	down	nonribosomal peptide synthase
19890472	BBA_07460	-1.73	3.11E-14	2.50E-13	down	hypothetical protein BBA_07460
19890729	BBA_07717	-1.73	1.02E-35	2.04E-34	down	hypothetical protein BBA_07717
19884436	BBA_01424	-1.73	7.65E-20	8.36E-19	down	hypothetical protein BBA_01424
19885888	BBA_02876	-1.73	2.63E-31	4.55E-30	down	vivid PAS protein VVD
19884539	BBA_01527	-1.72	9.86E-30	1.60E-28	down	hypothetical protein BBA_01527
19889065	BBA_06053	-1.72	1.47E-29	2.37E-28	down	short-chain dehydrogenase/reductase
19883553	BBA_00541	-1.72	2.49E-23	3.20E-22	down	phosphatidylserine decarboxylase family protein
19884513	BBA_01501	-1.72	4.09E-20	4.54E-19	down	major facilitator superfamily transporter
19888659	BBA_05647	-1.72	1.15E-22	1.44E-21	down	NAD/FAD-binding protein
19888386	BBA_05374	-1.72	3.34E-23	4.26E-22	down	hypothetical protein BBA_05374
19890473	BBA_07461	-1.72	8.74E-14	6.77E-13	down	Peptidase S8/S53, subtilisin/kexin/sedolisin
19889226	BBA_06214	-1.72	3.25E-09	1.77E-08	down	Cytochrome P450 CYP5293A1
19891043	BBA_08031	-1.71	5.53E-27	8.25E-26	down	hypothetical protein BBA_08031
19892140	BBA_09128	-1.71	3.02E-25	4.24E-24	down	Concanavalin A-like lectin/glucanase
19885738	BBA_02726	-1.71	1.96E-04	5.48E-04	down	hypothetical protein BBA_02726
19884679	BBA_01667	-1.71	4.78E-07	2.01E-06	down	forkhead domain-containing protein
19893252	BBA_10240	-1.70	1.26E-29	2.03E-28	down	histidine acid phosphatase
19887242	BBA_04230	-1.70	5.04E-33	9.20E-32	down	oxidoreductase family protein
19892630	BBA_09618	-1.70	1.22E-34	2.34E-33	down	dynamin GTPase, putative
19889003	BBA_05991	-1.70	7.99E-36	1.60E-34	down	PH domain-containing protein
19884405	BBA_01393	-1.70	6.60E-07	2.73E-06	down	hypothetical protein BBA_01393
19888647	BBA_05635	-1.69	4.35E-19	4.56E-18	down	nitrite reductase
19890881	BBA_07869	-1.69	9.91E-23	1.24E-21	down	nudix domain containing protein
19892135	BBA_09123	-1.69	1.38E-10	8.40E-10	down	multidrug resistance protein MDR, putative
19892194	BBA_09182	-1.69	1.18E-32	2.14E-31	down	Glycoside hydrolase, family 36
19883837	BBA_00825	-1.69	5.16E-37	1.07E-35	down	Major Facilitator Superfamily protein
19888637	BBA_05625	-1.69	1.43E-19	1.54E-18	down	metal ion transporter metal ion transporter
19885837	BBA_02825	-1.69	1.25E-10	7.65E-10	down	proline transporter
19884142	BBA_01130	-1.69	2.21E-07	9.67E-07	down	hypothetical protein BBA_01130
19893180	BBA_10168	-1.69	2.28E-15	1.96E-14	down	geranylgeranyl pyrophosphate synthetase
19892849	BBA_09837	-1.68	1.97E-05	6.49E-05	down	TPR repeat protein
19884076	BBA_01064	-1.68	3.46E-07	1.48E-06	down	hypothetical protein BBA_01064
19885385	BBA_02373	-1.68	4.65E-09	2.49E-08	down	major facilitator superfamily transporter
19885517	BBA_02505	-1.68	2.66E-26	3.89E-25	down	homeobox domain-containing protein
19887803	BBA_04791	-1.68	1.33E-12	9.44E-12	down	hypothetical protein BBA_04791
19893368	BBA_10356	-1.68	1.60E-16	1.47E-15	down	reverse transcriptase
19890942	BBA_07930	-1.68	1.13E-19	1.22E-18	down	protein kinase subdomain-containing protein
19893088	BBA_10076	-1.67	2.22E-12	1.55E-11	down	CAMK family protein kinase
19893071	BBA_10059	-1.67	1.20E-17	1.17E-16	down	p63 related protein
19890091	BBA_07079	-1.67	1.91E-33	3.53E-32	down	hypothetical protein BBA_07079
19887282	BBA_04270	-1.67	1.57E-10	9.54E-10	down	hypothetical protein BBA_04270
19884824	BBA_01812	-1.67	2.58E-33	4.76E-32	down	HD domain-containing protein
19888986	BBA_05974	-1.66	9.98E-09	5.13E-08	down	hypothetical protein BBA_05974
19885559	BBA_02547	-1.66	5.66E-31	9.64E-30	down	hypothetical protein BBA_02547
19886852	BBA_03840	-1.66	6.71E-35	1.31E-33	down	hypothetical protein BBA_03840
19890966	BBA_07954	-1.66	7.59E-25	1.05E-23	down	ammonium permease MepC

19891856	BBA_08844	-1.66	3.02E-03	6.70E-03	down	eukaryotic aspartyl protease
19887159	BBA_04147	-1.65	1.84E-16	1.68E-15	down	F-box domain-containing protein
19890120	BBA_07108	-1.65	1.79E-09	9.91E-09	down	hypothetical protein BBA_07108
19883615	BBA_00603	-1.65	4.05E-18	4.03E-17	down	AMP-binding enzyme
19892907	BBA_09895	-1.65	2.51E-24	3.38E-23	down	hypothetical protein BBA_09895
19893064	BBA_10052	-1.64	2.80E-06	1.06E-05	down	hypothetical protein BBA_10052
19890554	BBA_07542	-1.63	2.41E-21	2.83E-20	down	hypothetical protein BBA_07542
19884602	BBA_01590	-1.63	2.21E-14	1.80E-13	down	
19885108	BBA_02096	-1.63	3.24E-31	5.59E-30	down	hypothetical protein BBA_02096
19889164	BBA_06152	-1.63	3.25E-28	5.06E-27	down	RTA1 like protein
19887672	BBA_04660	-1.63	1.13E-16	1.04E-15	down	hypothetical protein BBA_04660
19889279	BBA_06267	-1.62	1.08E-10	6.66E-10	down	flavin-binding monooxygenase, putative
19892175	BBA_09163	-1.62	7.97E-33	1.45E-31	down	carbohydrate-binding module family 21
19885218	BBA_02206	-1.62	1.32E-33	2.45E-32	down	carboxypeptidase S1
19889439	BBA_06427	-1.62	4.13E-30	6.82E-29	down	glycoside hydrolase family 13
19885241	BBA_02229	-1.61	9.14E-33	1.66E-31	down	hypothetical protein BBA_02229
19884141	BBA_01129	-1.61	3.97E-32	7.03E-31	down	inversin protein alternative isoform
19884161	BBA_01149	-1.61	2.23E-22	2.74E-21	down	alpha-1,3-mannosyltransferase CMT1
19891931	BBA_08919	-1.61	1.59E-28	2.49E-27	down	NRPS-like enzyme
19891725	BBA_08713	-1.61	1.28E-09	7.21E-09	down	hypothetical protein BBA_08713
19889544	BBA_06532	-1.60	2.88E-10	1.71E-09	down	DUF500 domain protein
19889094	BBA_06082	-1.60	3.36E-30	5.58E-29	down	ATP synthase F0
19892846	BBA_09834	-1.60	2.53E-05	8.17E-05	down	hypothetical protein BBA_09834
19883251	BBA_00239	-1.60	1.21E-14	1.00E-13	down	transcription factor Domain protein family
19885329	BBA_02317	-1.60	7.79E-21	8.98E-20	down	serine/threonine-protein kinase nak1
19883396	BBA_00384	-1.60	8.04E-17	7.48E-16	down	hypothetical protein BBA_00384
19884823	BBA_01811	-1.60	7.36E-18	7.25E-17	down	NAD dependent epimerase/dehydratase
19886083	BBA_03071	-1.60	9.58E-04	2.36E-03	down	hydrophobin-like protein
19887940	BBA_04928	-1.60	1.48E-10	8.99E-10	down	hypothetical protein BBA_04928
19884676	BBA_01664	-1.60	1.05E-29	1.71E-28	down	DNA photolyase
19884922	BBA_01910	-1.59	6.50E-24	8.54E-23	down	hypothetical protein BBA_01910
19885338	BBA_02326	-1.59	2.32E-09	1.28E-08	down	sodium symporter family protein
19888641	BBA_05629	-1.58	1.00E-28	1.57E-27	down	carbohydrate esterase family 9
19888378	BBA_05366	-1.58	4.81E-06	1.75E-05	down	hypothetical protein BBA_05366
19889351	BBA_06339	-1.58	9.21E-19	9.48E-18	down	major facilitator superfamily transporter
19885240	BBA_02228	-1.58	4.90E-20	5.40E-19	down	hypothetical protein BBA_02228
19892134	BBA_09122	-1.58	6.15E-05	1.87E-04	down	hypothetical protein BBA_09122
19887074	BBA_04062	-1.58	2.02E-30	3.39E-29	down	FAD binding domain-containing protein
19891540	BBA_08528	-1.57	3.48E-08	1.68E-07	down	hypothetical protein BBA_08528
19887352	BBA_04340	-1.57	4.08E-10	2.39E-09	down	hypothetical protein BBA_04340
19883317	BBA_00305	-1.57	3.54E-26	5.14E-25	down	Cytochrome P450 CYP628A2
19883107	BBA_00095	-1.57	2.23E-29	3.57E-28	down	
19884131	BBA_01119	-1.57	1.15E-09	6.49E-09	down	Di-trans-poly-cis-decaprenylcistransferase-like protein
19889661	BBA_06649	-1.57	4.51E-27	6.75E-26	down	hypothetical protein BBA_06649
19886689	BBA_03677	-1.57	3.44E-12	2.37E-11	down	hypothetical protein BBA_03677
19886101	BBA_03089	-1.56	3.45E-07	1.48E-06	down	tetratricopeptide repeat domain protein
19890903	BBA_07891	-1.56	9.61E-22	1.15E-20	down	bZIP transcription factor
19891640	BBA_08628	-1.56	7.78E-11	4.87E-10	down	Hsp70 family chaperone
19892348	BBA_09336	-1.56	1.02E-07	4.65E-07	down	MFS quinate transporter, putative
19892293	BBA_09281	-1.56	9.79E-10	5.56E-09	down	hypothetical protein BBA_09281
19885734	BBA_02722	-1.55	1.18E-21	1.40E-20	down	nitrate assimilation regulatory protein nirA
19891095	BBA_08083	-1.55	3.76E-13	2.79E-12	down	helix-loop-helix DNA-binding domain-containing protein
19883578	BBA_00566	-1.55	2.08E-03	4.75E-03	down	hypothetical protein BBA_00566
19890624	BBA_07612	-1.55	2.96E-30	4.94E-29	down	FAD binding domain-containing protein
19891044	BBA_08032	-1.55	2.91E-07	1.25E-06	down	phosphotransferase enzyme family protein
19886243	BBA_03231	-1.55	1.08E-13	8.32E-13	down	chorismate synthase
19887955	BBA_04943	-1.55	3.18E-05	1.01E-04	down	putative carotenoid oxygenase
19887568	BBA_04556	-1.55	1.98E-18	2.01E-17	down	RTA1 like protein
19892044	BBA_09032	-1.55	2.16E-05	7.07E-05	down	hypothetical protein BBA_09032
19883611	BBA_00599	-1.54	1.82E-19	1.96E-18	down	
19889449	BBA_06437	-1.54	5.98E-25	8.32E-24	down	Glycoside hydrolase, catalytic core

19889838	BBA_06826	-1.54	4.80E-08	2.28E-07	down	glucose oxidase precursor
19885668	BBA_02656	-1.54	1.24E-24	1.69E-23	down	LPXTG-domain-containing protein
19892268	BBA_09256	-1.54	4.36E-14	3.44E-13	down	prephenate dehydratase
19885596	BBA_02584	-1.54	1.68E-30	2.83E-29	down	oxidoreductase family protein
19884143	BBA_01131	-1.54	3.74E-20	4.15E-19	down	hypothetical protein BBA_01131
19885553	BBA_02541	-1.54	3.22E-26	4.70E-25	down	dihydroxyacetone kinase
19885373	BBA_02361	-1.54	7.83E-22	9.37E-21	down	FMN-binding split barrel-related protein
19893184	BBA_10172	-1.53	9.50E-09	4.90E-08	down	hypothetical protein BBA_10172
19883598	BBA_00586	-1.53	5.48E-27	8.19E-26	down	DNA polymerase POL4, putative
19890386	BBA_07374	-1.53	9.13E-08	4.19E-07	down	hypothetical protein BBA_07374
19887334	BBA_04322	-1.53	2.74E-20	3.06E-19	down	transcription factor
19883525	BBA_00513	-1.53	2.69E-08	1.32E-07	down	hypothetical protein BBA_00513
19887876	BBA_04864	-1.52	8.77E-28	1.35E-26	down	WD domain-containing protein
19892563	BBA_09551	-1.52	8.78E-13	6.33E-12	down	C6 transcription factor, putative
19883768	BBA_00756	-1.52	4.34E-22	5.26E-21	down	cAMP-independent regulatory protein pac2
19887097	BBA_04085	-1.52	5.79E-07	2.41E-06	down	hypothetical protein BBA_04085
19886840	BBA_03828	-1.52	5.53E-05	1.70E-04	down	Cytochrome P450 CYP540B16
19884760	BBA_01748	-1.52	2.71E-23	3.47E-22	down	NADPH dehydrogenase
19887952	BBA_04940	-1.52	4.65E-05	1.45E-04	down	RNase H domain containing protein
19883087	BBA_00075	-1.52	5.05E-10	2.94E-09	down	hypothetical protein BBA_00075
19893068	BBA_10056	-1.52	6.91E-27	1.02E-25	down	Amino acid/polyamine transporter I
19884193	BBA_01181	-1.52	1.34E-06	5.34E-06	down	heterokaryon incompatibility protein
19888089	BBA_05077	-1.51	7.17E-12	4.85E-11	down	hypothetical protein BBA_05077
19892845	BBA_09833	-1.51	9.39E-06	3.28E-05	down	hypothetical protein BBA_09833
19889873	BBA_06861	-1.51	8.31E-09	4.32E-08	down	inner membrane transport protein yfaV
19891662	BBA_08650	-1.51	6.50E-20	7.11E-19	down	hypothetical protein BBA_08650
19885711	BBA_02699	-1.51	6.11E-17	5.71E-16	down	mitochondrial chaperone BCS1
19889735	BBA_06723	-1.51	2.50E-12	1.74E-11	down	hypothetical protein BBA_06723
19887082	BBA_04070	-1.50	5.99E-13	4.38E-12	down	calcium/calmodulin dependent protein kinase C, putative
19892223	BBA_09211	-1.50	2.01E-05	6.60E-05	down	exo-beta-1,3-glucanase, putative
19892217	BBA_09205	-1.50	1.50E-12	1.06E-11	down	reverse transcriptase
19884015	BBA_01003	-1.49	1.82E-12	1.28E-11	down	subtilase-like protein
19889087	BBA_06075	-1.49	7.90E-18	7.76E-17	down	hypothetical protein BBA_06075
19893364	BBA_10352	-1.49	2.50E-14	2.02E-13	down	protein kinase domain-containing protein
19890339	BBA_07327	-1.49	2.86E-14	2.30E-13	down	putative MEI5 protein
19885628	BBA_02616	-1.49	2.74E-08	1.34E-07	down	receptor-interacting serine/threonine-protein kinase
19892887	BBA_09875	-1.49	4.96E-03	1.05E-02	down	O-methyltransferase, family 3
19890941	BBA_07929	-1.49	2.19E-06	8.40E-06	down	nucleosome binding protein
19883455	BBA_00443	-1.48	7.52E-16	6.63E-15	down	cuticle-degrading protease bassiasin I precursor
19891636	BBA_08624	-1.48	2.79E-20	3.12E-19	down	ATP synthase F0
19892066	BBA_09054	-1.48	1.77E-23	2.29E-22	down	putative MEI5 protein
19888285	BBA_05273	-1.48	8.77E-08	4.04E-07	down	hypothetical protein BBA_05273
19892131	BBA_09119	-1.48	1.03E-26	1.52E-25	down	phosphoesterase-like protein
19886490	BBA_03478	-1.47	3.76E-10	2.22E-09	down	DUF829 domain protein (PaxU)
19885921	BBA_02909	-1.47	5.51E-12	3.75E-11	down	kelch repeat protein
19887099	BBA_04087	-1.47	1.17E-21	1.39E-20	down	F5/8 type C domain protein
19892721	BBA_09709	-1.47	2.28E-15	1.96E-14	down	Hsp70 family chaperone, putative
19892841	BBA_09829	-1.47	1.58E-04	4.48E-04	down	hypothetical protein BBA_09829
19890369	BBA_07357	-1.47	3.21E-16	2.87E-15	down	TPR domain protein
19884871	BBA_01859	-1.46	4.43E-06	1.62E-05	down	hypothetical protein BBA_01859
19892688	BBA_09676	-1.46	4.29E-07	1.82E-06	down	MFS allantoate transporter
19886833	BBA_03821	-1.46	1.67E-13	1.27E-12	down	N,N-dimethylglycine oxidase
19890673	BBA_07661	-1.46	1.09E-24	1.49E-23	down	potassium/sodium efflux P-type ATPase
19889518	BBA_06506	-1.46	9.20E-21	1.05E-19	down	carbohydrate esterase family 3
19890492	BBA_07480	-1.46	4.59E-24	6.06E-23	down	membrane copper amine oxidase
19883956	BBA_00944	-1.46	2.28E-12	1.59E-11	down	nitrite transporter
19890721	BBA_07709	-1.46	3.16E-14	2.53E-13	down	transcription factor RfeG
19883237	BBA_00225	-1.46	2.67E-06	1.01E-05	down	hypothetical protein BBA_00225
19887991	BBA_04979	-1.46	1.37E-09	7.71E-09	down	hypothetical protein BBA_04979
19887671	BBA_04659	-1.45	3.45E-17	3.26E-16	down	monooxygenase-like protein
19892548	BBA_09536	-1.45	2.57E-25	3.62E-24	down	transposase-like protein

19889070	BBA_06058	-1.45	1.67E-19	1.79E-18	down	esterase-like protein
19885412	BBA_02400	-1.45	3.38E-26	4.92E-25	down	amidase-like protein
19888194	BBA_05182	-1.45	1.64E-17	1.59E-16	down	ATPase protein
19887342	BBA_04330	-1.45	2.49E-22	3.06E-21	down	BZIP-type transcription factor
19887939	BBA_04927	-1.45	1.42E-05	4.79E-05	down	arylesterase/monooxygenase
19885980	BBA_02968	-1.44	1.65E-11	1.09E-10	down	regulator of G protein signaling
19884409	BBA_01397	-1.44	3.26E-04	8.75E-04	down	ABC multidrug transporter, putative
19885597	BBA_02585	-1.44	2.59E-08	1.27E-07	down	dihydrodipicolinate synthase
19884665	BBA_01653	-1.44	3.35E-22	4.09E-21	down	DUF1275 domain protein
19888061	BBA_05049	-1.44	5.54E-22	6.68E-21	down	hypothetical protein BBA_05049
19884666	BBA_01654	-1.44	2.07E-24	2.80E-23	down	oxalate decarboxylase family bicupin
19886750	BBA_03738	-1.44	6.65E-13	4.85E-12	down	heterotrimeric G protein beta subunit
19890390	BBA_07378	-1.44	4.41E-24	5.85E-23	down	putative histidine kinase HHK3p
19883195	BBA_00183	-1.44	5.58E-08	2.63E-07	down	major facilitator superfamily transporter
19891312	BBA_08300	-1.44	9.36E-14	7.25E-13	down	arylamine N-acetyltransferase 1
19890318	BBA_07306	-1.44	2.58E-13	1.94E-12	down	hypothetical protein BBA_07306
19890680	BBA_07668	-1.44	6.74E-11	4.25E-10	down	peptidase C14
19890434	BBA_07422	-1.43	1.89E-11	1.25E-10	down	hypothetical protein BBA_07422
19890784	BBA_07772	-1.43	5.51E-09	2.92E-08	down	reverse transcriptase
19886880	BBA_03868	-1.43	1.94E-24	2.63E-23	down	piwi domain-containing protein
19887835	BBA_04823	-1.43	2.69E-17	2.56E-16	down	hypothetical protein BBA_04823
19893023	BBA_10011	-1.42	5.46E-19	5.69E-18	down	DUF323 domain-containing protein
19888179	BBA_05167	-1.42	5.89E-06	2.11E-05	down	peptidase family M48
19888600	BBA_05588	-1.42	2.69E-19	2.85E-18	down	MAP kinase kinase skh1/pek1
19890701	BBA_07689	-1.42	9.48E-07	3.85E-06	down	transposase-like protein
19887878	BBA_04866	-1.42	2.85E-13	2.13E-12	down	finger protein AZF1
19892931	BBA_09919	-1.42	1.41E-04	4.04E-04	down	hypothetical protein BBA_09919
19889396	BBA_06384	-1.41	8.53E-07	3.47E-06	down	hypothetical protein BBA_06384
19884404	BBA_01392	-1.41	3.89E-18	3.88E-17	down	glycosyltransferase family 2
19892852	BBA_09840	-1.41	1.51E-15	1.31E-14	down	hypothetical protein BBA_09840
19888832	BBA_05820	-1.41	1.33E-15	1.16E-14	down	C6 transcription factor, putative
19891254	BBA_08242	-1.41	3.70E-08	1.78E-07	down	glycoside hydrolase family 43 protein
19891368	BBA_08356	-1.41	3.64E-21	4.27E-20	down	integral membrane protein (Pth11)
19888267	BBA_05255	-1.41	6.61E-07	2.73E-06	down	ankyrin repeat protein
19889909	BBA_06897	-1.40	1.79E-12	1.26E-11	down	hypothetical protein BBA_06897
19892471	BBA_09459	-1.40	1.98E-13	1.50E-12	down	hypothetical protein BBA_09459
19887807	BBA_04795	-1.40	4.01E-12	2.75E-11	down	hypothetical protein BBA_04795
19884242	BBA_01230	-1.39	2.59E-20	2.90E-19	down	ZIP Zinc transporter
19884021	BBA_01009	-1.39	2.59E-11	1.69E-10	down	amino acid permease
19889339	BBA_06327	-1.39	1.73E-03	4.03E-03	down	methyltransferase-like protein
19885193	BBA_02181	-1.38	9.19E-26	1.31E-24	down	ornithine carbamoyltransferase
19884140	BBA_01128	-1.38	9.70E-24	1.27E-22	down	yippee family protein
19890157	BBA_07145	-1.38	1.16E-12	8.27E-12	down	hypothetical protein BBA_07145
19885772	BBA_02760	-1.38	2.94E-23	3.76E-22	down	Beta-lactamase-type transpeptidase
19883331	BBA_00319	-1.38	1.65E-10	1.00E-09	down	Peptidase S8
19887643	BBA_04631	-1.38	1.05E-23	1.37E-22	down	Cytochrome P450 CYP6004A2
19890143	BBA_07131	-1.37	2.08E-06	8.00E-06	down	C3HC4 type (RING finger) zinc finger containing protein
19888134	BBA_05122	-1.37	3.65E-24	4.86E-23	down	hypothetical protein BBA_05122
19893333	BBA_10321	-1.37	3.15E-16	2.82E-15	down	Dynamin family protein
19885759	BBA_02747	-1.37	2.56E-05	8.26E-05	down	Cytochrome P450 CYP682N1
19884861	BBA_01849	-1.37	7.17E-16	6.33E-15	down	hydroxyproline-rich glycoprotein DZ-HRGP
19887970	BBA_04958	-1.37	7.45E-19	7.70E-18	down	RadR putative transcriptional regulator
19883054	BBA_00042	-1.37	2.61E-24	3.51E-23	down	homoserine acetyltransferase family protein
19883304	BBA_00292	-1.37	5.77E-19	5.99E-18	down	WD domain-containing protein
19887772	BBA_04760	-1.36	7.25E-09	3.80E-08	down	Putative Zn(II)2Cys6 transcription factor
19889168	BBA_06156	-1.36	2.30E-21	2.70E-20	down	sulfate transporter
19884402	BBA_01390	-1.36	3.90E-14	3.09E-13	down	hypothetical protein BBA_01390
19892926	BBA_09914	-1.36	1.37E-09	7.73E-09	down	dynamin GTPase
19885007	BBA_01995	-1.36	2.41E-23	3.10E-22	down	methyltransferase-like protein
19892239	BBA_09227	-1.36	7.71E-07	3.15E-06	down	ferric reductase like transmembrane component
19887393	BBA_04381	-1.35	3.15E-22	3.84E-21	down	hypothetical protein BBA_04381

19883409	BBA_00397	-1.35	5.52E-23	6.97E-22	down	hexose transporter
19891226	BBA_08214	-1.35	4.56E-12	3.11E-11	down	cell wall glucanosyltransferase Mwg1
19886908	BBA_03896	-1.35	3.40E-11	2.19E-10	down	dsp1-1-like protein
19883212	BBA_00200	-1.35	9.84E-07	3.98E-06	down	hypothetical protein BBA_00200
19890732	BBA_07720	-1.35	5.32E-11	3.39E-10	down	hypothetical protein BBA_07720
19890704	BBA_07692	-1.35	7.02E-24	9.20E-23	down	dimethylaniline monooxygenase 3
19884028	BBA_01016	-1.35	8.16E-23	1.02E-21	down	Ribonuclease/ribotoxin
19890491	BBA_07479	-1.35	2.00E-21	2.37E-20	down	putative peptidylarginine deiminase
19887382	BBA_04370	-1.34	8.45E-18	8.30E-17	down	Cytochrome P450 CYP584G1
19892220	BBA_09208	-1.34	5.54E-06	2.00E-05	down	dimethylaniline monooxygenase
19892614	BBA_09602	-1.34	1.51E-06	5.95E-06	down	hypothetical protein BBA_09602
19887190	BBA_04178	-1.34	3.61E-13	2.68E-12	down	cyclopropane-fatty-acyl-phospholipid synthase
19884825	BBA_01813	-1.34	1.79E-16	1.64E-15	down	NADP(+) coupled glycerol dehydrogenase
19884348	BBA_01336	-1.33	1.30E-18	1.33E-17	down	bicyclomycin resistance protein
19888866	BBA_05854	-1.33	1.48E-16	1.36E-15	down	putative phospholipase
19886235	BBA_03223	-1.33	5.72E-11	3.63E-10	down	capsular associated protein
19884757	BBA_01745	-1.33	1.97E-05	6.48E-05	down	hypothetical protein BBA_01745
19886697	BBA_03685	-1.33	1.18E-21	1.40E-20	down	tetratricopeptide repeat domain-containing protein
19892910	BBA_09898	-1.33	2.12E-08	1.05E-07	down	FluG domain-containing protein
19883980	BBA_00968	-1.33	5.47E-18	5.41E-17	down	hypothetical protein BBA_00968
19890601	BBA_07589	-1.33	4.73E-10	2.76E-09	down	nonribosomal peptide synthase, putative
19887477	BBA_04465	-1.32	4.65E-04	1.21E-03	down	hypothetical protein BBA_04465
19883411	BBA_00399	-1.32	1.12E-11	7.49E-11	down	guanine nucleotide exchange factor synembryn
19890676	BBA_07664	-1.32	5.06E-14	3.97E-13	down	stearoyl-CoA desaturase
19889082	BBA_06070	-1.32	2.00E-05	6.59E-05	down	ABC multidrug transporter
19892492	BBA_09480	-1.32	9.49E-08	4.35E-07	down	hypothetical protein BBA_09480
19892838	BBA_09826	-1.32	8.63E-05	2.56E-04	down	hypothetical protein BBA_09826
19886416	BBA_03404	-1.32	6.60E-07	2.73E-06	down	MFS multidrug transporter
19888193	BBA_05181	-1.32	3.46E-18	3.46E-17	down	alpha/beta fold family hydrolase
19892115	BBA_09103	-1.32	1.72E-05	5.71E-05	down	glyoxalase-like protein
19887186	BBA_04174	-1.31	1.86E-08	9.27E-08	down	hypothetical protein BBA_04174
19883870	BBA_00858	-1.31	1.20E-20	1.37E-19	down	hypothetical protein BBA_00858
19887346	BBA_04334	-1.31	2.84E-16	2.56E-15	down	hypothetical protein BBA_04334
19891674	BBA_08662	-1.31	5.81E-10	3.37E-09	down	Cytochrome P450 CYP561D2P
19885436	BBA_02424	-1.31	9.71E-15	8.08E-14	down	DASH family cryptochrome
19885226	BBA_02214	-1.30	3.16E-11	2.04E-10	down	subtilase-like protein
19886100	BBA_03088	-1.30	3.91E-11	2.50E-10	down	Ankyrin repeat protein
19890782	BBA_07770	-1.30	1.64E-03	3.84E-03	down	hypothetical protein BBA_07770
19889971	BBA_06959	-1.30	3.27E-06	1.23E-05	down	canalicular multispecific organic anion transporter 1
19883513	BBA_00501	-1.30	2.09E-22	2.57E-21	down	negative acting factor
19889551	BBA_06539	-1.30	1.12E-11	7.48E-11	down	TfdA family Taurine catabolism dioxygenase TauD
19885818	BBA_02806	-1.30	7.32E-22	8.81E-21	down	splicing factor Spf30
19888789	BBA_05777	-1.30	1.85E-11	1.22E-10	down	putative HRQ family protein 2
19890413	BBA_07401	-1.29	1.28E-10	7.79E-10	down	fungal specific transcription factor
19883486	BBA_00474	-1.29	1.06E-08	5.42E-08	down	
19890083	BBA_07071	-1.29	1.43E-15	1.24E-14	down	C6 zinc finger domain-containing protein
19892424	BBA_09412	-1.29	1.36E-19	1.47E-18	down	trascription factor
19888079	BBA_05067	-1.29	1.55E-13	1.18E-12	down	RNase3 domain-containing protein
19889208	BBA_06196	-1.29	1.07E-07	4.87E-07	down	monooxygenase, FAD-binding protein
19892525	BBA_09513	-1.29	5.40E-08	2.55E-07	down	hypothetical protein BBA_09513
19891913	BBA_08901	-1.29	2.43E-15	2.08E-14	down	Peptidase S8/S53, subtilisin/kexin/sedolisin
19886455	BBA_03443	-1.29	4.29E-06	1.58E-05	down	ABC transporter
19886286	BBA_03274	-1.29	6.27E-19	6.51E-18	down	hypothetical protein BBA_03274
19884224	BBA_01212	-1.29	1.48E-19	1.59E-18	down	sodium/calcium exchanger protein
19888321	BBA_05309	-1.29	5.47E-06	1.97E-05	down	phosphotransferase enzyme family protein
19889287	BBA_06275	-1.29	3.97E-20	4.41E-19	down	general amino acid permease AGP2
19891090	BBA_08078	-1.29	1.62E-21	1.92E-20	down	hypothetical protein BBA_08078
19885666	BBA_02654	-1.29	1.12E-09	6.33E-09	down	carbon-nitrogen hydrolase
19885242	BBA_02230	-1.29	2.22E-17	2.13E-16	down	chitinase-like protein
19890309	BBA_07297	-1.28	1.12E-13	8.62E-13	down	oxidoreductase, short-chain dehydrogenase/reductase
19889359	BBA_06347	-1.28	4.65E-20	5.13E-19	down	DUF1275 domain protein

19889505	BBA_06493	-1.28	1.83E-05	6.07E-05	down	hypothetical protein BBA_06493
19889606	BBA_06594	-1.28	4.11E-17	3.87E-16	down	Ig domain protein group 2 domain protein
19893069	BBA_10057	-1.28	5.27E-17	4.95E-16	down	Acyl-CoA N-acyltransferase
19886431	BBA_03419	-1.28	4.94E-05	1.53E-04	down	hypothetical protein BBA_03419
19888974	BBA_05962	-1.28	2.40E-17	2.30E-16	down	fungal specific transcription factor, putative
19885444	BBA_02432	-1.28	3.87E-15	3.29E-14	down	triacylglycerol lipase
19885574	BBA_02562	-1.27	7.43E-07	3.05E-06	down	AdoMet-dependent methyltransferase, Putative
19888704	BBA_05692	-1.27	1.98E-14	1.61E-13	down	hypothetical protein BBA_05692
19887836	BBA_04824	-1.27	3.15E-12	2.17E-11	down	ABC transporter with duplicated ATPase domains
19890162	BBA_07150	-1.27	6.41E-18	6.32E-17	down	sedoheptulose-1,7-bisphosphatase
19886779	BBA_03767	-1.27	9.02E-19	9.30E-18	down	uricase-like protein
19887749	BBA_04737	-1.27	1.22E-09	6.90E-09	down	heterokaryon incompatibility protein
19883438	BBA_00426	-1.27	1.37E-17	1.33E-16	down	phospholipase D2
19892617	BBA_09605	-1.27	7.71E-04	1.93E-03	down	chitinase-like protein
19893354	BBA_10342	-1.27	4.23E-08	2.02E-07	down	hypothetical protein BBA_10342
19893282	BBA_10270	-1.27	6.83E-15	5.71E-14	down	restless-like transposase
19889069	BBA_06057	-1.27	6.29E-21	7.27E-20	down	Fungal transcriptional regulatory protein
19883179	BBA_00167	-1.27	4.91E-17	4.61E-16	down	hemolysin-III family protein
19888997	BBA_05985	-1.27	2.59E-20	2.90E-19	down	nitrate assimilation regulatory protein nirA
19890010	BBA_06998	-1.27	5.06E-18	5.01E-17	down	ATROPHIN-1 protein
19884107	BBA_01095	-1.27	3.18E-17	3.02E-16	down	MAP kinase kinase 1
19886475	BBA_03463	-1.27	2.81E-18	2.83E-17	down	acid phosphatase
19887910	BBA_04898	-1.27	2.70E-19	2.86E-18	down	
19885415	BBA_02403	-1.26	5.06E-21	5.89E-20	down	glycosyl hydrolase, putative
19887404	BBA_04392	-1.26	3.05E-14	2.45E-13	down	lipase/thioesterase
19888125	BBA_05113	-1.26	1.51E-04	4.29E-04	down	hypothetical protein BBA_05113
19890037	BBA_07025	-1.26	1.35E-14	1.12E-13	down	ATG1 protein
19885417	BBA_02405	-1.26	4.80E-09	2.57E-08	down	RNA recognition domain-containing protein
19885209	BBA_02197	-1.26	1.79E-19	1.92E-18	down	nucleoside-diphosphate-sugar epimerase
19886610	BBA_03598	-1.26	2.18E-09	1.20E-08	down	RasGEF domain-containing protein
19890956	BBA_07944	-1.26	1.49E-18	1.52E-17	down	PAF acetylhydrolase
19890999	BBA_07987	-1.26	4.63E-14	3.64E-13	down	hypothetical protein BBA_07987
19889919	BBA_06907	-1.26	5.59E-20	6.14E-19	down	histidine acid phosphatase
19890551	BBA_07539	-1.26	1.76E-16	1.61E-15	down	sugar hydrolase
19889294	BBA_06282	-1.26	9.98E-19	1.03E-17	down	fungal specific transcription factor
19890490	BBA_07478	-1.25	2.38E-19	2.53E-18	down	Pfs domain protein
19892459	BBA_09447	-1.25	7.01E-12	4.74E-11	down	hypothetical protein BBA_09447
19893154	BBA_10142	-1.25	1.11E-17	1.08E-16	down	small s protein
19883471	BBA_00459	-1.25	4.61E-20	5.09E-19	down	hypothetical protein BBA_00459
19889432	BBA_06420	-1.25	1.42E-19	1.54E-18	down	oligopeptide transporter
19889315	BBA_06303	-1.25	3.85E-18	3.85E-17	down	methyltransferase domain-containing protein
19892364	BBA_09352	-1.25	3.41E-16	3.05E-15	down	MFS allantoate transporter, putative
19890866	BBA_07854	-1.25	7.04E-06	2.50E-05	down	Pfs, NACHT, and Ankyrin domain protein
19889345	BBA_06333	-1.25	3.72E-06	1.38E-05	down	phospholipase A2
19883088	BBA_00076	-1.25	1.63E-06	6.40E-06	down	thiamin biosynthesis protein (Thi-4)
19891320	BBA_08308	-1.25	4.75E-19	4.98E-18	down	OPT oligopeptide transporter
19892302	BBA_09290	-1.25	5.00E-06	1.82E-05	down	CFEM domain-containing protein
19883724	BBA_00712	-1.25	2.73E-12	1.88E-11	down	putative C6-zinc finger TF, regulator of conidiation
19886796	BBA_03784	-1.25	2.83E-13	2.12E-12	down	alpha/beta hydrolase fold protein
19885572	BBA_02560	-1.24	4.19E-05	1.31E-04	down	dual specificity phosphatase Yvh1, putative
19889117	BBA_06105	-1.24	7.51E-06	2.65E-05	down	hypothetical protein BBA_06105
19886064	BBA_03052	-1.24	9.38E-15	7.81E-14	down	peptidase family M20/M25/M40
19885452	BBA_02440	-1.24	2.33E-13	1.76E-12	down	peptidase M61 domain protein
19883675	BBA_00663	-1.24	3.13E-11	2.03E-10	down	beta-lactamase family protein
19891618	BBA_08606	-1.24	7.95E-11	4.97E-10	down	F-box protein
19888623	BBA_05611	-1.24	1.72E-17	1.65E-16	down	copper-transporting ATPase
19892497	BBA_09485	-1.24	1.36E-14	1.13E-13	down	
19887360	BBA_04348	-1.24	3.69E-14	2.95E-13	down	wd-repeat protein
19884558	BBA_01546	-1.24	2.56E-04	7.02E-04	down	hypothetical protein BBA_01546
19891665	BBA_08653	-1.24	4.51E-06	1.65E-05	down	hypothetical protein BBA_08653
19891515	BBA_08503	-1.24	4.76E-11	3.04E-10	down	hypothetical protein BBA_08503

19888777	BBA_05765	-1.23	7.73E-16	6.81E-15	down	HET domain protein
19888153	BBA_05141	-1.23	1.48E-06	5.85E-06	down	putative NDT80 protein
19892461	BBA_09449	-1.23	1.96E-13	1.49E-12	down	hypothetical protein BBA_09449
19888549	BBA_05537	-1.23	9.84E-16	8.63E-15	down	polysaccharide synthase Cps1p
19887111	BBA_04099	-1.23	3.66E-08	1.77E-07	down	hypothetical protein BBA_04099
19888000	BBA_04988	-1.23	1.95E-19	2.08E-18	down	aminotransferase class I and II
19884326	BBA_01314	-1.22	6.26E-09	3.30E-08	down	peptide transporter MTD1
19884942	BBA_01930	-1.22	3.75E-18	3.75E-17	down	OPT peptide transporter Mtd1
19889530	BBA_06518	-1.22	5.44E-10	3.17E-09	down	DnaJ domain-containing protein
19889574	BBA_06562	-1.22	7.09E-17	6.62E-16	down	hypothetical protein BBA_06562
19889769	BBA_06757	-1.22	4.15E-16	3.70E-15	down	DUF895 domain membrane protein, putative
19889045	BBA_06033	-1.22	5.23E-19	5.46E-18	down	LamB/YcsF family protein
19884081	BBA_01069	-1.22	2.63E-16	2.38E-15	down	fatty-acid amide hydrolase
19890643	BBA_07631	-1.22	7.72E-19	7.98E-18	down	family S53 protease
19886189	BBA_03177	-1.22	1.76E-05	5.84E-05	down	fungal specific transcription factor
19891774	BBA_08762	-1.22	3.05E-14	2.45E-13	down	autophagy related lipase
19886206	BBA_03194	-1.22	4.10E-16	3.66E-15	down	RasGEF domain-containing protein
19890516	BBA_07504	-1.21	3.19E-08	1.55E-07	down	hypothetical protein BBA_07504
19891388	BBA_08376	-1.21	3.40E-19	3.59E-18	down	MAC1 interacting protein
19888838	BBA_05826	-1.21	2.26E-14	1.83E-13	down	20beta-hydroxysteroid dehydrogenase
19887815	BBA_04803	-1.21	1.46E-10	8.84E-10	down	F-box domain-containing protein
19888246	BBA_05234	-1.21	7.59E-14	5.90E-13	down	glycosyl hydrolase
19891632	BBA_08620	-1.21	1.87E-14	1.53E-13	down	drug resistance protein
19890416	BBA_07404	-1.21	6.36E-16	5.63E-15	down	DUF221 family protein
19892465	BBA_09453	-1.21	9.98E-05	2.93E-04	down	hypothetical protein BBA_09453
19885524	BBA_02512	-1.21	2.93E-06	1.11E-05	down	putative ATP-dependent RNA helicase DED1
19884514	BBA_01502	-1.21	8.55E-13	6.18E-12	down	fungal specific transcription factor
19888406	BBA_05394	-1.20	8.85E-15	7.38E-14	down	C4-dicarboxylate transporter/malic acid transporter
19883740	BBA_00728	-1.20	5.74E-19	5.97E-18	down	zinc-binding dehydrogenase
19886832	BBA_03820	-1.20	2.63E-19	2.79E-18	down	sarcosine oxidase
19893261	BBA_10249	-1.20	1.83E-14	1.50E-13	down	kynurenine 3-monooxygenase
19883882	BBA_00870	-1.20	3.20E-04	8.59E-04	down	permease-like protein
19885362	BBA_02350	-1.20	1.49E-09	8.32E-09	down	zinc finger protein 76 (expressed in testis)
19884838	BBA_01826	-1.20	2.96E-12	2.04E-11	down	capsular associated protein
19887810	BBA_04798	-1.20	5.56E-07	2.32E-06	down	hypothetical protein BBA_04798
19890547	BBA_07535	-1.20	2.33E-10	1.39E-09	down	ankyrin repeat protein
19883350	BBA_00338	-1.20	2.81E-17	2.67E-16	down	L-arabinitol 4-dehydrogenase
19891551	BBA_08539	-1.20	3.23E-04	8.69E-04	down	putative SAM-dependent methyltransferase
19889076	BBA_06064	-1.20	2.35E-09	1.29E-08	down	GATA zinc finger protein
19884890	BBA_01878	-1.19	2.62E-14	2.11E-13	down	hypothetical protein BBA_01878
19888758	BBA_05746	-1.19	2.43E-14	1.97E-13	down	FAD binding domain protein
19886976	BBA_03964	-1.19	3.37E-14	2.70E-13	down	Glycoside hydrolase, catalytic core
19892266	BBA_09254	-1.19	1.26E-13	9.68E-13	down	nitrate assimilation regulatory protein nirA
19888798	BBA_05786	-1.19	1.94E-07	8.59E-07	down	protein kinase domain-containing protein
19886578	BBA_03566	-1.19	8.89E-19	9.18E-18	down	transmembrane amino acid transporter
19888748	BBA_05736	-1.19	2.75E-05	8.86E-05	down	MFS monosaccharide transporter (Hxt8)
19887674	BBA_04662	-1.19	1.27E-17	1.23E-16	down	SAM-dependent methyltransferase
19889276	BBA_06264	-1.19	2.59E-09	1.42E-08	down	Brefeldin A-sensitivity protein 4
19888044	BBA_05032	-1.19	5.48E-08	2.59E-07	down	DUF967 domain-containing protein
19891262	BBA_08250	-1.19	2.10E-16	1.91E-15	down	methyltransferase-like protein
19886428	BBA_03416	-1.18	2.39E-08	1.18E-07	down	hypothetical protein BBA_03416
19886626	BBA_03614	-1.18	2.24E-14	1.82E-13	down	hypothetical protein BBA_03614
19888224	BBA_05212	-1.18	7.43E-18	7.31E-17	down	ATP synthase F0
19887037	BBA_04025	-1.18	2.37E-08	1.17E-07	down	glucose oxidase
19884887	BBA_01875	-1.18	2.15E-18	2.18E-17	down	arginase-like protein
19891008	BBA_07996	-1.18	4.11E-18	4.09E-17	down	hypothetical protein BBA_07996
19884367	BBA_01355	-1.18	1.39E-12	9.83E-12	down	hypothetical protein BBA_01355
19889812	BBA_06800	-1.18	1.08E-12	7.73E-12	down	beta-galactosidase
19888897	BBA_05885	-1.18	4.91E-15	4.14E-14	down	FAD binding domain-containing protein
19889122	BBA_06110	-1.18	5.76E-11	3.65E-10	down	palI protein
19885288	BBA_02276	-1.17	8.11E-09	4.22E-08	down	tetratricopeptide repeat domain-containing protein

19883684	BBA_00672	-1.17	7.97E-13	5.77E-12	down	putative glutathione S-transferase
19887980	BBA_04968	-1.17	2.63E-07	1.14E-06	down	hypothetical protein BBA_04968
19888973	BBA_05961	-1.17	1.52E-12	1.08E-11	down	3-hydroxyacid dehydrogenase/reductase
19893024	BBA_10012	-1.16	1.11E-03	2.71E-03	down	FluG domain-containing protein
19888398	BBA_05386	-1.16	3.96E-13	2.93E-12	down	major facilitator superfamily transporter
19883581	BBA_00569	-1.16	3.82E-11	2.45E-10	down	hypothetical protein BBA_00569
19892805	BBA_09793	-1.16	1.47E-07	6.63E-07	down	hypothetical protein BBA_09793
19887920	BBA_04908	-1.16	4.11E-05	1.29E-04	down	penicillin-binding protein
19892358	BBA_09346	-1.16	1.80E-07	7.99E-07	down	amidase-like protein
19884227	BBA_01215	-1.16	1.04E-05	3.61E-05	down	
19887972	BBA_04960	-1.15	2.77E-04	7.57E-04	down	Methyltransferase type 11
19885239	BBA_02227	-1.15	6.93E-13	5.05E-12	down	hypothetical protein BBA_02227
19886012	BBA_03000	-1.15	1.66E-04	4.68E-04	down	isochorismatase-like protein
19886935	BBA_03923	-1.15	6.13E-12	4.16E-11	down	galactonate dehydratase
19890001	BBA_06989	-1.15	8.67E-04	2.16E-03	down	hypothetical protein BBA_06989
19892166	BBA_09154	-1.15	5.43E-14	4.25E-13	down	HET-s/LopB domain-containing protein
19886628	BBA_03616	-1.15	6.74E-10	3.88E-09	down	polyketide synthase, putative
19888719	BBA_05707	-1.15	7.39E-08	3.43E-07	down	UPF0665 family protein c
19892258	BBA_09246	-1.15	4.30E-12	2.94E-11	down	hypothetical protein BBA_09246
19890949	BBA_07937	-1.15	1.10E-10	6.77E-10	down	amino acid kinase
19888720	BBA_05708	-1.15	4.14E-06	1.53E-05	down	putative carbohydrate-binding protein
19890763	BBA_07751	-1.15	2.51E-12	1.74E-11	down	hypothetical protein BBA_07751
19888605	BBA_05593	-1.15	6.02E-16	5.33E-15	down	small s protein
19891975	BBA_08963	-1.15	1.83E-05	6.05E-05	down	hypothetical protein BBA_08963
19884879	BBA_01867	-1.14	1.49E-08	7.53E-08	down	WD domain-containing protein
19889573	BBA_06561	-1.14	1.09E-13	8.42E-13	down	histone-lysine N-methyltransferase
19888310	BBA_05298	-1.14	6.29E-08	2.94E-07	down	DNA repair and transcription factor Ada, putative
19893329	BBA_10317	-1.14	1.32E-11	8.80E-11	down	Ankyrin repeat protein
19883575	BBA_00563	-1.14	8.28E-07	3.38E-06	down	hypothetical protein BBA_00563
19884453	BBA_01441	-1.14	2.35E-08	1.16E-07	down	zinc finger protein SFP1
19885660	BBA_02648	-1.14	6.57E-07	2.72E-06	down	malic enzyme
19891996	BBA_08984	-1.14	1.94E-11	1.27E-10	down	ankyrin repeat-containing protein
19883802	BBA_00790	-1.14	5.74E-15	4.82E-14	down	L-serine dehydratase
19893348	BBA_10336	-1.14	2.04E-11	1.34E-10	down	hypothetical protein BBA_10336
19888464	BBA_05452	-1.13	2.68E-08	1.32E-07	down	T-complex protein 11
19883308	BBA_00296	-1.13	1.15E-10	7.07E-10	down	RING-8 protein
19889935	BBA_06923	-1.13	2.06E-07	9.10E-07	down	YhhN family protein
19892334	BBA_09322	-1.13	3.88E-05	1.23E-04	down	chitin synthase
19883904	BBA_00892	-1.13	5.89E-08	2.76E-07	down	hypothetical protein BBA_00892
19890642	BBA_07630	-1.13	7.36E-04	1.85E-03	down	Rieske domain-containing protein
19889492	BBA_06480	-1.13	2.17E-11	1.42E-10	down	oxidoreductase FAD-binding domain-containing protein
19883907	BBA_00895	-1.13	1.46E-07	6.56E-07	down	RasGEF domain-containing protein
19885470	BBA_02458	-1.13	1.17E-15	1.02E-14	down	hypothetical protein BBA_02458
19886026	BBA_03014	-1.13	2.32E-10	1.39E-09	down	bZIP transcription factor
19888635	BBA_05623	-1.13	2.68E-06	1.02E-05	down	zinc finger protein
19883117	BBA_00105	-1.13	2.15E-03	4.91E-03	down	Putative Zn(II)2Cys6 transcription factor
19885080	BBA_02068	-1.12	1.84E-14	1.51E-13	down	calcineurin-like phosphoesterase
19883467	BBA_00455	-1.12	1.64E-12	1.16E-11	down	MYND domain protein, putative
19885607	BBA_02595	-1.12	3.13E-08	1.52E-07	down	major facilitator superfamily transporter
19890911	BBA_07899	-1.12	3.27E-16	2.93E-15	down	chitinase 18-3
19893089	BBA_10077	-1.12	1.81E-04	5.09E-04	down	Dynamin family protein
19888050	BBA_05038	-1.12	2.75E-04	7.51E-04	down	hypothetical protein BBA_05038
19891745	BBA_08733	-1.12	2.81E-07	1.21E-06	down	allantoicase-like protein
19885827	BBA_02815	-1.12	2.80E-09	1.53E-08	down	ABC transporter
19885159	BBA_02147	-1.12	7.42E-14	5.78E-13	down	radical SAM superfamily protein
19889890	BBA_06878	-1.12	9.23E-15	7.69E-14	down	DNA repair protein (Rad57), putative
19888154	BBA_05142	-1.12	2.10E-09	1.16E-08	down	tafazzin-like protein
19891984	BBA_08972	-1.11	1.70E-06	6.64E-06	down	hypothetical protein BBA_08972
19883643	BBA_00631	-1.11	1.28E-06	5.12E-06	down	D-alanine--D-alanine ligase
19884098	BBA_01086	-1.11	1.64E-12	1.16E-11	down	thymine dioxygenase
19888359	BBA_05347	-1.11	5.89E-11	3.73E-10	down	Ncp1-like protein

19888051	BBA_05039	-1.11	5.05E-15	4.25E-14	down	hypothetical protein BBA_05039
19887330	BBA_04318	-1.11	1.48E-14	1.21E-13	down	PH domain-containing protein
19885490	BBA_02478	-1.11	3.35E-07	1.44E-06	down	R3H domain-containing protein
19884250	BBA_01238	-1.11	1.08E-13	8.32E-13	down	hypothetical protein BBA_01238
19889004	BBA_05992	-1.11	1.38E-07	6.21E-07	down	NTF2 and RRM domain-containing protein
19886191	BBA_03179	-1.11	3.61E-11	2.32E-10	down	calcium-transporting ATPase
19889607	BBA_06595	-1.11	1.28E-03	3.07E-03	down	hypothetical protein UCP014753
19892090	BBA_09078	-1.11	1.90E-04	5.32E-04	down	beta-hexosaminidase
19883235	BBA_00223	-1.11	3.03E-04	8.20E-04	down	hypothetical protein BBA_00223
19892843	BBA_09831	-1.11	9.52E-04	2.35E-03	down	hypothetical protein BBA_09831
19884431	BBA_01419	-1.11	1.41E-15	1.23E-14	down	adenine phosphoribosyltransferase
19887676	BBA_04664	-1.10	1.11E-11	7.44E-11	down	pyridoxal-phosphate dependent enzyme
19883929	BBA_00917	-1.10	1.22E-13	9.41E-13	down	hypothetical protein BBA_00917
19887928	BBA_04916	-1.10	7.20E-14	5.61E-13	down	aminotransferase class-III
19885161	BBA_02149	-1.10	2.15E-08	1.06E-07	down	phosphate metabolism protein
19888403	BBA_05391	-1.10	2.95E-15	2.52E-14	down	modin protein
19891858	BBA_08846	-1.10	6.36E-14	4.97E-13	down	hypothetical protein BBA_08846
19883433	BBA_00421	-1.10	2.66E-11	1.74E-10	down	LAS seventeen-binding protein
19884099	BBA_01087	-1.10	3.55E-15	3.03E-14	down	amidohydrolase-like protein
19890790	BBA_07778	-1.10	2.30E-13	1.73E-12	down	oxidoreductase, 2OG-Fe(II) oxygenase family protein
19888070	BBA_05058	-1.10	3.10E-09	1.69E-08	down	acyltransferase-like protein
19893185	BBA_10173	-1.10	5.79E-13	4.24E-12	down	K-3-type glutaminase
19889170	BBA_06158	-1.10	6.67E-10	3.85E-09	down	fork head domain-containing protein
19889391	BBA_06379	-1.10	5.86E-09	3.10E-08	down	glycoside hydrolase family 16 protein
19885047	BBA_02035	-1.10	3.89E-10	2.29E-09	down	hypothetical protein BBA_02035
19889833	BBA_06821	-1.09	2.10E-07	9.25E-07	down	hypothetical protein BBA_06821
19891643	BBA_08631	-1.09	1.63E-07	7.25E-07	down	hypothetical protein BBA_08631
19892479	BBA_09467	-1.09	5.17E-09	2.75E-08	down	hypothetical protein BBA_09467
19890722	BBA_07710	-1.09	1.01E-04	2.98E-04	down	hypothetical protein BBA_07710
19892045	BBA_09033	-1.09	9.05E-13	6.51E-12	down	hypothetical protein BBA_09033
19886615	BBA_03603	-1.09	3.11E-10	1.85E-09	down	stomatin-like protein
19886476	BBA_03464	-1.09	1.83E-13	1.39E-12	down	C6 finger domain protein
19884097	BBA_01085	-1.09	1.16E-07	5.29E-07	down	peptidase family M1
19886869	BBA_03857	-1.09	3.58E-13	2.66E-12	down	hypothetical protein BBA_03857
19886285	BBA_03273	-1.09	1.37E-15	1.19E-14	down	hypothetical protein BBA_03273
19891518	BBA_08506	-1.08	1.45E-15	1.26E-14	down	multidrug and toxin extrusion protein
19892219	BBA_09207	-1.08	1.55E-02	2.91E-02	down	TPR repeat protein
19886511	BBA_03499	-1.08	9.42E-06	3.29E-05	down	hypothetical protein BBA_03499
19887969	BBA_04957	-1.08	2.30E-10	1.38E-09	down	potassium/sodium efflux P-type ATPase
19883318	BBA_00306	-1.08	2.04E-07	9.01E-07	down	DEAD/DEAH box helicase
19883783	BBA_00771	-1.08	3.00E-04	8.14E-04	down	Ankyrin repeat protein
19883618	BBA_00606	-1.08	1.53E-13	1.17E-12	down	hypothetical protein BBA_00606
19886503	BBA_03491	-1.08	4.23E-14	3.35E-13	down	tousled-like kinase
19889489	BBA_06477	-1.08	2.51E-05	8.13E-05	down	hypothetical protein BBA_06477
19887673	BBA_04661	-1.08	5.90E-12	4.01E-11	down	hypothetical protein BBA_04661
19885434	BBA_02422	-1.08	9.47E-05	2.79E-04	down	hypothetical protein BBA_02422
19886006	BBA_02994	-1.08	2.59E-12	1.79E-11	down	glycosyltransferase family 20
19891089	BBA_08077	-1.08	1.02E-10	6.35E-10	down	hypothetical protein BBA_08077
19884262	BBA_01250	-1.08	1.45E-05	4.86E-05	down	DNA polymerase II large subunit-like protein
19885044	BBA_02032	-1.08	5.99E-07	2.49E-06	down	dipeptidyl peptidase III
19891170	BBA_08158	-1.08	6.68E-06	2.38E-05	down	ATP-dependent protease La domain-containing protein
19889036	BBA_06024	-1.07	6.32E-06	2.26E-05	down	hypothetical protein BBA_06024
19889071	BBA_06059	-1.07	1.73E-13	1.32E-12	down	monooxygenase-like protein
19892094	BBA_09082	-1.07	3.61E-05	1.14E-04	down	acetylornithine deacetylase
19889389	BBA_06377	-1.07	8.02E-09	4.18E-08	down	RasGEF domain-containing protein
19883102	BBA_00090	-1.07	2.17E-09	1.20E-08	down	aryl-alcohol oxidase/vanillyl-alcohol oxidase
19883862	BBA_00850	-1.07	1.27E-10	7.77E-10	down	
19883358	BBA_00346	-1.07	2.05E-13	1.56E-12	down	fructosyl amino acid oxidase
19883637	BBA_00625	-1.07	1.99E-08	9.92E-08	down	Glycoside hydrolase, catalytic core
19885692	BBA_02680	-1.06	4.29E-14	3.39E-13	down	hypothetical protein BBA_02680
19886865	BBA_03853	-1.06	2.50E-06	9.51E-06	down	hypothetical protein BBA_03853

19892556	BBA_09544	-1.06	4.06E-07	1.73E-06	down	restless-like transposase
19885805	BBA_02793	-1.06	1.15E-12	8.18E-12	down	ankyrin repeat domain-containing protein 28
19883860	BBA_00848	-1.06	6.98E-11	4.38E-10	down	copper homeostasis protein cutC
19891265	BBA_08253	-1.06	1.86E-03	4.31E-03	down	2,3-cyclic-nucleotide 2-phosphodiesterase
19886452	BBA_03440	-1.06	9.95E-13	7.14E-12	down	hypothetical protein BBA_03440
19890939	BBA_07927	-1.06	2.45E-13	1.84E-12	down	C2 domain-containing protein
19888760	BBA_05748	-1.06	2.14E-04	5.95E-04	down	hypothetical protein BBA_05748
19892612	BBA_09600	-1.06	5.19E-09	2.77E-08	down	FluG domain-containing protein
19891505	BBA_08493	-1.06	1.53E-07	6.83E-07	down	Acyl-CoA N-acyltransferase
19886639	BBA_03627	-1.06	1.96E-14	1.60E-13	down	Hsp90 associated co-chaperone
19887986	BBA_04974	-1.06	4.15E-09	2.23E-08	down	aminotransferase family protein (LolT)
19889086	BBA_06074	-1.06	2.03E-07	8.95E-07	down	extensin-like protein
19893365	BBA_10353	-1.06	5.46E-07	2.29E-06	down	reverse transcriptase
19889807	BBA_06795	-1.05	4.04E-03	8.72E-03	down	hypothetical protein BBA_06795
19891119	BBA_08107	-1.05	1.54E-05	5.14E-05	down	MIF4G domain-containing protein
19891145	BBA_08133	-1.05	1.04E-12	7.43E-12	down	U-box domain-containing protein
19893338	BBA_10326	-1.05	2.34E-04	6.46E-04	down	hypothetical protein BBA_10326
19885771	BBA_02759	-1.05	2.17E-07	9.52E-07	down	hypothetical protein BBA_02759
19890743	BBA_07731	-1.05	9.96E-13	7.14E-12	down	ankyrin repeat domain protein
19890130	BBA_07118	-1.05	4.27E-12	2.92E-11	down	bacterial-type extracellular deoxyribonuclease
19890561	BBA_07549	-1.05	1.75E-07	7.77E-07	down	MFS transporter
19885612	BBA_02600	-1.05	1.29E-04	3.73E-04	down	MFS multidrug transporter
19886439	BBA_03427	-1.05	2.06E-06	7.94E-06	down	tricarballylate dehydrogenase
19883629	BBA_00617	-1.05	3.12E-08	1.52E-07	down	ankyrin repeat protein
19883839	BBA_00827	-1.05	3.92E-06	1.45E-05	down	RNA polymerase III RPC4
19886801	BBA_03789	-1.05	1.09E-03	2.65E-03	down	hypothetical protein BBA_03789
19887622	BBA_04610	-1.05	2.78E-09	1.52E-08	down	tRNA wybutosine-synthesizing protein
19883947	BBA_00935	-1.04	1.08E-13	8.32E-13	down	putative histidine acid phosphatase
19884160	BBA_01148	-1.04	4.66E-04	1.22E-03	down	hypothetical protein BBA_01148
19887932	BBA_04920	-1.04	3.71E-12	2.55E-11	down	hypothetical protein BBA_04920
19884608	BBA_01596	-1.04	2.74E-03	6.12E-03	down	hypothetical protein BBA_01596
19890647	BBA_07635	-1.04	3.84E-09	2.08E-08	down	SAM-dependent methyltransferase
19890623	BBA_07611	-1.04	1.32E-06	5.26E-06	down	non-ribosomal peptide synthetase
19889532	BBA_06520	-1.04	2.64E-07	1.14E-06	down	calcineurin-like phosphoesterase
19888833	BBA_05821	-1.04	1.38E-12	9.78E-12	down	oxidoreductase family protein
19892785	BBA_09773	-1.04	1.03E-11	6.91E-11	down	Cytochrome P450 CYP620D1
19885924	BBA_02912	-1.04	6.39E-10	3.70E-09	down	CoA binding domain-containing protein
19885768	BBA_02756	-1.04	2.35E-10	1.41E-09	down	TPR Domain containing protein
19884016	BBA_01004	-1.04	2.28E-07	9.94E-07	down	NUDIX domain-containing protein
19889480	BBA_06468	-1.04	1.35E-09	7.62E-09	down	ABC transporter
19889861	BBA_06849	-1.04	8.57E-10	4.88E-09	down	thermotolerance protein
19891169	BBA_08157	-1.04	5.88E-04	1.51E-03	down	hypothetical protein BBA_08157
19886201	BBA_03189	-1.04	4.67E-07	1.97E-06	down	structural toxin protein RtxA
19884466	BBA_01454	-1.04	4.36E-13	3.22E-12	down	aldehyde dehydrogenase
19885287	BBA_02275	-1.03	6.45E-06	2.30E-05	down	Cytochrome P450 CYP5202A1
19885456	BBA_02444	-1.03	5.09E-06	1.85E-05	down	OHCU decarboxylase
19888903	BBA_05891	-1.03	3.92E-10	2.31E-09	down	hypothetical protein BBA_05891
19893318	BBA_10306	-1.03	2.27E-07	9.93E-07	down	hypothetical protein BBA_10306
19891489	BBA_08477	-1.03	3.56E-11	2.29E-10	down	ATG C terminal domain-containing protein
19883577	BBA_00565	-1.03	2.89E-11	1.88E-10	down	annexin-like protein
19887490	BBA_04478	-1.03	4.47E-06	1.64E-05	down	ankyrin repeat protein
19890209	BBA_07197	-1.03	4.70E-14	3.69E-13	down	Peptidase M19, renal dipeptidase
19889972	BBA_06960	-1.03	1.53E-11	1.01E-10	down	alternative oxidase
19885045	BBA_02033	-1.03	1.50E-13	1.15E-12	down	feruloyl esterase A precursor
19892456	BBA_09444	-1.03	8.09E-12	5.44E-11	down	hypothetical protein BBA_09444
19888922	BBA_05910	-1.02	1.08E-10	6.68E-10	down	isoprenylcysteine carboxyl methyltransferase
19890696	BBA_07684	-1.02	6.76E-04	1.72E-03	down	BTB/POZ domain protein
19887315	BBA_04303	-1.02	1.01E-07	4.63E-07	down	LYAR-type C2HC zinc finger protein
19890312	BBA_07300	-1.02	6.49E-05	1.96E-04	down	C6 transcription factor
19891098	BBA_08086	-1.02	2.89E-13	2.16E-12	down	hypothetical protein BBA_08086
19884441	BBA_01429	-1.02	1.72E-06	6.71E-06	down	GTPase-activator protein for Ras-like GTPase

19885217	BBA_02205	-1.02	2.90E-05	9.31E-05	down	G-protein coupled receptor
19891093	BBA_08081	-1.02	3.09E-08	1.51E-07	down	La domain-containing protein
19889572	BBA_06560	-1.02	2.21E-12	1.54E-11	down	ABC transporter
19884614	BBA_01602	-1.02	3.59E-05	1.14E-04	down	kynurenine 3-monooxygenase
19888839	BBA_05827	-1.02	3.09E-12	2.13E-11	down	siderophore iron transporter
19889582	BBA_06570	-1.01	2.39E-09	1.31E-08	down	Ankyrin repeat protein
19888865	BBA_05853	-1.01	8.21E-03	1.65E-02	down	LysM domain-containing protein
19892480	BBA_09468	-1.01	5.94E-11	3.75E-10	down	WSC domain-containing protein
19883367	BBA_00355	-1.01	2.91E-13	2.17E-12	down	alanine racemase domain protein
19891966	BBA_08954	-1.01	1.15E-05	3.95E-05	down	retrotransposon protein, putative, Ty1-copia subclass
19883208	BBA_00196	-1.01	5.81E-03	1.21E-02	down	tat pathway signal sequence
19884192	BBA_01180	-1.01	8.07E-11	5.03E-10	down	homeobox domain-containing protein
19891664	BBA_08652	-1.01	4.93E-09	2.63E-08	down	transcription factor tfiiic complex subunit sfc6
19892345	BBA_09333	-1.01	2.23E-04	6.17E-04	down	C2H2 finger domain protein, putative
19892539	BBA_09527	-1.01	4.46E-07	1.88E-06	down	asparagine synthetase
19890105	BBA_07093	-1.01	5.01E-05	1.55E-04	down	RING finger protein
19888516	BBA_05504	-1.01	9.98E-11	6.19E-10	down	Immunoglobulin E-set
19891435	BBA_08423	-1.01	1.13E-05	3.88E-05	down	lipase-like protein
19891403	BBA_08391	-1.01	1.98E-11	1.30E-10	down	thiamine pyrophosphate enzyme
19883678	BBA_00666	-1.01	4.27E-06	1.57E-05	down	hypothetical protein BBA_00666
19888932	BBA_05920	-1.01	2.06E-09	1.14E-08	down	hypothetical protein BBA_05920
19892303	BBA_09291	-1.01	1.10E-04	3.21E-04	down	nucleoplasmin-like protein
19891800	BBA_08788	-1.00	1.72E-06	6.72E-06	down	MFS sugar transporter, putative
19883710	BBA_00698	-1.00	5.96E-10	3.46E-09	down	restless-like transposase
19892737	BBA_09725	1.00	7.33E-09	3.84E-08	up	integral membrane protein, putative
19887484	BBA_04472	1.00	9.64E-12	6.46E-11	up	PET8 protein
19885917	BBA_02905	1.00	1.46E-04	4.18E-04	up	protein transport protein YOS1
19888049	BBA_05037	1.01	2.44E-07	1.06E-06	up	cyclophilin A
19889843	BBA_06831	1.01	1.91E-09	1.06E-08	up	major facilitator superfamily transporter
19883451	BBA_00439	1.01	8.34E-11	5.19E-10	up	alanyl-tRNA synthetase
19884891	BBA_01879	1.01	4.63E-14	3.64E-13	up	glycosyltransferase family 17
19888444	BBA_05432	1.01	8.03E-15	6.71E-14	up	hypothetical protein BBA_05432
19890760	BBA_07748	1.01	1.51E-11	1.00E-10	up	ABC transporter
19891398	BBA_08386	1.01	8.60E-18	8.44E-17	up	pyruvate decarboxylase
19887693	BBA_04681	1.01	1.25E-10	7.63E-10	up	haloacid dehalogenase
19888820	BBA_05808	1.01	2.08E-15	1.79E-14	up	cell wall protein
19889098	BBA_06086	1.01	4.82E-07	2.03E-06	up	
19884123	BBA_01111	1.02	1.57E-15	1.36E-14	up	Cysteine synthase
19892615	BBA_09603	1.02	5.02E-06	1.82E-05	up	transposase-like protein
19890579	BBA_07567	1.02	9.21E-13	6.62E-12	up	nonspecific lipid-transfer protein
19891225	BBA_08213	1.02	4.96E-15	4.18E-14	up	hypothetical protein BBA_08213
19885174	BBA_02162	1.02	3.78E-06	1.40E-05	up	hypothetical protein BBA_02162
19890415	BBA_07403	1.02	5.80E-18	5.72E-17	up	hypothetical protein BBA_07403
19889954	BBA_06942	1.02	4.14E-06	1.53E-05	up	MFS multidrug transporter, putative
19887134	BBA_04122	1.02	2.40E-08	1.18E-07	up	MAK1-like monooxygenase
19883532	BBA_00520	1.02	1.52E-16	1.39E-15	up	heterokaryon incompatibility protein Het-C
19887680	BBA_04668	1.03	5.98E-11	3.78E-10	up	phenylacrylic acid decarboxylase, putative
19891621	BBA_08609	1.03	4.08E-09	2.20E-08	up	hypothetical protein BBA_08609
19889435	BBA_06423	1.03	4.62E-15	3.91E-14	up	cation transport ATPase
19892385	BBA_09373	1.03	1.71E-16	1.56E-15	up	inorganic pyrophosphatase
19886076	BBA_03064	1.03	4.16E-08	1.99E-07	up	hypothetical protein BBA_03064
19892152	BBA_09140	1.03	1.78E-17	1.71E-16	up	hypothetical protein BBA_09140
19891981	BBA_08969	1.03	2.23E-09	1.23E-08	up	hypothetical protein BBA_08969
19883025	BBA_00013	1.04	1.33E-12	9.42E-12	up	cellobiose dehydrogenase, putative
19884610	BBA_01598	1.04	2.89E-18	2.91E-17	up	proclavaminate amidinohydrolase
19889888	BBA_06876	1.04	3.18E-13	2.37E-12	up	AGC kinase
19887559	BBA_04547	1.04	4.94E-17	4.64E-16	up	hypothetical protein BBA_04547
19891616	BBA_08604	1.04	4.53E-06	1.66E-05	up	hypothetical protein BBA_08604
19886888	BBA_03876	1.04	1.80E-10	1.09E-09	up	hypothetical protein BBA_03876
19891016	BBA_08004	1.04	3.30E-05	1.05E-04	up	complex 1 protein (LYR family) protein
19883399	BBA_00387	1.04	8.47E-13	6.12E-12	up	RPEL repeat protein

19889166	BBA_06154	1.04	8.82E-11	5.49E-10	up	triacylglycerol lipase
19892770	BBA_09758	1.04	4.43E-13	3.27E-12	up	serum paraoxonase/arylesterase family protein
19891348	BBA_08336	1.04	1.10E-18	1.13E-17	up	CAAX amino terminal protease
19886983	BBA_03971	1.04	8.66E-18	8.49E-17	up	Glycoside hydrolase, family 31
19887044	BBA_04032	1.04	5.99E-17	5.61E-16	up	hydrolase, alpha/beta fold family protein
19892224	BBA_09212	1.04	1.20E-07	5.47E-07	up	hypothetical protein BBA_09212
19888959	BBA_05947	1.05	2.09E-05	6.85E-05	up	short chain dehydrogenase
19891186	BBA_08174	1.05	5.28E-04	1.37E-03	up	putative Heat-labile enterotoxin IIB, A chain
19892415	BBA_09403	1.05	1.69E-04	4.78E-04	up	bZIP transcription factor
19884701	BBA_01689	1.05	1.44E-07	6.47E-07	up	Vacuolar ATP synthase subunit F
19892075	BBA_09063	1.05	1.15E-05	3.96E-05	up	hypothetical protein BBA_09063
19888836	BBA_05824	1.05	8.43E-08	3.89E-07	up	thioesterase superfamily protein
19888753	BBA_05741	1.05	1.10E-15	9.58E-15	up	4-hydroxyacetophenone monooxygenase
19888984	BBA_05972	1.05	8.74E-13	6.30E-12	up	hypothetical protein BBA_05972
19892709	BBA_09697	1.05	8.98E-09	4.65E-08	up	C2H2 transcription factor
19888056	BBA_05044	1.06	2.65E-12	1.84E-11	up	FAD monooxygenase
19885153	BBA_02141	1.06	1.96E-19	2.09E-18	up	mannitol-1-phosphate dehydrogenase
19891787	BBA_08775	1.06	1.78E-11	1.17E-10	up	6-O-methylguanine DNA methyltransferase
19893308	BBA_10296	1.06	1.23E-10	7.55E-10	up	reverse transcriptase
19892493	BBA_09481	1.06	4.50E-20	4.98E-19	up	quinate permease
19889613	BBA_06601	1.06	2.09E-10	1.25E-09	up	hypothetical protein BBA_06601
19885115	BBA_02103	1.06	8.76E-08	4.03E-07	up	spindle assembly checkpoint kinase
19886596	BBA_03584	1.06	4.04E-10	2.37E-09	up	ABC transporter
19887110	BBA_04098	1.06	1.52E-07	6.80E-07	up	hypothetical protein BBA_04098
19885225	BBA_02213	1.06	3.94E-13	2.92E-12	up	hypothetical protein BBA_02213
19885519	BBA_02507	1.06	6.15E-21	7.12E-20	up	fungal specific transcription factor
19883250	BBA_00238	1.06	1.42E-12	1.00E-11	up	chaperone protein hchA
19893238	BBA_10226	1.06	1.10E-05	3.81E-05	up	restless-like transposase
19892969	BBA_09957	1.07	2.64E-16	2.39E-15	up	hypothetical protein BBA_09957
19887508	BBA_04496	1.07	7.92E-07	3.23E-06	up	putative sugar transporter
19891355	BBA_08343	1.07	1.37E-04	3.93E-04	up	hypothetical protein BBA_08343
19889811	BBA_06799	1.07	1.27E-08	6.49E-08	up	hypothetical protein BBA_06799
19887779	BBA_04767	1.07	8.70E-07	3.54E-06	up	glycosyl hydrolase family 76
19883046	BBA_00034	1.07	1.56E-20	1.76E-19	up	group II pyridoxal-5-phosphate decarboxylase
19892559	BBA_09547	1.07	3.54E-11	2.28E-10	up	transposase-like protein
19888459	BBA_05447	1.08	2.03E-07	8.96E-07	up	
19890101	BBA_07089	1.08	2.86E-15	2.45E-14	up	exostosin 2
19887052	BBA_04040	1.08	1.70E-11	1.13E-10	up	MFS monocarboxylate transporter
19883311	BBA_00299	1.08	9.62E-16	8.44E-15	up	hypothetical protein BBA_00299
19886472	BBA_03460	1.08	1.10E-20	1.25E-19	up	phosphatidylinositol transfer protein CSR1
19891063	BBA_08051	1.08	1.34E-08	6.80E-08	up	hypothetical protein BBA_08051
19887384	BBA_04372	1.08	1.03E-17	1.01E-16	up	DNA repair protein (Rad57), putative
19893203	BBA_10191	1.08	2.05E-05	6.73E-05	up	hypothetical protein BBA_10191
19891590	BBA_08578	1.08	1.54E-09	8.64E-09	up	Acyl-CoA N-acyltransferase
19892912	BBA_09900	1.08	1.02E-04	3.00E-04	up	ankyrin 2,3/unc44
19884295	BBA_01283	1.09	9.74E-21	1.11E-19	up	major facilitator superfamily transporter
19891523	BBA_08511	1.09	3.77E-14	3.00E-13	up	short-chain dehydrogenase/reductase 2
19893264	BBA_10252	1.09	1.11E-05	3.84E-05	up	hypothetical protein BBA_10252
19883125	BBA_00113	1.09	7.72E-11	4.83E-10	up	hypothetical protein BBA_00113
19891422	BBA_08410	1.09	1.73E-12	1.22E-11	up	ppic-type ppiase domain-containing protein
19886487	BBA_03475	1.09	4.97E-08	2.36E-07	up	bicyclomycin resistance protein
19893019	BBA_10007	1.09	1.23E-05	4.20E-05	up	hypothetical protein BBA_10007
19888616	BBA_05604	1.09	1.61E-16	1.48E-15	up	hypothetical protein BBA_05604
19886686	BBA_03674	1.09	4.13E-12	2.83E-11	up	Formyl transferase
19891313	BBA_08301	1.09	3.37E-11	2.17E-10	up	hypothetical protein BBA_08301
19890856	BBA_07844	1.09	6.47E-16	5.72E-15	up	O-methyltransferase, family 3
19884705	BBA_01693	1.09	9.02E-21	1.03E-19	up	major facilitator superfamily transporter
19884605	BBA_01593	1.09	2.41E-20	2.71E-19	up	quinone oxidoreductase
19886725	BBA_03713	1.09	1.67E-09	9.29E-09	up	L-PSP endoribonuclease family protein
19890422	BBA_07410	1.09	1.44E-14	1.19E-13	up	glutathione S-transferase domain-containing protein
19888020	BBA_05008	1.09	9.34E-09	4.83E-08	up	hypothetical protein BBA_05008

19885027	BBA_02015	1.09	3.74E-13	2.78E-12	up	hypothetical protein BBA_02015
19885205	BBA_02193	1.10	4.51E-13	3.32E-12	up	hypothetical protein BBA_02193
19885439	BBA_02427	1.10	1.34E-07	6.03E-07	up	conidiation-specific protein (con-13)
19888762	BBA_05750	1.10	8.05E-09	4.20E-08	up	thioesterase superfamily protein
19883993	BBA_00981	1.10	5.17E-08	2.45E-07	up	hypothetical protein BBA_00981
19885566	BBA_02554	1.10	6.86E-07	2.83E-06	up	thioredoxin reductase GliT
19890578	BBA_07566	1.10	8.46E-17	7.86E-16	up	histidinol phosphate phosphatase HisJ family protein
19888617	BBA_05605	1.10	3.41E-19	3.60E-18	up	formyl transferase domain-containing protein
19893304	BBA_10292	1.10	4.15E-12	2.84E-11	up	cytochrome c oxidase assembly factor
19892531	BBA_09519	1.10	1.57E-07	7.02E-07	up	phosphotransferase enzyme family protein
19887527	BBA_04515	1.10	4.00E-08	1.92E-07	up	hypothetical protein BBA_04515
19892036	BBA_09024	1.10	1.28E-13	9.80E-13	up	transposase-like protein
19891565	BBA_08553	1.10	2.47E-07	1.07E-06	up	hypothetical protein BBA_08553
19892177	BBA_09165	1.11	2.93E-10	1.74E-09	up	tetratricopeptide-like protein
19885743	BBA_02731	1.11	2.30E-21	2.70E-20	up	hypothetical protein BBA_02731
19892716	BBA_09704	1.11	8.17E-21	9.41E-20	up	DUF89 domain protein
19886339	BBA_03327	1.11	7.74E-13	5.61E-12	up	YCII-related domain protein
19883225	BBA_00213	1.11	5.43E-13	3.99E-12	up	nitrilotriacetate monooxygenase component B
19885485	BBA_02473	1.11	2.54E-09	1.39E-08	up	ribosomal protein S19
19890385	BBA_07373	1.11	1.85E-12	1.30E-11	up	hypothetical protein BBA_07373
19886027	BBA_03015	1.11	3.74E-21	4.37E-20	up	class I hydrophobin
19886027	BBA_03015	1.11	3.74E-21	4.37E-20	up	class I hydrophobin
19886267	BBA_03255	1.11	5.65E-13	4.14E-12	up	hypothetical protein BBA_03255
19885034	BBA_02022	1.11	2.55E-20	2.86E-19	up	hypothetical protein BBA_02022
19893269	BBA_10257	1.12	5.58E-06	2.01E-05	up	hypothetical protein BBA_10257
19891992	BBA_08980	1.12	1.52E-04	4.32E-04	up	hypothetical protein BBA_08980
19891937	BBA_08925	1.12	2.52E-10	1.50E-09	up	O-methyltransferase, family 3
19883413	BBA_00401	1.12	8.76E-12	5.89E-11	up	hypothetical protein BBA_00401
19886427	BBA_03415	1.12	6.58E-08	3.07E-07	up	calcofluor white hypersensitive protein
19892209	BBA_09197	1.12	1.73E-11	1.14E-10	up	interferon-induced GTP-binding protein Mx
19883040	BBA_00028	1.12	4.37E-19	4.58E-18	up	oxysterol-binding family protein
19887041	BBA_04029	1.12	5.48E-20	6.01E-19	up	amino acid permease
19885375	BBA_02363	1.12	1.18E-17	1.14E-16	up	hypothetical protein BBA_02363
19890545	BBA_07533	1.12	3.12E-19	3.30E-18	up	NADP-dependent alcohol dehydrogenase
19883060	BBA_00048	1.13	5.03E-10	2.93E-09	up	hypothetical protein BBA_00048
19886592	BBA_03580	1.13	3.52E-22	4.27E-21	up	FkbM family methyltransferase
19885374	BBA_02362	1.13	1.93E-19	2.07E-18	up	C-5 cytosine-specific DNA methylase
19886946	BBA_03934	1.13	4.56E-22	5.52E-21	up	Cytochrome P450 CYP617A1
19889673	BBA_06661	1.13	5.32E-05	1.64E-04	up	nonribosomal peptide synthase, putative
19889686	BBA_06674	1.14	2.32E-07	1.01E-06	up	NADH:flavin oxidoreductase/NADH oxidase
19888684	BBA_05672	1.14	1.45E-16	1.34E-15	up	beta-glucosidase, putative
19893230	BBA_10218	1.14	8.93E-05	2.65E-04	up	
19887603	BBA_04591	1.14	1.04E-21	1.24E-20	up	glycerophosphoryl diester phosphodiesterase
19891436	BBA_08424	1.14	4.43E-13	3.27E-12	up	nonribosomal peptide synthase, putative
19884252	BBA_01240	1.14	5.37E-21	6.24E-20	up	LysR family regulatory protein
19889993	BBA_06981	1.14	3.18E-06	1.20E-05	up	cytochrome c oxidase assembly protein COX19
19884046	BBA_01034	1.14	1.79E-17	1.73E-16	up	DNA photolyase
19892939	BBA_09927	1.14	3.14E-04	8.47E-04	up	pol-like protein
19887418	BBA_04406	1.14	5.80E-23	7.32E-22	up	hypothetical protein BBA_04406
19888869	BBA_05857	1.15	7.95E-17	7.41E-16	up	short chain dehydrogenase
19891399	BBA_08387	1.15	2.06E-20	2.32E-19	up	aromatic amino acid aminotransferase
19886916	BBA_03904	1.15	7.24E-06	2.56E-05	up	SCP-like extracellular protein, putative
19885126	BBA_02114	1.15	3.09E-18	3.09E-17	up	mitochondrial phosphate carrier protein
19890344	BBA_07332	1.15	2.02E-06	7.80E-06	up	aspartyl proteinase
19893014	BBA_10002	1.16	7.76E-05	2.32E-04	up	hypothetical protein BBA_10002
19890170	BBA_07158	1.16	6.33E-11	3.99E-10	up	hypothetical protein BBA_07158
19892660	BBA_09648	1.16	4.19E-10	2.46E-09	up	hypothetical protein BBA_09648
19886019	BBA_03007	1.16	1.09E-12	7.78E-12	up	hypothetical protein BBA_03007
19888284	BBA_05272	1.16	6.90E-22	8.31E-21	up	ABC transporter
19887635	BBA_04623	1.16	6.44E-10	3.72E-09	up	ribosomal protein S14p/S29e
19888678	BBA_05666	1.16	3.99E-17	3.77E-16	up	MFS transporter

19886133	BBA_03121	1.17	5.88E-17	5.51E-16	up	cell surface protein
19888625	BBA_05613	1.17	6.07E-10	3.51E-09	up	hypothetical protein BBA_05613
19889579	BBA_06567	1.17	5.30E-12	3.61E-11	up	Catalase-like domain, heme-dependent
19889950	BBA_06938	1.17	1.94E-06	7.50E-06	up	phytanoyl-CoA dioxygenase family protein
19888589	BBA_05577	1.17	2.61E-23	3.35E-22	up	SCP-like extracellular protein
19883666	BBA_00654	1.17	3.53E-06	1.32E-05	up	hypothetical protein BBA_00654
19885090	BBA_02078	1.17	2.94E-09	1.60E-08	up	density-regulated protein DRP1
19892043	BBA_09031	1.17	1.48E-14	1.22E-13	up	hypothetical protein BBA_09031
19890252	BBA_07240	1.17	5.17E-08	2.45E-07	up	hypothetical protein BBA_07240
19885643	BBA_02631	1.18	6.50E-19	6.74E-18	up	hypothetical protein BBA_02631
19885809	BBA_02797	1.18	3.83E-17	3.62E-16	up	
19892279	BBA_09267	1.18	1.04E-06	4.20E-06	up	hypothetical protein BBA_09267
19891483	BBA_08471	1.18	4.69E-09	2.51E-08	up	MFS allantoate transporter, putative
19888388	BBA_05376	1.18	7.80E-22	9.33E-21	up	alpha-amylase A type-3
19892170	BBA_09158	1.18	3.13E-06	1.18E-05	up	hypothetical protein BBA_09158
19887637	BBA_04625	1.18	1.06E-07	4.83E-07	up	hypothetical protein BBA_04625
19892489	BBA_09477	1.19	4.44E-15	3.77E-14	up	hexose transporter
19883460	BBA_00448	1.19	1.07E-08	5.45E-08	up	hypothetical protein BBA_00448
19887732	BBA_04720	1.19	8.20E-16	7.21E-15	up	hypothetical protein BBA_04720
19886051	BBA_03039	1.19	2.92E-07	1.26E-06	up	flavin containing amine oxidoreductase
19891876	BBA_08864	1.19	1.12E-16	1.04E-15	up	hypothetical protein BBA_08864
19884647	BBA_01635	1.19	9.74E-07	3.94E-06	up	Cytochrome P450 CYP570E2
19887699	BBA_04687	1.19	2.63E-25	3.70E-24	up	nuclear migration protein
19883159	BBA_00147	1.19	2.62E-11	1.71E-10	up	cutinase-2 protein
19891575	BBA_08563	1.19	1.51E-20	1.71E-19	up	ABC transporter, putative
19890316	BBA_07304	1.20	1.95E-19	2.08E-18	up	malate dehydrogenase, putative
19892642	BBA_09630	1.20	5.88E-06	2.11E-05	up	hypothetical protein BBA_09630
19886765	BBA_03753	1.20	4.51E-23	5.71E-22	up	glutamate/Leucine/Phenylalanine/Valine dehydrogenase
19891531	BBA_08519	1.20	5.96E-10	3.46E-09	up	lactoylglutathione lyase
19886156	BBA_03144	1.20	1.44E-04	4.11E-04	up	short chain dehydrogenase
19888802	BBA_05790	1.20	1.25E-13	9.58E-13	up	hypothetical protein BBA_05790
19892997	BBA_09985	1.20	1.96E-07	8.65E-07	up	DUF124 domain protein
19886013	BBA_03001	1.20	3.85E-08	1.85E-07	up	ABC-type Fe3+ transport system
19886953	BBA_03941	1.20	7.01E-25	9.73E-24	up	hypothetical protein BBA_03941
19887112	BBA_04100	1.20	3.94E-15	3.35E-14	up	GMC oxidoreductase
19892061	BBA_09049	1.21	1.38E-18	1.41E-17	up	RING finger domain protein
19885493	BBA_02481	1.21	2.65E-13	1.98E-12	up	DUF636 domain-containing protein
19883204	BBA_00192	1.21	3.40E-08	1.64E-07	up	Ribonuclease H1
19886314	BBA_03302	1.21	7.76E-15	6.50E-14	up	histidinolphosphatase-like protein
19893131	BBA_10119	1.21	2.39E-15	2.04E-14	up	HAD-superfamily subfamily IIA hydrolase
19893346	BBA_10334	1.21	1.11E-12	7.93E-12	up	hypothetical protein BBA_10334
19890596	BBA_07584	1.21	2.75E-14	2.21E-13	up	MAP kinase kinase skh1/pek1
19890879	BBA_07867	1.22	1.39E-06	5.51E-06	up	hypothetical protein BBA_07867
19892638	BBA_09626	1.22	2.10E-05	6.89E-05	up	hypothetical protein BBA_09626
19887200	BBA_04188	1.22	5.10E-04	1.32E-03	up	tat pathway signal sequence
19888282	BBA_05270	1.22	3.35E-11	2.16E-10	up	lipase class 2
19884572	BBA_01560	1.22	5.57E-12	3.79E-11	up	triose-phosphate transporter
19887073	BBA_04061	1.22	1.39E-23	1.80E-22	up	tyrosinase 2
19892536	BBA_09524	1.22	4.84E-18	4.79E-17	up	agmatine deiminase
19892351	BBA_09339	1.22	2.61E-17	2.49E-16	up	CFEM domain-containing protein
19889219	BBA_06207	1.22	1.72E-12	1.21E-11	up	hypothetical protein BBA_06207
19884976	BBA_01964	1.22	1.20E-24	1.64E-23	up	ferric-chelate reductase
19890788	BBA_07776	1.22	8.12E-17	7.55E-16	up	hypothetical protein BBA_07776
19888700	BBA_05688	1.22	2.14E-07	9.41E-07	up	Arylsulfotransferase-like protein
19885695	BBA_02683	1.23	2.22E-07	9.70E-07	up	hypothetical protein BBA_02683
19884673	BBA_01661	1.23	7.33E-17	6.84E-16	up	expression library immunization antigen 1
19890531	BBA_07519	1.23	5.62E-13	4.12E-12	up	glucose sorbosone dehydrogenase
19883775	BBA_00763	1.23	7.66E-26	1.10E-24	up	glutaryl-CoA dehydrogenase
19887058	BBA_04046	1.23	3.23E-23	4.13E-22	up	hypothetical protein BBA_04046
19888274	BBA_05262	1.24	8.87E-07	3.61E-06	up	WSC domain-containing protein
19884358	BBA_01346	1.24	6.00E-09	3.18E-08	up	hypothetical protein BBA_01346

19891190	BBA_08178	1.24	4.10E-14	3.25E-13	up	hydantoinase B/oxoprolinase
19891539	BBA_08527	1.24	1.39E-13	1.07E-12	up	hypothetical protein BBA_08527
19884063	BBA_01051	1.25	4.44E-16	3.95E-15	up	tat pathway signal sequence
19888674	BBA_05662	1.25	1.27E-16	1.17E-15	up	Alpha/beta hydrolase fold-3
19891081	BBA_08069	1.25	1.83E-06	7.11E-06	up	glycosyl transferase
19888055	BBA_05043	1.25	3.88E-06	1.44E-05	up	taurine catabolism dioxygenase TauD
19888387	BBA_05375	1.26	8.66E-15	7.22E-14	up	ferric reductase transmembrane component 4
19887936	BBA_04924	1.26	1.03E-22	1.29E-21	up	meiotic chromosome segregation protein
19886332	BBA_03320	1.26	1.62E-08	8.18E-08	up	hypothetical protein BBA_03320
19883748	BBA_00736	1.26	2.62E-18	2.64E-17	up	hypothetical protein BBA_00736
19890633	BBA_07621	1.26	4.52E-24	5.97E-23	up	hypothetical protein BBA_07621
19890294	BBA_07282	1.26	2.18E-23	2.81E-22	up	pristinamycin IIA synthase subunit A
19887929	BBA_04917	1.26	6.12E-08	2.87E-07	up	mannosylphosphorylation protein MNN4
19884638	BBA_01626	1.26	3.00E-18	3.01E-17	up	helix-loop-helix DNA-binding domain-containing protein
19892879	BBA_09867	1.26	1.62E-18	1.65E-17	up	hypothetical protein BBA_09867
19883658	BBA_00646	1.26	4.81E-05	1.49E-04	up	hypothetical protein BBA_00646
19884690	BBA_01678	1.27	4.54E-15	3.85E-14	up	ZIP Zinc transporter family protein
19888963	BBA_05951	1.27	2.90E-08	1.42E-07	up	hypothetical protein BBA_05951
19883483	BBA_00471	1.27	4.45E-24	5.90E-23	up	catechol O-methyltransferase
19892434	BBA_09422	1.27	2.26E-08	1.12E-07	up	hypothetical protein BBA_09422
19885004	BBA_01992	1.27	3.12E-27	4.72E-26	up	calcineurin-like phosphoesterase
19883342	BBA_00330	1.28	4.14E-26	5.99E-25	up	succinate-semialdehyde dehydrogenase NADP+
19885367	BBA_02355	1.28	8.91E-08	4.10E-07	up	hypothetical protein BBA_02355
19888167	BBA_05155	1.28	4.17E-27	6.27E-26	up	extracellular aldonolactonase
19887696	BBA_04684	1.28	3.14E-27	4.74E-26	up	tetratricopeptide repeat domain protein
19890634	BBA_07622	1.28	1.01E-14	8.38E-14	up	yjfJ-like protein
19892649	BBA_09637	1.28	4.22E-27	6.34E-26	up	hypothetical protein BBA_09637
19892280	BBA_09268	1.28	1.10E-04	3.22E-04	up	hypothetical protein BBA_09268
19887740	BBA_04728	1.29	9.77E-20	1.06E-18	up	coenzyme A transferase
19884202	BBA_01190	1.29	5.24E-26	7.55E-25	up	fumarylacetoacetate hydrolase
19893256	BBA_10244	1.29	4.82E-07	2.03E-06	up	hypothetical protein BBA_10244
19892146	BBA_09134	1.29	1.03E-10	6.38E-10	up	cofilin/tropomyosin-type actin-binding protein
19884492	BBA_01480	1.29	4.55E-24	6.00E-23	up	major facilitator superfamily transporter
19886276	BBA_03264	1.29	7.66E-10	4.39E-09	up	NADH:ubiquinone oxidoreductase 6.6kD subunit
19892227	BBA_09215	1.29	1.86E-16	1.69E-15	up	hypothetical protein BBA_09215
19891599	BBA_08587	1.30	4.87E-09	2.60E-08	up	hypothetical protein BBA_08587
19883563	BBA_00551	1.30	4.31E-24	5.72E-23	up	major facilitator superfamily transporter
19886661	BBA_03649	1.30	2.87E-09	1.56E-08	up	glucose-repressible protein
19885376	BBA_02364	1.30	1.09E-23	1.41E-22	up	DJ-1/PfpI family protein
19888752	BBA_05740	1.30	1.11E-10	6.81E-10	up	major facilitator superfamily transporter
19891772	BBA_08760	1.30	1.01E-15	8.86E-15	up	OsmC-like protein
19890511	BBA_07499	1.30	8.31E-05	2.47E-04	up	hypothetical protein BBA_07499
19883059	BBA_00047	1.31	4.08E-23	5.18E-22	up	Cel5b-like protein
19891686	BBA_08674	1.31	5.87E-06	2.11E-05	up	hypothetical protein BBA_08674
19890914	BBA_07902	1.31	3.50E-14	2.80E-13	up	sterol desaturase
19885802	BBA_02790	1.31	1.66E-22	2.05E-21	up	hypothetical protein BBA_02790
19892756	BBA_09744	1.31	7.21E-10	4.14E-09	up	O-methyltransferase-like protein
19883456	BBA_00444	1.31	1.86E-25	2.63E-24	up	nucleoside transporter, putative
19890793	BBA_07781	1.32	3.47E-22	4.23E-21	up	F-box and wd40 domain protein
19889610	BBA_06598	1.32	1.63E-08	8.22E-08	up	histidine acid phosphatase
19891183	BBA_08171	1.32	3.29E-28	5.10E-27	up	adenylosuccinate synthetase
19892677	BBA_09665	1.32	1.36E-10	8.27E-10	up	hypothetical protein BBA_09665
19890955	BBA_07943	1.32	1.23E-29	1.98E-28	up	glycerophosphoryl diester phosphodiesterase
19889595	BBA_06583	1.32	1.76E-07	7.84E-07	up	hypothetical protein BBA_06583
19883651	BBA_00639	1.32	9.16E-13	6.59E-12	up	hypothetical protein BBA_00639
19887391	BBA_04379	1.32	8.36E-23	1.05E-21	up	proline-rich protein
19883295	BBA_00283	1.32	3.76E-07	1.60E-06	up	membrane transporter
19893079	BBA_10067	1.33	4.36E-08	2.08E-07	up	DSBA-like thioredoxin domain protein
19886687	BBA_03675	1.33	6.58E-17	6.15E-16	up	NAD dependent epimerase/dehydratase
19888805	BBA_05793	1.33	1.80E-09	9.97E-09	up	hypothetical protein BBA_05793
19886708	BBA_03696	1.33	1.30E-18	1.33E-17	up	hypothetical protein BBA_03696

19892383	BBA_09371	1.33	4.64E-06	1.70E-05	up	hypothetical protein BBA_09371
19890727	BBA_07715	1.34	2.29E-13	1.73E-12	up	major facilitator superfamily transporter
19891740	BBA_08728	1.34	1.05E-19	1.15E-18	up	hexose transporter
19889771	BBA_06759	1.34	1.39E-06	5.51E-06	up	hypothetical protein BBA_06759
19892218	BBA_09206	1.34	5.55E-11	3.52E-10	up	SNARE domain-containing protein
19888816	BBA_05804	1.34	7.96E-11	4.97E-10	up	hypothetical protein BBA_05804
19883450	BBA_00438	1.35	3.43E-10	2.03E-09	up	GNAT family acetyltransferase, putative
19891051	BBA_08039	1.35	1.04E-10	6.41E-10	up	shwachman-Bodian-Diamond syndrome protein
19887026	BBA_04014	1.35	1.42E-11	9.45E-11	up	inositol phospholipid synthesis protein Scs3p
19889560	BBA_06548	1.35	5.10E-29	8.08E-28	up	transient receptor potential (TRP) ion channel
19887502	BBA_04490	1.35	8.39E-21	9.65E-20	up	hypothetical protein BBA_04490
19889364	BBA_06352	1.35	1.65E-04	4.66E-04	up	glycoside hydrolase family 76
19889141	BBA_06129	1.35	6.63E-12	4.49E-11	up	hypothetical protein BBA_06129
19891437	BBA_08425	1.36	2.03E-22	2.50E-21	up	Cytochrome P450 CYP5262A3
19891667	BBA_08655	1.36	4.22E-07	1.79E-06	up	hypothetical protein BBA_08655
19892265	BBA_09253	1.36	1.92E-20	2.16E-19	up	XFP domain-containing protein MPK1
19890072	BBA_07060	1.37	2.12E-08	1.05E-07	up	hypothetical protein BBA_07060
19887176	BBA_04164	1.37	7.02E-08	3.27E-07	up	phosphorylcholine phosphatase
19885763	BBA_02751	1.37	4.33E-07	1.83E-06	up	Cytochrome P450 CYP5282A1
19891980	BBA_08968	1.37	2.71E-24	3.63E-23	up	hypothetical protein BBA_08968
19883631	BBA_00619	1.37	4.38E-18	4.35E-17	up	intracellular serine protease
19892267	BBA_09255	1.38	1.07E-20	1.22E-19	up	acetate kinase
19883543	BBA_00531	1.38	3.71E-14	2.95E-13	up	hypothetical protein BBA_00531
19884873	BBA_01861	1.38	2.38E-27	3.59E-26	up	hypothetical protein BBA_01861
19886726	BBA_03714	1.38	1.51E-13	1.16E-12	up	ricin-type beta-trefoil lectin domain-containing protein
19886077	BBA_03065	1.38	2.98E-17	2.83E-16	up	GliK protein
19891501	BBA_08489	1.39	3.51E-14	2.80E-13	up	DJ-1/PfpI family protein
19887135	BBA_04123	1.39	3.73E-21	4.36E-20	up	NmrA-like family protein
19893290	BBA_10278	1.39	8.81E-09	4.56E-08	up	hypothetical protein BBA_10278
19892487	BBA_09475	1.40	8.10E-05	2.42E-04	up	hypothetical protein BBA_09475
19885173	BBA_02161	1.40	5.19E-25	7.26E-24	up	hypothetical protein BBA_02161
19883774	BBA_00762	1.40	5.85E-10	3.40E-09	up	hypothetical protein BBA_00762
19887048	BBA_04036	1.40	3.83E-11	2.46E-10	up	NmrA-like family protein
19887166	BBA_04154	1.40	1.16E-22	1.44E-21	up	hypothetical protein BBA_04154
19885788	BBA_02776	1.41	5.69E-30	9.37E-29	up	choline transport protein
19892636	BBA_09624	1.41	1.91E-14	1.56E-13	up	hypothetical protein BBA_09624
19884691	BBA_01679	1.41	4.02E-04	1.06E-03	up	hypothetical protein BBA_01679
19886325	BBA_03313	1.41	3.53E-06	1.32E-05	up	hypothetical protein BBA_03313
19884490	BBA_01478	1.41	5.73E-34	1.07E-32	up	hydantoinase B/oxoprolinase
19890161	BBA_07149	1.41	2.47E-08	1.22E-07	up	major facilitator superfamily transporter
19892016	BBA_09004	1.41	7.49E-22	8.99E-21	up	Putative C2H2 finger domain transcription factor
19892203	BBA_09191	1.41	1.46E-10	8.88E-10	up	MIP transporter
19891012	BBA_08000	1.42	3.82E-23	4.86E-22	up	enoyl-CoA hydratase/isomerase
19893237	BBA_10225	1.42	5.27E-20	5.80E-19	up	C6 finger domain protein, putative
19889152	BBA_06140	1.42	8.36E-09	4.34E-08	up	hypothetical protein BBA_06140
19889648	BBA_06636	1.43	1.51E-08	7.62E-08	up	hypothetical protein BBA_06636
19889654	BBA_06642	1.43	7.36E-06	2.60E-05	up	hypothetical protein BBA_06642
19884790	BBA_01778	1.43	6.34E-08	2.96E-07	up	hypothetical protein BBA_01778
19893359	BBA_10347	1.43	2.38E-32	4.27E-31	up	FAD binding domain-containing protein
19883630	BBA_00618	1.43	2.74E-12	1.89E-11	up	Hemolysin-III protein
19887407	BBA_04395	1.44	2.90E-24	3.88E-23	up	hypothetical protein BBA_04395
19883699	BBA_00687	1.44	2.62E-17	2.50E-16	up	hypothetical protein BBA_00687
19893153	BBA_10141	1.45	7.36E-08	3.42E-07	up	hypothetical protein BBA_10141
19885123	BBA_02111	1.45	1.22E-22	1.52E-21	up	NAD dependent epimerase/dehydratase
19891061	BBA_08049	1.46	2.20E-14	1.80E-13	up	VMA21-like domain-containing protein
19887847	BBA_04835	1.46	2.09E-12	1.46E-11	up	hypothetical protein BBA_04835
19892173	BBA_09161	1.46	1.72E-12	1.21E-11	up	NACHT and WD domain protein
19893226	BBA_10214	1.46	1.79E-18	1.82E-17	up	hypothetical protein BBA_10214
19886415	BBA_03403	1.46	5.19E-08	2.46E-07	up	thioredoxin-like protein
19889774	BBA_06762	1.46	4.81E-26	6.94E-25	up	ctr copper transporter
19891438	BBA_08426	1.46	1.11E-24	1.52E-23	up	aspartate aminotransferase, putative

19886122	BBA_03110	1.47	9.55E-18	9.35E-17	up	OPT oligopeptide transporter
19884006	BBA_00994	1.47	3.15E-25	4.41E-24	up	membrane protein-like protein
19890430	BBA_07418	1.47	1.50E-04	4.26E-04	up	fungal specific transcription factor
19891188	BBA_08176	1.47	4.85E-11	3.09E-10	up	mitochondrial CorA family metal ion transporter
19885925	BBA_02913	1.47	8.02E-24	1.05E-22	up	methyltransferase domain-containing protein
19889032	BBA_06020	1.48	5.54E-19	5.77E-18	up	hypothetical protein BBA_06020
19892027	BBA_09015	1.48	2.48E-04	6.80E-04	up	lipase/esterase, putative
19888996	BBA_05984	1.48	3.54E-26	5.14E-25	up	pH regulatory protein
19888679	BBA_05667	1.48	2.69E-07	1.16E-06	up	hypothetical protein BBA_05667
19893366	BBA_10354	1.48	2.39E-14	1.94E-13	up	gag protein
19887290	BBA_04278	1.48	4.30E-14	3.39E-13	up	pyridine nucleotide-disulfide oxidoreductase, putative
19885773	BBA_02761	1.48	7.52E-29	1.18E-27	up	
19885971	BBA_02959	1.49	1.40E-14	1.15E-13	up	hypothetical protein BBA_02959
19886591	BBA_03579	1.49	5.85E-36	1.18E-34	up	putative apoptosis-inducing factor (AIF)
19884909	BBA_01897	1.49	6.98E-06	2.48E-05	up	anthranilate synthase component II
19886303	BBA_03291	1.49	7.13E-36	1.44E-34	up	MFS monocarboxylate transporter
19892013	BBA_09001	1.49	5.75E-08	2.70E-07	up	prenyltransferase, UbiA family protein
19890951	BBA_07939	1.49	4.82E-19	5.04E-18	up	flavin-binding monooxygenase, putative
19888519	BBA_05507	1.49	2.53E-14	2.05E-13	up	hypothetical protein BBA_05507
19883734	BBA_00722	1.49	4.60E-12	3.14E-11	up	Cytochrome P450 CYP645A1
19886241	BBA_03229	1.49	5.61E-13	4.12E-12	up	hypothetical protein BBA_03229
19884293	BBA_01281	1.50	4.76E-29	7.55E-28	up	hypothetical protein BBA_01281
19884713	BBA_01701	1.50	2.98E-22	3.65E-21	up	C6 finger domain protein
19888780	BBA_05768	1.50	2.55E-25	3.60E-24	up	cysteine synthase
19888100	BBA_05088	1.50	1.87E-19	2.01E-18	up	cutinase-like protein
19892181	BBA_09169	1.50	1.32E-34	2.53E-33	up	Hexose transport-related protein, putative
19892918	BBA_09906	1.51	3.27E-28	5.09E-27	up	hypothetical protein BBA_09906
19889163	BBA_06151	1.51	2.43E-16	2.20E-15	up	ferric reductase like transmembrane component
19887601	BBA_04589	1.51	3.17E-13	2.36E-12	up	DUF866 domain-containing protein
19890133	BBA_07121	1.51	3.77E-10	2.23E-09	up	Pfs, NACHT and Ankyrin domain protein
19892972	BBA_09960	1.52	7.14E-19	7.39E-18	up	Rho-associated protein kinase, putative
19892610	BBA_09598	1.52	1.01E-11	6.80E-11	up	
19890791	BBA_07779	1.52	2.81E-14	2.27E-13	up	serine-threonine protein kinase
19890962	BBA_07950	1.52	1.64E-09	9.14E-09	up	hypothetical protein BBA_07950
19885303	BBA_02291	1.52	2.80E-07	1.21E-06	up	V-type ATPase
19892132	BBA_09120	1.52	2.42E-18	2.44E-17	up	hydrolase-like protein
19892445	BBA_09433	1.53	4.13E-38	8.86E-37	up	hypothetical protein BBA_09433
19884766	BBA_01754	1.53	2.08E-13	1.58E-12	up	DUF636 domain-containing protein
19893002	BBA_09990	1.54	7.98E-14	6.19E-13	up	dipeptidyl aminopeptidase/acylaminoacyl peptidase
19891742	BBA_08730	1.54	3.06E-30	5.10E-29	up	hypothetical protein BBA_08730
19891579	BBA_08567	1.54	4.57E-23	5.78E-22	up	methyltransferase domain-containing protein
19889064	BBA_06052	1.55	9.20E-16	8.08E-15	up	cyclase protein
19887975	BBA_04963	1.55	9.88E-09	5.08E-08	up	hypothetical protein BBA_04963
19885707	BBA_02695	1.55	1.71E-15	1.48E-14	up	short chain dehydrogenase
19888129	BBA_05117	1.55	2.66E-07	1.15E-06	up	SET domain-containing protein
19889411	BBA_06399	1.55	1.27E-20	1.45E-19	up	neutral/alkaline non-lysosomal ceramidase
19887040	BBA_04028	1.56	1.10E-19	1.19E-18	up	nonribosomal peptide synthetase 10
19892510	BBA_09498	1.56	5.59E-08	2.64E-07	up	FkbM family methyltransferase
19887183	BBA_04171	1.56	7.71E-35	1.50E-33	up	hypothetical protein BBA_04171
19889128	BBA_06116	1.57	4.76E-37	9.93E-36	up	CAP22 protein
19883068	BBA_00056	1.57	2.04E-06	7.88E-06	up	hypothetical protein BBA_00056
19891439	BBA_08427	1.57	1.45E-34	2.77E-33	up	fatty acid hydroxylase superfamily protein
19885670	BBA_02658	1.57	2.65E-11	1.73E-10	up	hypothetical protein BBA_02658
19890295	BBA_07283	1.58	1.80E-26	2.64E-25	up	
19885502	BBA_02490	1.58	1.15E-15	1.01E-14	up	FAD dependent oxidoreductase
19888260	BBA_05248	1.58	1.47E-15	1.28E-14	up	GPR1/FUN34/YaaH-class plasma membrane protein
19886063	BBA_03051	1.58	9.54E-17	8.85E-16	up	polyamine transporter 3
19892040	BBA_09028	1.59	3.71E-14	2.95E-13	up	Pfs, NACHT and Ankyrin domain protein
19887177	BBA_04165	1.59	7.95E-23	1.00E-21	up	toxD-like zinc binding oxidoreductase
19891421	BBA_08409	1.60	7.78E-07	3.18E-06	up	hypothetical protein BBA_08409
19883502	BBA_00490	1.60	3.92E-11	2.51E-10	up	DASH complex subunit Dad4

19890862	BBA_07850	1.60	1.04E-30	1.75E-29	up	hypothetical protein BBA_07850
19889372	BBA_06360	1.60	6.54E-12	4.43E-11	up	Succinyl-CoA synthetase, beta subunit
19891873	BBA_08861	1.60	1.55E-30	2.61E-29	up	hypothetical protein BBA_08861
19887860	BBA_04848	1.61	5.55E-27	8.26E-26	up	Alpha/beta hydrolase fold-3 domain protein
19886046	BBA_03034	1.61	1.82E-06	7.09E-06	up	hypothetical protein BBA_03034
19888028	BBA_05016	1.61	1.10E-03	2.67E-03	up	integral membrane protein pth11
19893020	BBA_10008	1.61	2.62E-12	1.81E-11	up	hypothetical protein BBA_10008
19887700	BBA_04688	1.62	3.84E-30	6.35E-29	up	glycosyl hydrolase family 76
19893229	BBA_10217	1.62	3.02E-34	5.67E-33	up	hypothetical protein BBA_10217
19885836	BBA_02824	1.62	1.21E-07	5.51E-07	up	short chain dehydrogenase
19892731	BBA_09719	1.62	1.25E-27	1.91E-26	up	MFS toxin efflux pump (AflT)
19889548	BBA_06536	1.63	4.03E-39	9.07E-38	up	carboxylic acid transport protein
19890946	BBA_07934	1.63	9.86E-16	8.64E-15	up	secretory lipase
19884343	BBA_01331	1.63	1.38E-26	2.04E-25	up	helix-turn-helix domain-containing protein
19889182	BBA_06170	1.64	9.80E-13	7.03E-12	up	Cry3Aa protein
19890397	BBA_07385	1.64	1.46E-38	3.19E-37	up	MFS transporter
19892002	BBA_08990	1.64	2.90E-35	5.74E-34	up	pyruvate decarboxylase
19884920	BBA_01908	1.64	3.52E-13	2.62E-12	up	hypothetical protein BBA_01908
19893336	BBA_10324	1.64	1.26E-09	7.11E-09	up	metalloreductase transmembrane component
19892723	BBA_09711	1.65	2.77E-37	5.81E-36	up	sphingolipid long chain base-responsive protein LSP1
19888305	BBA_05293	1.65	8.19E-03	1.64E-02	up	hypothetical protein BBA_05293
19883459	BBA_00447	1.65	7.76E-21	8.96E-20	up	
19883955	BBA_00943	1.65	6.31E-25	8.78E-24	up	hypothetical protein BBA_00943
19886021	BBA_03009	1.65	8.89E-35	1.73E-33	up	
19889229	BBA_06217	1.66	4.02E-19	4.23E-18	up	quinone oxidoreductase
19883105	BBA_00093	1.66	1.69E-09	9.43E-09	up	methyltransferase domain-containing protein
19886245	BBA_03233	1.66	3.29E-28	5.10E-27	up	GDP-mannose transporter
19892949	BBA_09937	1.66	6.31E-06	2.26E-05	up	hypothetical protein BBA_09937
19887620	BBA_04608	1.67	9.06E-10	5.16E-09	up	hypothetical protein BBA_04608
19892758	BBA_09746	1.67	1.64E-08	8.24E-08	up	major facilitator superfamily transporter
19891946	BBA_08934	1.67	1.93E-27	2.92E-26	up	proline dehydrogenase
19889019	BBA_06007	1.67	1.10E-27	1.68E-26	up	short-chain dehydrogenase
19886432	BBA_03420	1.67	1.30E-09	7.32E-09	up	hypothetical protein BBA_03420
19887068	BBA_04056	1.67	1.68E-16	1.54E-15	up	SpoVR like family protein
19890118	BBA_07106	1.68	6.53E-40	1.49E-38	up	CoA-transferase family III
19884859	BBA_01847	1.68	4.24E-19	4.46E-18	up	hypothetical protein BBA_01847
19889362	BBA_06350	1.68	3.98E-18	3.96E-17	up	C6 transcription factor
19886958	BBA_03946	1.68	6.09E-29	9.61E-28	up	glycosyl hydrolase family 2
19884115	BBA_01103	1.68	3.05E-16	2.74E-15	up	ABC transporter
19886594	BBA_03582	1.68	3.20E-41	7.52E-40	up	O-methyltransferase family protein
19892435	BBA_09423	1.69	7.75E-09	4.05E-08	up	translation factor (SUA5)
19886885	BBA_03873	1.69	1.22E-31	2.13E-30	up	calcium/proton exchanger
19884569	BBA_01557	1.69	1.33E-34	2.54E-33	up	choline-sulfatase-like protein
19886094	BBA_03082	1.69	1.88E-18	1.90E-17	up	beta (1-3) glucanosyltransferase
19887151	BBA_04139	1.70	8.08E-44	2.03E-42	up	glutamate 5-kinase
19891741	BBA_08729	1.70	8.89E-30	1.45E-28	up	phosphatidylserine decarboxylase family protein
19893146	BBA_10134	1.70	2.28E-44	5.83E-43	up	fatty acid hydroxylase superfamily protein
19893300	BBA_10288	1.70	9.48E-20	1.04E-18	up	hypothetical protein BBA_10288
19883457	BBA_00445	1.72	3.82E-26	5.53E-25	up	hypothetical protein BBA_00445
19886195	BBA_03183	1.73	2.08E-12	1.46E-11	up	hypothetical protein BBA_03183
19889388	BBA_06376	1.74	2.92E-41	6.92E-40	up	glycine-rich cell wall structural protein 1
19888319	BBA_05307	1.74	1.50E-17	1.45E-16	up	hypothetical protein BBA_05307
19885793	BBA_02781	1.75	1.96E-22	2.42E-21	up	WSC domain-containing protein
19888735	BBA_05723	1.75	2.85E-16	2.56E-15	up	fructosamine-3-kinase
19890660	BBA_07648	1.75	6.64E-44	1.67E-42	up	
19883649	BBA_00637	1.76	5.12E-36	1.04E-34	up	epoxide hydrolase
19890768	BBA_07756	1.76	2.90E-33	5.33E-32	up	prolyl oligopeptidase
19886473	BBA_03461	1.76	8.68E-31	1.47E-29	up	fatty acid desaturase
19887349	BBA_04337	1.77	2.53E-08	1.24E-07	up	hypothetical protein BBA_04337
19883584	BBA_00572	1.78	5.09E-19	5.31E-18	up	glyoxalase/bleomycin resistance protein/dioxygenase
19891445	BBA_08433	1.79	3.81E-09	2.06E-08	up	quercetin 2,3-dioxygenase

19886031	BBA_03019	1.79	2.73E-06	1.04E-05	up	hypothetical protein BBA_03019
19887335	BBA_04323	1.79	1.77E-46	4.70E-45	up	DUF1445 domain-containing protein
19890352	BBA_07340	1.80	7.47E-22	8.98E-21	up	monocarboxylate permease-like protein
19890293	BBA_07281	1.80	2.67E-17	2.54E-16	up	urea active transporter
19885974	BBA_02962	1.80	2.43E-16	2.20E-15	up	hypothetical protein BBA_02962
19884843	BBA_01831	1.80	1.12E-07	5.12E-07	up	hypothetical protein BBA_01831
19892086	BBA_09074	1.81	1.81E-26	2.65E-25	up	mitochondrial chaperone BCS1
19889423	BBA_06411	1.81	1.84E-41	4.40E-40	up	Gti1/Pac2 family protein
19891042	BBA_08030	1.81	3.27E-24	4.37E-23	up	hypothetical protein BBA_08030
19888768	BBA_05756	1.81	1.78E-37	3.78E-36	up	cell wall serine-threonine-rich galactomannoprotein Mp1
19891661	BBA_08649	1.81	1.53E-07	6.86E-07	up	hypothetical protein BBA_08649
19885562	BBA_02550	1.81	2.02E-24	2.74E-23	up	atmA protein
19889421	BBA_06409	1.82	3.45E-31	5.91E-30	up	flavin-containing amine oxidasedehydrogenase
19890609	BBA_07597	1.82	1.00E-38	2.21E-37	up	hypothetical protein BBA_07597
19891417	BBA_08405	1.82	2.76E-10	1.64E-09	up	hypothetical protein BBA_08405
19883806	BBA_00794	1.82	5.89E-21	6.84E-20	up	hypothetical protein BBA_00794
19885591	BBA_02579	1.83	1.02E-47	2.83E-46	up	beta-glucuronidase
19890508	BBA_07496	1.84	1.29E-09	7.26E-09	up	hypothetical protein BBA_07496
19892611	BBA_09599	1.84	5.73E-46	1.50E-44	up	
19883781	BBA_00769	1.84	4.14E-49	1.19E-47	up	hypothetical protein BBA_00769
19885056	BBA_02044	1.85	8.37E-41	1.95E-39	up	integral membrane protein, putative
19887275	BBA_04263	1.85	1.76E-44	4.52E-43	up	Bys1 family protein
19885052	BBA_02040	1.85	3.73E-07	1.59E-06	up	hypothetical protein BBA_02040
19887307	BBA_04295	1.85	1.15E-38	2.53E-37	up	C2 domain-containing protein
19883122	BBA_00110	1.86	1.13E-18	1.16E-17	up	hypothetical protein BBA_00110
19887280	BBA_04268	1.86	1.63E-10	9.88E-10	up	hypothetical protein BBA_04268
19889621	BBA_06609	1.86	3.20E-20	3.56E-19	up	myosin-cross-reactive antigen
19884917	BBA_01905	1.87	1.40E-25	1.99E-24	up	UPF0643 protein
19893225	BBA_10213	1.87	3.82E-20	4.25E-19	up	platelet-activating factor acetylhydrolase
19891773	BBA_08761	1.88	1.09E-08	5.58E-08	up	hypothetical protein BBA_08761
19890235	BBA_07223	1.88	7.02E-10	4.03E-09	up	hypothetical protein BBA_07223
19883559	BBA_00547	1.88	3.55E-15	3.03E-14	up	purine nucleoside permease
19883310	BBA_00298	1.88	7.12E-38	1.52E-36	up	putative U-snRNP-associated cyclophilin
19887045	BBA_04033	1.88	6.92E-26	9.91E-25	up	General substrate transporter
19889705	BBA_06693	1.90	9.92E-43	2.45E-41	up	hypothetical protein BBA_06693
19885901	BBA_02889	1.90	1.24E-34	2.39E-33	up	hypothetical protein BBA_02889
19891014	BBA_08002	1.90	1.94E-10	1.17E-09	up	L-PSP endoribonuclease family protein
19892876	BBA_09864	1.90	2.32E-41	5.51E-40	up	phenol acid carboxylase, putative
19893227	BBA_10215	1.90	1.15E-18	1.17E-17	up	hypothetical protein BBA_10215
19892512	BBA_09500	1.91	2.60E-08	1.28E-07	up	cuticle-degrading serine protease
19890510	BBA_07498	1.92	7.04E-09	3.69E-08	up	fatty acid-biding protein
19888890	BBA_05878	1.92	1.32E-19	1.43E-18	up	metal dependent phosphohydrolase
19892719	BBA_09707	1.93	2.16E-51	6.55E-50	up	hypothetical protein BBA_09707
19886959	BBA_03947	1.93	1.83E-24	2.50E-23	up	hypothetical protein BBA_03947
19890538	BBA_07526	1.94	4.09E-50	1.20E-48	up	protein rds1
19890008	BBA_06996	1.94	1.27E-17	1.23E-16	up	AMP-binding enzyme
19891837	BBA_08825	1.96	7.70E-30	1.26E-28	up	hypothetical protein BBA_08825
19888364	BBA_05352	1.96	6.37E-42	1.54E-40	up	hypothetical protein BBA_05352
19888417	BBA_05405	1.97	8.65E-13	6.25E-12	up	hypothetical protein BBA_05405
19888268	BBA_05256	1.98	1.23E-07	5.58E-07	up	nucleoside-diphosphate-sugar epimerase, putative
19883777	BBA_00765	1.99	8.75E-25	1.20E-23	up	NADPH oxidase
19891187	BBA_08175	2.00	1.55E-08	7.85E-08	up	evolved D-lactonohydrolase
19891189	BBA_08177	2.01	1.05E-20	1.20E-19	up	cation transporter
19889495	BBA_06483	2.01	8.09E-25	1.12E-23	up	Lactamase B
19886090	BBA_03078	2.02	7.31E-45	1.90E-43	up	Cytochrome P450 CYP68N1
19892499	BBA_09487	2.02	3.69E-31	6.32E-30	up	hypothetical protein BBA_09487
19887733	BBA_04721	2.03	4.43E-20	4.91E-19	up	serine protein kinase
19888178	BBA_05166	2.03	1.01E-23	1.32E-22	up	
19890132	BBA_07120	2.06	4.05E-45	1.06E-43	up	major facilitator superfamily transporter
19890021	BBA_07009	2.06	2.17E-47	5.95E-46	up	alkaline foam protein B precursor
19890795	BBA_07783	2.06	2.95E-18	2.97E-17	up	CFEM domain-containing protein

19889844	BBA_06832	2.06	4.59E-47	1.25E-45	up	fatty acid hydroxylase superfamily protein
19891776	BBA_08764	2.07	6.80E-37	1.41E-35	up	hypothetical protein BBA_08764
19887618	BBA_04606	2.07	6.52E-09	3.43E-08	up	hypothetical protein BBA_04606
19888701	BBA_05689	2.07	4.46E-23	5.66E-22	up	extracellular dioxygenase
19885131	BBA_02119	2.07	2.96E-25	4.15E-24	up	X-Pro dipeptidyl-peptidase (S15 family) protein
19889712	BBA_06700	2.08	3.57E-09	1.93E-08	up	alkaline serine protease AorO
19892054	BBA_09042	2.08	4.78E-47	1.30E-45	up	hypothetical protein BBA_09042
19889500	BBA_06488	2.09	1.07E-19	1.16E-18	up	hypothetical protein BBA_06488
19883297	BBA_00285	2.11	1.88E-17	1.80E-16	up	transmembrane protein
19891696	BBA_08684	2.12	9.75E-10	5.54E-09	up	fatty acid desaturase
19891970	BBA_08958	2.14	1.01E-35	2.02E-34	up	casein kinase 1 epsilon
19893160	BBA_10148	2.14	6.22E-26	8.93E-25	up	hypothetical protein BBA_10148
19886910	BBA_03898	2.15	3.32E-42	8.10E-41	up	small secreted protein
19889041	BBA_06029	2.16	3.06E-09	1.67E-08	up	carboxymuconolactone decarboxylase
19885673	BBA_02661	2.16	3.92E-17	3.70E-16	up	hypothetical protein BBA_02661
19885410	BBA_02398	2.16	1.31E-63	5.44E-62	up	aromatic and neutral aliphatic amino acid permease
19893127	BBA_10115	2.17	5.77E-20	6.32E-19	up	endo alpha-1,4 polygalactosaminidase precursor
19891675	BBA_08663	2.18	2.29E-21	2.70E-20	up	hypothetical protein BBA_08663
19885431	BBA_02419	2.18	5.54E-67	2.53E-65	up	adhesin protein Mad1
19892008	BBA_08996	2.18	3.53E-32	6.28E-31	up	NADH-cytochrome b5 reductase
19893155	BBA_10143	2.19	2.33E-15	2.00E-14	up	hypothetical protein BBA_10143
19885211	BBA_02199	2.19	9.91E-34	1.84E-32	up	neutral amino acid transporter
19892127	BBA_09115	2.21	1.43E-46	3.82E-45	up	Cupin family protein
19886590	BBA_03578	2.21	1.48E-56	5.16E-55	up	siderophore iron transporter mirB
19887202	BBA_04190	2.21	6.65E-06	2.37E-05	up	tat pathway signal sequence
19892450	BBA_09438	2.21	7.40E-09	3.87E-08	up	Methyltransferase type 11
19888835	BBA_05823	2.22	7.43E-14	5.78E-13	up	NAD-dependent epimerase/dehydratase
19885563	BBA_02551	2.22	1.16E-08	5.95E-08	up	hypothetical protein BBA_02551
19891273	BBA_08261	2.23	1.30E-31	2.28E-30	up	inositol oxygenase, putative
19883683	BBA_00671	2.23	7.14E-08	3.32E-07	up	PLAC8 family protein
19888160	BBA_05148	2.24	1.76E-27	2.67E-26	up	Acyl-CoA N-acyltransferase
19890880	BBA_07868	2.25	3.43E-65	1.49E-63	up	hypothetical protein BBA_07868
19892315	BBA_09303	2.25	5.85E-33	1.07E-31	up	hypothetical protein BBA_09303
19891440	BBA_08428	2.26	4.28E-37	8.94E-36	up	efflux pump antibiotic resistance protein
19888573	BBA_05561	2.26	1.30E-44	3.36E-43	up	RNA 3'-terminal phosphate cyclase
19890991	BBA_07979	2.27	3.47E-53	1.12E-51	up	hypothetical protein BBA_07979
19883109	BBA_00097	2.28	1.88E-38	4.07E-37	up	3-hydroxyacyl-CoA dehyrogenase
19890398	BBA_07386	2.28	1.44E-09	8.08E-09	up	hypothetical protein BBA_07386
19892637	BBA_09625	2.29	1.93E-21	2.28E-20	up	dsp1-1-like protein
19891272	BBA_08260	2.29	3.28E-09	1.78E-08	up	hypothetical protein BBA_08260
19886008	BBA_02996	2.31	5.88E-53	1.88E-51	up	antigenic cell wall galactomannoprotein, putative
19884918	BBA_01906	2.32	1.79E-15	1.54E-14	up	mitochondrial carrier protein (Pet8)
19889944	BBA_06932	2.32	7.83E-61	3.11E-59	up	Carbamoyl transferase
19886574	BBA_03562	2.32	3.30E-24	4.40E-23	up	hypothetical protein BBA_03562
19892443	BBA_09431	2.32	4.67E-22	5.64E-21	up	hypothetical protein BBA_09431
19890384	BBA_07372	2.33	4.32E-13	3.20E-12	up	homogentisate 1,2-dioxygenase
19883458	BBA_00446	2.33	2.97E-35	5.86E-34	up	oxidoreductase-like protein
19892605	BBA_09593	2.33	3.04E-65	1.32E-63	up	adenosine deaminase
19888122	BBA_05110	2.35	2.25E-13	1.70E-12	up	phloretin hydrolase
19884548	BBA_01536	2.35	1.33E-14	1.10E-13	up	hypothetical protein BBA_01536
19890020	BBA_07008	2.36	2.73E-72	1.42E-70	up	2,3-diketo-5-methylthio-1-phosphopentane phosphatase
19888588	BBA_05576	2.39	3.76E-32	6.68E-31	up	hypothetical protein BBA_05576
19892225	BBA_09213	2.40	2.35E-17	2.25E-16	up	hypothetical protein BBA_09213
19889344	BBA_06332	2.41	9.19E-76	5.11E-74	up	gamma-glutamyltranspeptidase
19891593	BBA_08581	2.41	2.04E-08	1.01E-07	up	hypothetical protein BBA_08581
19885776	BBA_02764	2.41	7.11E-41	1.66E-39	up	phenazine biosynthesis-like protein
19887101	BBA_04089	2.42	2.29E-50	6.78E-49	up	zinc-binding dehydrogenase
19889697	BBA_06685	2.44	8.54E-84	5.89E-82	up	hypothetical protein BBA_06685
19892915	BBA_09903	2.45	4.70E-16	4.18E-15	up	hypothetical protein BBA_09903
19893159	BBA_10147	2.46	2.26E-24	3.04E-23	up	hypothetical protein BBA_10147
19888451	BBA_05439	2.46	9.49E-68	4.40E-66	up	Cytochrome P450 CYP539B1

19892446	BBA_09434	2.48	2.30E-19	2.45E-18	up	hypothetical protein BBA_09434
19887133	BBA_04121	2.49	6.69E-13	4.88E-12	up	ethanolamine utilization protein (EutQ)
19890100	BBA_07088	2.49	2.82E-32	5.05E-31	up	xylose isomerase-like TIM barrel
19892213	BBA_09201	2.49	3.12E-28	4.88E-27	up	hypothetical protein BBA_09201
19883570	BBA_00558	2.50	1.02E-12	7.28E-12	up	hypothetical protein BBA_00558
19889178	BBA_06166	2.51	1.48E-75	8.14E-74	up	alcohol dehydrogenase I
19883819	BBA_00807	2.51	2.95E-48	8.36E-47	up	glucose repressible protein Grg1
19890726	BBA_07714	2.52	1.29E-26	1.90E-25	up	hypothetical protein BBA_07714
19885593	BBA_02581	2.52	9.39E-61	3.69E-59	up	fatty acid hydroxylase superfamily protein
19887264	BBA_04252	2.53	1.41E-56	4.94E-55	up	F1F0-ATP synthase regulatory factor Stf2
19886506	BBA_03494	2.55	1.38E-35	2.75E-34	up	hypothetical protein BBA_03494
19891875	BBA_08863	2.58	6.69E-15	5.60E-14	up	metalloprotease 1
19891963	BBA_08951	2.59	9.92E-22	1.18E-20	up	hypothetical protein BBA_08951
19891701	BBA_08689	2.60	1.39E-14	1.15E-13	up	Putative GMC oxidoreductase
19887340	BBA_04328	2.62	4.62E-26	6.67E-25	up	aminotriazole resistance protein
19888658	BBA_05646	2.63	4.83E-28	7.47E-27	up	autophagy protein (Atg22), putative
19887681	BBA_04669	2.63	9.33E-23	1.17E-21	up	3-octaprenyl-4-hydroxybenzoate carboxy-lyase, putative
19888024	BBA_05012	2.63	1.81E-18	1.84E-17	up	Carbohydrate kinase, thermoresistant glucokinase
19893278	BBA_10266	2.66	6.55E-32	1.15E-30	up	sugar transporter
19892121	BBA_09109	2.67	2.93E-40	6.77E-39	up	Catalase-like domain, heme-dependent
19889850	BBA_06838	2.68	9.27E-40	2.11E-38	up	phospholipase/carboxylesterase
19892243	BBA_09231	2.69	6.79E-32	1.19E-30	up	hypothetical protein BBA_09231
19891978	BBA_08966	2.70	4.27E-35	8.39E-34	up	hypothetical protein BBA_08966
19892055	BBA_09043	2.72	4.35E-39	9.75E-38	up	mitogen-activated protein kinase sty1
19892448	BBA_09436	2.72	3.08E-48	8.67E-47	up	zinc finger-like protein
19891824	BBA_08812	2.72	1.65E-95	1.51E-93	up	secreted lipase 1 precursor
19890645	BBA_07633	2.72	3.78E-16	3.37E-15	up	hypothetical protein BBA_07633
19892004	BBA_08992	2.73	3.10E-31	5.36E-30	up	branched-chain-amino-acid aminotransferase
19887697	BBA_04685	2.74	1.08E-99	1.07E-97	up	2OG-Fe(II) oxygenase superfamily protein
19885377	BBA_02365	2.75	1.82E-60	7.08E-59	up	hypothetical protein BBA_02365
19890635	BBA_07623	2.75	1.72E-81	1.09E-79	up	trehalase-like protein
19887710	BBA_04698	2.77	1.02E-63	4.30E-62	up	hypothetical protein BBA_04698
19883484	BBA_00472	2.78	1.20E-97	1.16E-95	up	3-dehydroquinate synthase
19884975	BBA_01963	2.79	2.00E-36	4.11E-35	up	pepsin A1
19883482	BBA_00470	2.79	6.64E-83	4.45E-81	up	ATP-grasp enzyme-like protein
19891735	BBA_08723	2.82	5.71E-25	7.98E-24	up	Epl1 protein
19886929	BBA_03917	2.84	2.00E-26	2.93E-25	up	hypothetical protein BBA_03917
19885620	BBA_02608	2.85	1.34E-14	1.11E-13	up	biotrophy-associated secreted protein 2
19885023	BBA_02011	2.85	2.42E-14	1.96E-13	up	zinc finger protein Nv-ZicA
19888691	BBA_05679	2.90	1.14E-37	2.42E-36	up	HHE domain-containing protein
19892292	BBA_09280	2.90	1.07E-19	1.16E-18	up	hypothetical protein BBA_09280
19886962	BBA_03950	2.91	3.07E-23	3.93E-22	up	Beta-lactamase-type transpeptidase
19887509	BBA_04497	2.91	1.86E-13	1.41E-12	up	retrotransposon protein, putative, Ty1-copia subclass
19890004	BBA_06992	2.92	9.35E-88	7.36E-86	up	short chain dehydrogenase
19884549	BBA_01537	2.93	2.62E-56	9.04E-55	up	hypothetical protein BBA_01537
19888277	BBA_05265	2.98	1.07E-95	1.00E-93	up	hypothetical protein BBA_05265
19889198	BBA_06186	2.98	4.26E-97	4.07E-95	up	Catalase-like domain, heme-dependent
19892478	BBA_09466	2.99	2.64E-22	3.24E-21	up	hypothetical protein BBA_09466
19887922	BBA_04910	2.99	5.01E-40	1.15E-38	up	hypothetical protein BBA_04910
19883073	BBA_00061	3.00	2.27E-34	4.30E-33	up	
19890003	BBA_06991	3.00	2.61E-98	2.56E-96	up	
19888448	BBA_05436	3.00	4.09E-67	1.89E-65	up	hypothetical protein BBA_05436
19884038	BBA_01026	3.01	1.09E-120	1.41E-118	up	hypothetical protein BBA_01026
19893156	BBA_10144	3.02	8.79E-43	2.18E-41	up	Methyltransferase type 11
19883762	BBA_00750	3.03	2.61E-59	9.76E-58	up	hypothetical protein BBA_00750
19888976	BBA_05964	3.03	6.52E-32	1.15E-30	up	hypothetical protein BBA_05964
19893015	BBA_10003	3.03	6.26E-39	1.40E-37	up	mitochondrial chaperone BCS1
19887485	BBA_04473	3.04	2.57E-46	6.81E-45	up	hypothetical protein BBA_04473
19887000	BBA_03988	3.05	3.68E-62	1.49E-60	up	hypothetical protein BBA_03988
19888666	BBA_05654	3.05	2.75E-86	2.02E-84	up	OsmC-like protein
19892449	BBA_09437	3.06	3.29E-31	5.65E-30	up	C2H2 finger domain protein, putative

19886948	BBA_03936	3.06	2.83E-37	5.93E-36	up	DUF1264 domain-containing protein
19892186	BBA_09174	3.07	1.32E-63	5.45E-62	up	cell surface protein
19889044	BBA_06032	3.09	1.04E-54	3.50E-53	up	spherulin-1A
19891677	BBA_08665	3.11	1.36E-11	9.02E-11	up	trypsin-like protease
19890418	BBA_07406	3.12	3.48E-09	1.89E-08	up	acyl-CoA thioesterase II
19890850	BBA_07838	3.12	8.47E-75	4.57E-73	up	hypothetical protein BBA_07838
19888332	BBA_05320	3.12	1.50E-82	9.95E-81	up	plasma membrane zinc ion transporter, putative
19892007	BBA_08995	3.14	4.25E-84	2.95E-82	up	hypothetical protein BBA_08995
19884472	BBA_01460	3.15	1.64E-68	7.87E-67	up	hypothetical protein BBA_01460
19886224	BBA_03212	3.17	7.09E-121	9.30E-119	up	phthalate transporter
19892214	BBA_09202	3.19	4.65E-111	5.05E-109	up	hypothetical protein BBA_09202
19885787	BBA_02775	3.20	4.26E-89	3.47E-87	up	Amine oxidase
19889468	BBA_06456	3.20	6.02E-26	8.65E-25	up	HAD-superfamily hydrolase, subfamily IA, variant 1
19886261	BBA_03249	3.20	2.69E-24	3.61E-23	up	hypothetical protein BBA_03249
19887619	BBA_04607	3.21	3.20E-43	8.00E-42	up	hypothetical protein BBA_04607
19883880	BBA_00868	3.22	5.16E-52	1.58E-50	up	G-protein coupled receptor
19892640	BBA_09628	3.23	4.68E-22	5.65E-21	up	C6 zinc finger domain-containing protein
19892185	BBA_09173	3.23	8.79E-28	1.35E-26	up	hypothetical protein BBA_09173
19885133	BBA_02121	3.23	2.42E-20	2.72E-19	up	hypothetical protein BBA_02121
19891451	BBA_08439	3.23	2.63E-98	2.56E-96	up	zinc-binding dehydrogenase
19888779	BBA_05767	3.24	1.64E-128	2.62E-126	up	tetracycline efflux protein (otrb)
19891997	BBA_08985	3.25	2.29E-21	2.70E-20	up	ThiJ/PfpI family protein
19885944	BBA_02932	3.25	4.57E-60	1.76E-58	up	General substrate transporter
19892313	BBA_09301	3.28	1.01E-12	7.23E-12	up	amino acid transporter
19886964	BBA_03952	3.29	2.45E-26	3.58E-25	up	glutathione-dependent formaldehyde-activating, GFA
19889285	BBA_06273	3.32	7.59E-75	4.12E-73	up	OPT oligopeptide transporter
19892281	BBA_09269	3.33	1.62E-18	1.65E-17	up	secreted lipase 1 precursor
19888276	BBA_05264	3.33	3.62E-58	1.31E-56	up	chitinase-like protein
19891517	BBA_08505	3.34	3.70E-65	1.60E-63	up	subtilase-like protein
19890794	BBA_07782	3.36	1.20E-58	4.39E-57	up	hypothetical protein BBA_07782
19892971	BBA_09959	3.39	1.96E-135	3.49E-133	up	hypothetical protein BBA_09959
19887698	BBA_04686	3.40	2.75E-52	8.49E-51	up	pantothenate transporter
19892966	BBA_09954	3.42	2.30E-124	3.45E-122	up	C2H2 finger domain protein, putative
19892183	BBA_09171	3.42	1.85E-71	9.45E-70	up	hypothetical protein BBA_09171
19885592	BBA_02580	3.48	1.88E-121	2.53E-119	up	allergen-like protein
19891544	BBA_08532	3.51	3.86E-42	9.41E-41	up	hypothetical protein BBA_08532
19888690	BBA_05678	3.54	1.16E-60	4.54E-59	up	nitrogen assimilation transcription factor nirA
19889043	BBA_06031	3.56	8.47E-21	9.73E-20	up	metallo-beta-lactamase superfamily protein
19890102	BBA_07090	3.56	4.21E-29	6.71E-28	up	hypothetical protein BBA_07090
19892180	BBA_09168	3.57	5.42E-87	4.13E-85	up	retinol dehydrogenase 12
19893010	BBA_09998	3.57	3.58E-91	3.02E-89	up	conidial wall protein
19884850	BBA_01838	3.58	6.28E-60	2.40E-58	up	hypothetical protein BBA_01838
19887734	BBA_04722	3.59	8.85E-26	1.26E-24	up	hypothetical protein BBA_04722
19884627	BBA_01615	3.60	9.22E-147	1.89E-144	up	aquaporin-2
19885395	BBA_02383	3.61	6.13E-74	3.25E-72	up	hypothetical protein BBA_02383
19892074	BBA_09062	3.63	1.09E-38	2.39E-37	up	hypothetical protein BBA_09062
19887720	BBA_04708	3.65	1.23E-61	4.90E-60	up	hypothetical protein BBA_04708
19891769	BBA_08757	3.66	4.58E-31	7.82E-30	up	FAD binding domain-containing protein
19890509	BBA_07497	3.70	2.26E-84	1.58E-82	up	hypothetical protein BBA_07497
19887735	BBA_04723	3.72	4.54E-20	5.01E-19	up	hypothetical protein BBA_04723
19891979	BBA_08967	3.73	1.46E-94	1.28E-92	up	fungal zinc cluster transcription factor
19885778	BBA_02766	3.74	2.34E-50	6.90E-49	up	BTB domain transcription factor
19884290	BBA_01278	3.75	2.08E-85	1.49E-83	up	pyridoxamine 5'-phosphate oxidase
19885581	BBA_02569	3.77	9.87E-82	6.34E-80	up	hypothetical protein BBA_02569
19887100	BBA_04088	3.78	3.77E-55	1.28E-53	up	hypothetical protein BBA_04088
19890354	BBA_07342	3.79	1.45E-20	1.65E-19	up	C6 transcription factor
19887116	BBA_04104	3.79	1.47E-100	1.48E-98	up	DNA-binding protein
19889459	BBA_06447	3.83	8.90E-53	2.78E-51	up	hypothetical protein BBA_06447
19893246	BBA_10234	3.85	7.60E-40	1.73E-38	up	tetratricopeptide repeat domain protein
19888767	BBA_05755	3.86	2.34E-81	1.46E-79	up	hypothetical protein BBA_05755
19889199	BBA_06187	3.87	1.11E-77	6.45E-76	up	hypothetical protein BBA_06187

19883859	BBA_00847	3.88	4.84E-116	5.72E-114	up	alkaline phosphatase
19885739	BBA_02727	3.91	3.52E-33	6.44E-32	up	Chymotrypsin-like protein
19883811	BBA_00799	3.92	2.80E-172	7.77E-170	up	phosphate transporter
19892052	BBA_09040	4.01	3.15E-33	5.78E-32	up	hypothetical protein BBA_09040
19890981	BBA_07969	4.05	6.92E-30	1.14E-28	up	cutinase precursor
19891770	BBA_08758	4.06	2.52E-70	1.25E-68	up	FAD dependent oxidoreductase
19885109	BBA_02097	4.08	1.71E-52	5.31E-51	up	gll2891-like protein
19893189	BBA_10177	4.10	3.03E-18	3.04E-17	up	phosphatidylserine decarboxylase
19889020	BBA_06008	4.19	8.48E-53	2.66E-51	up	FAD binding domain-containing protein
19886947	BBA_03935	4.19	1.59E-88	1.27E-86	up	hypothetical protein BBA_03935
19891502	BBA_08490	4.25	7.13E-13	5.19E-12	up	hypothetical protein BBA_08490
19892038	BBA_09026	4.28	9.84E-76	5.43E-74	up	isochorismatase family protein
19892609	BBA_09597	4.36	1.41E-123	1.99E-121	up	
19890335	BBA_07323	4.39	3.22E-80	1.99E-78	up	6-phosphogluconate dehydrogenase
19887102	BBA_04090	4.39	1.26E-127	1.98E-125	up	short-chain dehydrogenase
19893228	BBA_10216	4.40	1.96E-52	6.09E-51	up	hypothetical protein BBA_10216
19885466	BBA_02454	4.45	3.37E-130	5.59E-128	up	hypothetical protein BBA_02454
19891503	BBA_08491	4.51	2.07E-34	3.94E-33	up	GEgh16 protein
19892945	BBA_09933	4.51	7.51E-79	4.49E-77	up	hypothetical protein BBA_09933
19883069	BBA_00057	4.55	2.74E-17	2.61E-16	up	hypothetical protein BBA_00057
19889062	BBA_06050	4.62	4.64E-83	3.13E-81	up	hypothetical protein BBA_06050
19892608	BBA_09596	4.66	4.87E-64	2.06E-62	up	succinate dehydrogenase cytochrome b subunit
19892244	BBA_09232	4.69	1.60E-54	5.31E-53	up	hypothetical protein BBA_09232
19887719	BBA_04707	4.71	1.63E-28	2.55E-27	up	hypothetical protein BBA_04707
19883636	BBA_00624	4.72	6.78E-14	5.29E-13	up	hypothetical protein BBA_00624
19893267	BBA_10255	4.74	7.21E-118	8.96E-116	up	Methyltransferase type 11
19891504	BBA_08492	4.94	3.20E-36	6.53E-35	up	extracellular serine-rich protein
19885904	BBA_02892	5.05	6.65E-255	5.71E-252	up	hypothetical protein BBA_02892
19886256	BBA_03244	5.34	4.98E-167	1.31E-164	up	HLH transcription factor
19892447	BBA_09435	5.36	3.11E-193	1.22E-190	up	C6 zinc finger domain-containing protein
19885614	BBA_02602	5.51	1.92E-238	1.40E-235	up	cell wall protein
19892970	BBA_09958	5.56	2.83E-158	6.67E-156	up	hypothetical protein BBA_09958
19893296	BBA_10284	5.56	1.17E-47	3.21E-46	up	mitochondrial chaperone bcs1
19892515	BBA_09503	5.91	7.38E-118	9.05E-116	up	WD domain protein
19893148	BBA_10136	5.94	3.91E-19	4.12E-18	up	Ferritin/ribonucleotide reductase
19892269	BBA_09257	6.20	2.34E-86	1.72E-84	up	cis,cis-muconate lactonizing enzyme precursor
19886507	BBA_03495	6.34	3.79E-154	8.33E-152	up	fatty acid hydroxylase superfamily protein
19893266	BBA_10254	6.88	7.28E-49	2.09E-47	up	hypothetical protein BBA_10254
19886961	BBA_03949	6.88	4.16E-274	3.93E-271	up	hydroxyacylglutathione hydrolase
19889508	BBA_06496	7.09	2.33E-48	6.65E-47	up	penicillin-binding protein
19892967	BBA_09955	7.12	3.11E-205	1.33E-202	up	dsp1-1-like protein
19892916	BBA_09904	7.14	1.01E-245	7.98E-243	up	hypothetical protein BBA_09904
19892767	BBA_09755	7.32	2.24E-58	8.17E-57	up	epoxide hydrolase
19891678	BBA_08666	7.38	3.02E-150	6.33E-148	up	bacteriodes thetaiotaomicron symbiotic chitinase
19886504	BBA_03492	7.47	0.00E+00	0.00E+00	up	hypothetical protein BBA_03492
19883051	BBA_00039	7.54	4.37E-72	2.25E-70	up	hypothetical protein BBA_00039
19892917	BBA_09905	7.79	1.04E-141	2.01E-139	up	hypothetical protein BBA_09905
19893147	BBA_10135	8.50	5.97E-65	2.55E-63	up	hypothetical protein BBA_10135
19887629	BBA_04617	9.13	5.15E-97	4.86E-95	up	subtilisin-like protease Pr1A
19886257	BBA_03245	9.64	5.33E-91	4.45E-89	up	cell wall protein
19886505	BBA_03493	11.36	0.00E+00	0.00E+00	up	cell wall protein
19886259	BBA_03247	11.81	2.47E-113	2.77E-111	up	cell wall protein
19886260	BBA_03248	12.05	0.00E+00	0.00E+00	up	major facilitator superfamily transporter
19886258	BBA_03246	12.08	0.00E+00	0.00E+00	up	cell wall protein
19887959	BBA_04947	15.32	9.03E-78	5.30E-76	up	Cytochrome P450 CYP617A1
19887958	BBA_04946	15.71	4.39E-86	3.17E-84	up	flavin-binding monooxygenase, putative
19890612	BBA_07600	15.84	4.53E-44	1.14E-42	up	hypothetical protein BBA_07600

Table S4. Counts of Δ*cre1*-specific dysregulated genes enriched to GO terms in three function classes of *B. bassiana* at the significant level of $p < 0.05$.

Enriched GO terms	Counts of enriched DEGs		
	Down	Up	Subtotal
**Function Class: Cellular Component**			
cellular component	455	303	758
integral component of membrane	52	40	92
extracellular region	28	9	37
RISC complex	4	0	4
membrane raft	2	2	4
**Total**	**541**	**354**	**895**
**Function Class: Biological Process**			
biological_process	268	183	451
oxidation-reduction process	64	42	106
transmembrane transport	48	38	86
metabolic process	20	25	45
proteolysis	26	4	30
methylation	8	5	13
nucleoside metabolic process	10	2	12
fatty acid biosynthetic process	2	8	10
protein catabolic process	6	2	8
glucose import	4	4	8
steroid metabolic process	3	3	6
nonribosomal peptide biosynthetic process	5	0	5
drug transmembrane transport	4	1	5
lipid catabolic process	2	3	5
fatty acid metabolic process	1	4	5
proteolysis involved in cellular protein catabolic process	5	0	5
cadmium ion transport	2	2	4
fatty acid catabolic process	4	0	4
regulation of small GTPase mediated signal transduction	4	0	4
cellular carbohydrate catabolic process	3	1	4
response to oxidative stress	4	0	4
phospholipid biosynthetic process	2	2	4
oxalate metabolic process	2	1	3
cellular aromatic compound metabolic process	2	1	3
methylglyoxal catabolic process to D-lactate via S-lactoyl-glutathione	0	3	3
regulation of filamentous growth of a population of unicellular organisms in response to biotic stimulus	3	0	3
carotene biosynthetic process	2	0	2
cinnamic acid catabolic process	0	2	2
conversion of ds siRNA to ss siRNA involved in RNA interference	2	0	2
D-gluconate metabolic process	2	0	2
emericellamide A biosynthetic process	2	0	2
ergot alkaloid biosynthetic process	2	0	2
glycoside catabolic process	2	0	2
oxylipin biosynthetic process	2	0	2
aromatic compound catabolic process	2	0	2
polysaccharide catabolic process	2	0	2
positive regulation of telomere maintenance via telomerase	1	1	2
purine nucleobase catabolic process	2	0	2
triglyceride catabolic process	0	2	2
**Total**	**523**	**339**	**862**

**Function Class: Molecular Function**			
molecular_function	255	200	455
oxidoreductase activity	38	35	73
catalytic activity	38	22	60
iron ion binding	26	12	38
heme binding	26	7	33
oxidoreductase activity, acting on paired donors, with incorporation or reduction of molecular oxygen	26	6	32
electron carrier activity	22	6	28
monooxygenase activity	13	6	19
nucleoside-triphosphatase activity	13	6	19
flavin adenine dinucleotide binding	11	7	18
transmembrane transporter activity	9	7	16
S-adenosylmethionine-dependent methyltransferase activity	8	7	15
lipase/triglyceride lipase activity	9	5	14
sequence-specific DNA binding	11	3	14
serine-type endopeptidase activity	9	4	13
aspartic-type endopeptidase activity	8	2	10
phosphopantetheine binding	8	0	8
glucose transmembrane transporter activity	4	4	8
substrate-specific transmembrane transporter activity	5	3	8
oxidoreductase activity, acting on CH-OH group of donors	3	4	7
acid phosphatase activity	6	0	6
coenzyme binding	3	3	6
serine-type carboxypeptidase activity	6	0	6
FMN binding	3	3	6
carboxy-lyase activity	0	5	5
O-methyltransferase activity	2	3	5
guanyl-nucleotide exchange factor activity	5	0	5
cation-transporting ATPase activity	5	0	5
protein dimerization activity	3	2	5
ADP binding	4	0	4
serine-type peptidase activity	4	0	4
oligopeptide transmembrane transporter activity	4	0	4
peroxidase activity	3	0	3
ammonium transmembrane transporter activity	2	1	3
beta-N-acetylhexosaminidase activity	3	0	3
carbon-sulfur lyase activity	0	3	3
serine-type peptidase activity	0	2	2
cadmium-exporting ATPase activity	1	1	2
fatty acid binding	0	2	2
glutamate 5-kinase activity	1	1	2
hydroxymethyl-, formyl- and related transferase activity	0	2	2
malate dehydrogenase (decarboxylating) (NAD+) activity	1	1	2
siRNA binding	2	0	2
chitinase activity	7	0	2
**Total**	**607**	**375**	**977**

Table S5. Counts of Δ*cre1*-specific dysregulated genes enriched to the KEGG pathways of *B. bassiana* at the significant level of $p < 0.05$.

**Pathway_ID**	**KEGG_pathway_name**	**Counts of enriched DEGs**		
		**Down**	**Up**	**Subtotal**
ko01200	Carbon metabolism	19	19	38
ko00380	Tryptophan metabolism	21	10	31
ko00350	Tyrosine metabolism	15	14	29
ko00071	Fatty acid degradation	17	10	27
ko00260	Glycine, serine and threonine metabolism	20	7	27
ko04146	Peroxisome	14	10	24
ko00561	Glycerolipid metabolism	18	5	23
ko00010	Glycolysis / Gluconeogenesis	11	10	21
ko02010	ABC transporters	13	5	18
ko00620	Pyruvate metabolism	12	4	16
ko00310	Lysine degradation	11	5	16
ko01212	Fatty acid metabolism	5	11	16
ko00404	Staurosporine biosynthesis	10	5	15
ko00061	Fatty acid biosynthesis	3	9	12
ko00640	Propanoate metabolism	7	5	12
ko00630	Glyoxylate and dicarboxylate metabolism	7	5	12
ko00600	Sphingolipid metabolism	7	4	11
ko00030	Pentose phosphate pathway	5	5	10
ko00780	Biotin metabolism	3	7	10
ko00052	Galactose metabolism	8	1	9
ko00511	Other glycan degradation	5	2	7
ko00603	Glycosphingolipid biosynthesis - globo series	5	0	5
	**Total**	**236**	**153**	**389**

Table S6. Lists of Δ*cre1*-specific dysregulated genes associated with phenotypic changes and involved in crucial cellular processes and events of *B. bassiana*.

Gene_ID	Genomic tag_locus	Δ*cre1* vs. WT			Annotation
		$\log_2 R$	*q* value	Regulation	
**Involved in normal cuticle infection and virulence-related cellular events**					
19885642	BBA_02630	-12.23	6.19E-75	down	bassianolide nonribosomal peptide synthetase
19891803	BBA_08791	-8.16	0.00E+00	down	alkaline serine protease AorO
19892188	BBA_09176	-8.01	9.85E-217	down	alkaline serine protease AorO
19886794	BBA_03782	-5.28	3.91E-164	down	putative hydrolase
19885641	BBA_02629	-4.42	6.22E-26	down	Putative conidiospore surface protein
19888365	BBA_05353	-3.75	1.28E-14	down	class III chitinase ChiA2
19883647	BBA_00635	-3.45	1.58E-09	down	Secretory lipase family protein
19886826	BBA_03814	-3.41	4.55E-61	down	chitinase-like protein
19887283	BBA_04271	-3.08	2.02E-77	down	pheromone receptor
19891433	BBA_08421	-2.77	2.08E-03	down	volvatoxin A2 precursor
19886459	BBA_03447	-2.70	5.36E-33	down	extracellular protein
19886921	BBA_03909	-2.62	5.14E-76	down	filamentous hemagglutinin / adhesin
19887096	BBA_04084	-2.45	1.90E-57	down	putative aspartic protease
19885429	BBA_02417	-2.44	2.01E-46	down	secreted aspartic proteinase
19889309	BBA_06297	-2.42	2.57E-37	down	chitinase-like protein
19892301	BBA_09289	-2.39	2.43E-51	down	Pectin lyase fold/virulence factor
19888837	BBA_05825	-2.17	1.45E-25	down	secreted glucosidase
19892165	BBA_09153	-2.15	1.35E-55	down	subtilisin-like serine protease Pr1C
19889439	BBA_06427	-1.62	6.82E-29	down	glycoside hydrolase family 13
19884015	BBA_01003	-1.49	1.28E-11	down	subtilase-like protein
19883455	BBA_00443	-1.48	6.63E-15	down	subtilisin-like protease Pr1B2
19885980	BBA_02968	-1.44	1.09E-10	down	regulator of G protein signaling
19891254	BBA_08242	-1.41	1.78E-07	down	glycoside hydrolase family 43 protein
19885226	BBA_02214	-1.30	2.04E-10	down	subtilase-like protein
19885242	BBA_02230	-1.29	2.13E-16	down	chitinase-like protein
19885444	BBA_02432	-1.28	3.29E-14	down	triacylglycerol lipase
19892617	BBA_09605	-1.27	1.93E-03	down	chitinase-like protein
19890643	BBA_07631	-1.22	7.98E-18	down	family S53 protease
19890911	BBA_07899	-1.12	2.93E-15	down	chitinase 18-3
19891435	BBA_08423	-1.01	3.88E-05	down	lipase-like protein
19883159	BBA_00147	1.19	1.71E-10	up	cutinase-2 protein
19888282	BBA_05270	1.22	2.16E-10	up	lipase class 2
19888100	BBA_05088	1.50	2.01E-18	up	cutinase-like protein
19892132	BBA_09120	1.52	2.44E-17	up	hydrolase-like protein
19889182	BBA_06170	1.64	7.03E-12	up	Cry3Aa protein
19892512	BBA_09500	1.91	1.28E-07	up	cuticle-degrading serine protease
19889712	BBA_06700	2.08	1.93E-08	up	alkaline serine protease AorO
19885431	BBA_02419	2.18	2.53E-65	up	adhesin protein Mad1
19891875	BBA_08863	2.58	5.60E-14	up	metalloprotease 1
19891677	BBA_08665	3.11	9.02E-11	up	trypsin-like protease
19883880	BBA_00868	3.22	1.58E-50	up	G-protein coupled receptor
19888276	BBA_05264	3.33	1.31E-56	up	chitinase-like protein
19891517	BBA_08505	3.34	1.60E-63	up	subtilase-like protein

19890981	BBA_07969	4.05	1.14E-28	up	cutinase precursor
19887629	BBA_04617	9.13	4.86E-95	up	subtilisin-like protease Pr1B3
**Involved in asexual development (conidiation)**					
19885168	BBA_02156	-2.85	5.77E-85	down	sporulation associated protein
19886838	BBA_03826	-1.95	6.78E-47	down	sporulation associated protein
19884540	BBA_01528	-1.74	1.26E-25	down	frequency clock protein
19885888	BBA_02876	-1.73	4.55E-30	down	vivid PAS protein VVD
19883724	BBA_00712	-1.25	1.88E-11	down	putative C6-zinc finger TF, regulator of conidiation
19885439	BBA_02427	1.10	6.03E-07	up	conidiation-specific protein (con-13)
**Involved in antioxdant response**					
19886483	BBA_03471	-5.10	1.07E-39	down	FAD dependent oxidoreductase domain containing protein
19892311	BBA_09299	-4.75	7.77E-59	down	sarcosine oxidase
19884826	BBA_01814	-4.52	3.49E-154	down	oxidoreductase, 2OG-Fe(II) oxygenase family
19890756	BBA_07744	-4.08	5.14E-71	down	FAD dependent oxidoreductase
19891755	BBA_08743	-3.39	3.34E-125	down	sarcosine oxidase
19883676	BBA_00664	-2.12	8.84E-22	down	oxidoreductase, short-chain dehydrogenase/reductase family
19893021	BBA_10009	-2.10	3.88E-18	down	6-hydroxy-D-nicotine oxidase
19888615	BBA_05603	-1.99	8.55E-47	down	Catalase-like domain, heme-dependent
19892783	BBA_09771	-1.96	4.11E-36	down	FAD dependent oxidoreductase
19892693	BBA_09681	-1.82	3.44E-37	down	sarcosine oxidase
19887242	BBA_04230	-1.70	9.20E-32	down	oxidoreductase family protein
19889838	BBA_06826	-1.54	2.28E-07	down	glucose oxidase precursor
19885596	BBA_02584	-1.54	2.83E-29	down	oxidoreductase family protein
19886833	BBA_03821	-1.46	1.27E-12	down	N,N-dimethylglycine oxidase
19890309	BBA_07297	-1.28	8.62E-13	down	oxidoreductase, short-chain dehydrogenase/reductase
19886832	BBA_03820	-1.20	2.79E-18	down	sarcosine oxidase
19887037	BBA_04025	-1.18	1.17E-07	down	glucose oxidase
19884227	BBA_01215	-1.16	3.61E-05	down	oxidoreductase, short chain dehydrogenase/reductase family superfamily
19889492	BBA_06480	-1.13	1.42E-10	down	oxidoreductase FAD-binding domain-containing protein
19890790	BBA_07778	-1.10	1.73E-12	down	oxidoreductase, 2OG-Fe(II) oxygenase family protein
19888833	BBA_05821	-1.04	9.78E-12	down	oxidoreductase family protein
19889972	BBA_06960	-1.03	1.01E-10	down	alternative oxidase
19884605	BBA_01593	1.09	2.71E-19	up	quinone oxidoreductase
19885566	BBA_02554	1.10	2.83E-06	up	thioredoxin reductase GliT
19889579	BBA_06567	1.17	3.61E-11	up	Catalase-like domain, heme-dependent
19887112	BBA_04100	1.20	3.35E-14	up	GMC oxidoreductase
19884976	BBA_01964	1.22	1.64E-23	up	ferric-chelate reductase
19886415	BBA_03403	1.46	2.46E-07	up	thioredoxin-like protein
19892610	BBA_09598	1.52	6.80E-11	up	oxidoreductase, short-chain dehydrogenase/reductase family
19885502	BBA_02490	1.58	1.01E-14	up	FAD dependent oxidoreductase
19883459	BBA_00447	1.65	8.96E-20	up	oxidoreductase, short chain dehydrogenase/reductase family
19883458	BBA_00446	2.33	5.86E-34	up	oxidoreductase-like protein
19891701	BBA_08689	2.60	1.15E-13	up	Putative GMC oxidoreductase
19892121	BBA_09109	2.67	6.77E-39	up	Catalase-like domain, heme-dependent
19889198	BBA_06186	2.98	4.07E-95	up	Catalase-like domain, heme-dependent
19885787	BBA_02775	3.20	3.47E-87	up	Amine oxidase
19891770	BBA_08758	4.06	1.25E-68	up	FAD dependent oxidoreductase

19892767	BBA_09755	7.32	8.17E-57	up	epoxide hydrolase
**Involved in cell wall composition and integrity**					
19886828	BBA_03816	-5.18	2.36E-72	down	lectin-like protein
19890150	BBA_07138	-3.98	1.20E-93	down	conidial wall protein
19888941	BBA_05929	-3.19	1.18E-111	down	WSC domain-containing protein
19883537	BBA_00525	-3.03	5.94E-71	down	GPI anchored cell wall protein
19885008	BBA_01996	-2.91	6.67E-69	down	GPI anchored protein, putative
19886969	BBA_03957	-2.05	2.33E-46	down	integral membrane protein
19892140	BBA_09128	-1.71	4.24E-24	down	Concanavalin A-like lectin/glucanase
19886083	BBA_03071	-1.60	2.36E-03	down	hydrophobin-like protein
19891368	BBA_08356	-1.41	4.27E-20	down	integral membrane protein (Pth11)
19891226	BBA_08214	-1.35	3.11E-11	down	cell wall glucanosyltransferase Mwg1
19892480	BBA_09468	-1.01	3.75E-10	down	WSC domain-containing protein
19892737	BBA_09725	1.00	3.84E-08	up	integral membrane protein, putative
19888820	BBA_05808	1.01	1.79E-14	up	cell wall protein
19886027	BBA_03015	1.11	4.37E-20	up	class I hydrophobin
19886427	BBA_03415	1.12	3.07E-07	up	calcofluor white hypersensitive protein
19888684	BBA_05672	1.14	1.34E-15	up	beta-glucosidase, putative
19886133	BBA_03121	1.17	5.51E-16	up	cell surface protein
19888274	BBA_05262	1.24	3.61E-06	up	WSC domain-containing protein
19888028	BBA_05016	1.61	2.67E-03	up	integral membrane protein pth11
19889388	BBA_06376	1.74	6.92E-40	up	glycine-rich cell wall structural protein 1
19885793	BBA_02781	1.75	2.42E-21	up	WSC domain-containing protein
19888768	BBA_05756	1.81	3.78E-36	up	cell wall serine-threonine-rich galactomannoprotein Mp1
19885056	BBA_02044	1.85	1.95E-39	up	integral membrane protein, putative
19886008	BBA_02996	2.31	1.88E-51	up	antigenic cell wall galactomannoprotein, putative
19892186	BBA_09174	3.07	5.45E-62	up	cell surface protein
19885614	BBA_02602	5.51	1.40E-235	up	cell wall protein
19886257	BBA_03245	9.64	4.45E-89	up	cell wall protein
19886505	BBA_03493	11.36	0.00E+00	up	cell wall protein
19886259	BBA_03247	11.81	2.77E-111	up	cell wall protein
19886258	BBA_03246	12.08	0.00E+00	up	cell wall protein
**Involved in carbon/nitrogen metabolisms and energy conversion**					
19891196	BBA_08184	-15.30	6.73E-55	down	glutathione-s-transferase
19888148	BBA_05136	-10.45	0.00E+00	down	carbon catabolite repressor
19890303	BBA_07291	-10.10	0.00E+00	down	peptidase A4 family protein
19891234	BBA_08222	-9.98	1.35E-35	down	nonribosomal peptide synthase, putative
19890517	BBA_07505	-7.48	5.12E-168	down	peptidase A4 family protein
19890652	BBA_07640	-7.11	4.02E-159	down	tyrosinase-like protein
19891758	BBA_08746	-5.54	1.22E-89	down	glutathione-S-transferase theta, GST
19887956	BBA_04944	-4.76	1.54E-135	down	phytoene synthase/lycopene cyclase
19891488	BBA_08476	-4.69	6.31E-206	down	serine carboxypeptidase S28
19883096	BBA_00084	-4.48	9.67E-49	down	Peptidase M64, IgA
19886895	BBA_03883	-4.44	1.08E-173	down	phytase, putative
19889358	BBA_06346	-4.36	1.59E-141	down	oxaloacetate acetylhydrolase
19887401	BBA_04389	-3.89	2.08E-18	down	ATP synthase F1
19883804	BBA_00792	-3.80	2.07E-130	down	beta-1, 3 exoglucanase precursor

19887386	BBA_04374	-3.65	2.05E-29	down	polyketide synthase, putative
19883540	BBA_00528	-3.65	2.24E-140	down	short chain dehydrogenase
19892307	BBA_09295	-3.46	4.51E-62	down	glutathione-dependent formaldehyde-activating enzyme
19890757	BBA_07745	-3.45	5.27E-67	down	Glycoside hydrolase, catalytic core
19883628	BBA_00616	-3.34	4.04E-119	down	glycoside hydrolase family 35
19892439	BBA_09427	-2.96	8.42E-101	down	serine carboxypeptidase
19888563	BBA_05551	-2.90	2.93E-81	down	Beta-lactamase-type transpeptidase
19890755	BBA_07743	-2.86	1.35E-86	down	serine peptidase, putative
19885245	BBA_02233	-2.82	5.57E-12	down	Glycoside hydrolase, catalytic core
19885714	BBA_02702	-2.64	4.49E-48	down	salicylate hydroxylase
19886320	BBA_03308	-2.62	7.13E-10	down	fatty acid hydroxylase superfamily protein
19893117	BBA_10105	-2.39	4.76E-53	down	nonribosomal peptide synthase
19888172	BBA_05160	-2.37	3.35E-67	down	glycolipid anchored surface protein
19891553	BBA_08541	-2.37	7.88E-27	down	short-chain dehydrogenase
19890686	BBA_07674	-2.23	1.16E-47	down	Peptidase S33, tripeptidyl-peptidase
19886777	BBA_03765	-2.22	1.94E-15	down	thiamin biosynthesis protein (Thi-4)
19887957	BBA_04945	-2.22	4.62E-31	down	phytoene dehydrogenase
19884511	BBA_01499	-2.20	3.86E-47	down	nitrate assimilation regulatory protein nirA
19886841	BBA_03829	-2.20	3.59E-50	down	peptidase family M3
19887839	BBA_04827	-2.15	8.62E-34	down	peptide synthetase
19883601	BBA_00589	-2.08	7.41E-35	down	pyruvate dehydrogenase
19892440	BBA_09428	-2.03	5.21E-09	down	peptidase family M3
19890400	BBA_07388	-2.01	4.31E-45	down	Glycoside hydrolase, catalytic core
19892485	BBA_09473	-2.00	2.22E-24	down	dethiobiotin synthetase
19888824	BBA_05812	-1.97	7.33E-40	down	hexose transporter
19884669	BBA_01657	-1.93	2.34E-45	down	prolyl oligopeptidase
19885042	BBA_02030	-1.92	2.77E-41	down	aldehyde dehydrogenase
19885140	BBA_02128	-1.90	2.65E-07	down	Glycoside hydrolase, family 31
19883787	BBA_00775	-1.88	1.20E-41	down	glutamate/Leucine/Phenylalanine/Valine dehydrogenase
19891777	BBA_08765	-1.88	1.39E-04	down	tyrosinase-like protein
19888372	BBA_05360	-1.86	1.76E-36	down	tyrosine phosphatase
19893181	BBA_10169	-1.84	1.70E-21	down	ATPase protein
19885667	BBA_02655	-1.82	8.49E-12	down	carbon-nitrogen hydrolase
19885948	BBA_02936	-1.79	1.73E-16	down	peptidase family protein
19891910	BBA_08898	-1.78	2.81E-30	down	Peptidase cysteine/serine, trypsin
19887540	BBA_04528	-1.77	3.51E-04	down	geranylgeranyl pyrophosphate synthetase
19884822	BBA_01810	-1.73	1.49E-22	down	nonribosomal peptide synthase
19890473	BBA_07461	-1.72	6.77E-13	down	Peptidase S8/S53, subtilisin/kexin/sedolisin
19892194	BBA_09182	-1.69	2.14E-31	down	Glycoside hydrolase, family 36
19893180	BBA_10168	-1.69	1.96E-14	down	geranylgeranyl pyrophosphate synthetase
19892175	BBA_09163	-1.62	1.45E-31	down	carbohydrate-binding module family 21
19889439	BBA_06427	-1.62	6.82E-29	down	glycoside hydrolase family 13
19889094	BBA_06082	-1.60	5.58E-29	down	ATP synthase F0
19888641	BBA_05629	-1.58	1.57E-27	down	carbohydrate esterase family 9
19885734	BBA_02722	-1.55	1.40E-20	down	nitrate assimilation regulatory protein nirA
19889449	BBA_06437	-1.54	8.32E-24	down	Glycoside hydrolase, catalytic core
19891636	BBA_08624	-1.48	3.12E-19	down	ATP synthase F0
19889518	BBA_06506	-1.46	1.05E-19	down	carbohydrate esterase family 3

19888194	BBA_05182	-1.45	1.59E-16	down	ATPase protein
19885597	BBA_02585	-1.44	1.27E-07	down	dihydrodipicolinate synthase
19884666	BBA_01654	-1.44	2.80E-23	down	oxalate decarboxylase family bicupin
19890680	BBA_07668	-1.44	4.25E-10	down	peptidase C14
19888179	BBA_05167	-1.42	2.11E-05	down	peptidase family M48
19884404	BBA_01392	-1.41	3.88E-17	down	glycosyltransferase family 2
19891254	BBA_08242	-1.41	1.78E-07	down	glycoside hydrolase family 43 protein
19885772	BBA_02760	-1.38	3.76E-22	down	Beta-lactamase-type transpeptidase
19883331	BBA_00319	-1.38	1.00E-09	down	Peptidase S8
19883409	BBA_00397	-1.35	6.97E-22	down	hexose transporter
19890601	BBA_07589	-1.33	2.76E-09	down	nonribosomal peptide synthase, putative
19891913	BBA_08901	-1.29	2.08E-14	down	Peptidase S8/S53, subtilisin/kexin/sedolisin
19885666	BBA_02654	-1.29	6.33E-09	down	carbon-nitrogen hydrolase
19885444	BBA_02432	-1.28	3.29E-14	down	triacylglycerol lipase
19888997	BBA_05985	-1.27	2.90E-19	down	nitrate assimilation regulatory protein nirA
19885415	BBA_02403	-1.26	5.89E-20	down	glycosyl hydrolase, putative
19890551	BBA_07539	-1.26	1.61E-15	down	sugar hydrolase
19883088	BBA_00076	-1.25	6.40E-06	down	thiamin biosynthesis protein (Thi-4)
19886064	BBA_03052	-1.24	7.81E-14	down	peptidase family M20/M25/M40
19885452	BBA_02440	-1.24	1.76E-12	down	peptidase M61 domain protein
19883675	BBA_00663	-1.24	2.03E-10	down	beta-lactamase family protein
19888549	BBA_05537	-1.23	8.63E-15	down	polysaccharide synthase Cps1p
19884081	BBA_01069	-1.22	2.38E-15	down	fatty-acid amide hydrolase
19888246	BBA_05234	-1.21	5.90E-13	down	glycosyl hydrolase
19886976	BBA_03964	-1.19	2.70E-13	down	Glycoside hydrolase, catalytic core
19892266	BBA_09254	-1.19	9.68E-13	down	nitrate assimilation regulatory protein nirA
19888224	BBA_05212	-1.18	7.31E-17	down	ATP synthase F0
19889812	BBA_06800	-1.18	7.73E-12	down	beta-galactosidase
19886628	BBA_03616	-1.15	3.88E-09	down	polyketide synthase, putative
19883802	BBA_00790	-1.14	4.82E-14	down	L-serine dehydratase
19892334	BBA_09322	-1.13	1.23E-04	down	chitin synthase
19892090	BBA_09078	-1.11	5.32E-04	down	beta-hexosaminidase
19893185	BBA_10173	-1.10	4.24E-12	down	K-3-type glutaminase
19889391	BBA_06379	-1.10	3.10E-08	down	glycoside hydrolase family 16 protein
19884097	BBA_01085	-1.09	5.29E-07	down	peptidase family M1
19886006	BBA_02994	-1.08	1.79E-11	down	glycosyltransferase family 20
19891170	BBA_08158	-1.08	2.38E-05	down	ATP-dependent protease La domain-containing protein
19883637	BBA_00625	-1.07	9.92E-08	down	Glycoside hydrolase, catalytic core
19890623	BBA_07611	-1.04	5.26E-06	down	non-ribosomal peptide synthetase
19884466	BBA_01454	-1.04	3.22E-12	down	aldehyde dehydrogenase
19890209	BBA_07197	-1.03	3.69E-13	down	Peptidase M19, renal dipeptidase
19884891	BBA_01879	1.01	3.64E-13	up	glycosyltransferase family 17
19891398	BBA_08386	1.01	8.44E-17	up	pyruvate decarboxylase
19889098	BBA_06086	1.01	2.03E-06	up	glycerophosphoryl diester phosphodiesterase family protein
19889166	BBA_06154	1.04	5.49E-10	up	triacylglycerol lipase
19886983	BBA_03971	1.04	8.49E-17	up	Glycoside hydrolase, family 31
19888959	BBA_05947	1.05	6.85E-05	up	short chain dehydrogenase
19887779	BBA_04767	1.07	3.54E-06	up	glycosyl hydrolase family 76
19890422	BBA_07410	1.09	1.19E-13	up	glutathione S-transferase domain-containing protein

19893304	BBA_10292	1.10	2.84E-11	up	cytochrome c oxidase assembly factor
19889673	BBA_06661	1.13	1.64E-04	up	nonribosomal peptide synthase, putative
19888684	BBA_05672	1.14	1.34E-15	up	beta-glucosidase, putative
19887603	BBA_04591	1.14	1.24E-20	up	glycerophosphoryl diester phosphodiesterase
19891436	BBA_08424	1.14	3.27E-12	up	nonribosomal peptide synthase, putative
19888869	BBA_05857	1.15	7.41E-16	up	short chain dehydrogenase
19885809	BBA_02797	1.18	3.62E-16	up	Serine family amino acid catabolism-related protein, putative
19892489	BBA_09477	1.19	3.77E-14	up	hexose transporter
19886765	BBA_03753	1.20	5.71E-22	up	glutamate/Leucine/Phenylalanine/Valine dehydrogenase
19891531	BBA_08519	1.20	3.46E-09	up	lactoylglutathione lyase
19886156	BBA_03144	1.20	4.11E-04	up	short chain dehydrogenase
19887073	BBA_04061	1.22	1.80E-22	up	tyrosinase 2
19890531	BBA_07519	1.23	4.12E-12	up	glucose sorbosone dehydrogenase
19883775	BBA_00763	1.23	1.10E-24	up	glutaryl-CoA dehydrogenase
19891081	BBA_08069	1.25	7.11E-06	up	glycosyl transferase
19886661	BBA_03649	1.30	1.56E-08	up	glucose-repressible protein
19890955	BBA_07943	1.32	1.98E-28	up	glycerophosphoryl diester phosphodiesterase
19891740	BBA_08728	1.34	1.15E-18	up	hexose transporter
19887026	BBA_04014	1.35	9.45E-11	up	inositol phospholipid synthesis protein Scs3p
19889364	BBA_06352	1.35	4.66E-04	up	glycoside hydrolase family 76
19888780	BBA_05768	1.50	3.60E-24	up	cysteine synthase
19892181	BBA_09169	1.50	2.53E-33	up	Hexose transport-related protein, putative
19885707	BBA_02695	1.55	1.48E-14	up	short chain dehydrogenase
19887040	BBA_04028	1.56	1.19E-18	up	nonribosomal peptide synthetase 10
19891439	BBA_08427	1.57	2.77E-33	up	fatty acid hydroxylase superfamily protein
19890295	BBA_07283	1.58	2.64E-25	up	alcohol dehydrogenase GroES-like domain-containing protein
19887700	BBA_04688	1.62	6.35E-29	up	glycosyl hydrolase family 76
19885836	BBA_02824	1.62	5.51E-07	up	short chain dehydrogenase
19892002	BBA_08990	1.64	5.74E-34	up	pyruvate decarboxylase
19889019	BBA_06007	1.67	1.68E-26	up	short-chain dehydrogenase
19886958	BBA_03946	1.68	9.61E-28	up	glycosyl hydrolase family 2
19886094	BBA_03082	1.69	1.90E-17	up	beta (1-3) glucanosyltransferase
19887151	BBA_04139	1.70	2.03E-42	up	glutamate 5-kinase
19893146	BBA_10134	1.70	5.83E-43	up	fatty acid hydroxylase superfamily protein
19889388	BBA_06376	1.74	6.92E-40	up	glycine-rich cell wall structural protein 1
19890768	BBA_07756	1.76	5.33E-32	up	prolyl oligopeptidase
19885591	BBA_02579	1.83	2.83E-46	up	beta-glucuronidase
19892611	BBA_09599	1.84	1.50E-44	up	alcohol dehydrogenase GroES-like domain-containing protein
19890510	BBA_07498	1.92	3.69E-08	up	fatty acid-biding protein
19889495	BBA_06483	2.01	1.12E-23	up	Lactamase B
19889844	BBA_06832	2.06	1.25E-45	up	fatty acid hydroxylase superfamily protein
19891273	BBA_08261	2.23	2.28E-30	up	inositol oxygenase, putative
19889178	BBA_06166	2.51	8.14E-74	up	alcohol dehydrogenase I
19883819	BBA_00807	2.51	8.36E-47	up	glucose repressible protein Grg1
19885593	BBA_02581	2.52	3.69E-59	up	fatty acid hydroxylase superfamily protein
19888024	BBA_05012	2.63	1.84E-17	up	Carbohydrate kinase, thermoresistant glucokinase
19890635	BBA_07623	2.75	1.09E-79	up	trehalase-like protein
19883482	BBA_00470	2.79	4.45E-81	up	ATP-grasp enzyme-like protein

19886962	BBA_03950	2.91	3.93E-22	up	Beta-lactamase-type transpeptidase
19890004	BBA_06992	2.92	7.36E-86	up	short chain dehydrogenase
19890003	BBA_06991	3.00	2.56E-96	up	alcohol dehydrogenase GroES-like domain-containing protein
19891677	BBA_08665	3.11	9.02E-11	up	trypsin-like protease
19886964	BBA_03952	3.29	3.58E-25	up	glutathione-dependent formaldehyde-activating, GFA
19887102	BBA_04090	4.39	1.98E-125	up	short-chain dehydrogenase
19886507	BBA_03495	6.34	8.33E-152	up	fatty acid hydroxylase superfamily protein
**Involved in heat tolerance**					
19885069	BBA_02057	-2.04	3.55E-19	down	heat shock protein 30
19893292	BBA_10280	-1.86	2.56E-13	down	DnaJ domain containing protein
19891640	BBA_08628	-1.56	4.87E-10	down	Hsp70 family chaperone
19892721	BBA_09709	-1.47	1.96E-14	down	Hsp70 family chaperone, putative
19889530	BBA_06518	-1.22	3.17E-09	down	DnaJ domain-containing protein
19886639	BBA_03627	-1.06	1.60E-13	down	Hsp90 associated co-chaperone
19889861	BBA_06849	-1.04	4.88E-09	down	thermotolerance protein
**Involved in cellular transport and homeostasis**					
19891192	BBA_08180	-9.48	0.00E+00	down	MFS multidrug resistance transporter, putative
19887217	BBA_04205	-7.56	2.12E-175	down	major facilitator superfamily transporter
19892091	BBA_09079	-6.19	5.81E-65	down	major facilitator superfamily protein
19883029	BBA_00017	-4.81	1.97E-43	down	ABC transporter
19886458	BBA_03446	-4.70	6.77E-26	down	MFS transporter
19890043	BBA_07031	-4.07	9.95E-41	down	MFS multidrug transporter
19889428	BBA_06416	-3.91	1.42E-38	down	membrane transporter
19883552	BBA_00540	-3.72	1.85E-83	down	MFS transporter, putative
19883017	BBA_00005	-3.55	5.00E-120	down	ABC transporter transmembrane region
19888845	BBA_05833	-3.34	3.43E-50	down	major facilitator superfamily transporter
19892405	BBA_09393	-3.29	3.90E-53	down	sugar transport protein
19892538	BBA_09526	-2.98	1.82E-11	down	MFS transporter
19885463	BBA_02451	-2.80	2.09E-84	down	oligopeptide transporter
19889770	BBA_06758	-2.58	7.59E-63	down	Na/K ATPase alpha 1 subunit, putative
19883863	BBA_00851	-2.57	8.43E-23	down	small oligopeptide transporter, OPT family
19890828	BBA_07816	-2.56	2.87E-73	down	major facilitator superfamily transporter
19891072	BBA_08060	-2.54	7.09E-20	down	MFS monosaccharide transporter, putative
19888461	BBA_05449	-2.48	5.49E-62	down	metabolite transport protein GIT1
19889570	BBA_06558	-2.42	2.49E-15	down	ABC transporter, putative
19892856	BBA_09844	-2.39	1.99E-14	down	vacuolar calcium ion transporter /H(+) exchanger
19891253	BBA_08241	-2.39	1.03E-52	down	general alpha-glucoside permease
19884817	BBA_01805	-2.37	1.11E-23	down	MFS transporter
19887702	BBA_04690	-2.21	3.29E-58	down	Na,H/K antiporter P-type ATPase
19888821	BBA_05809	-2.13	8.33E-26	down	MFS allantoate transporter, putative
19891485	BBA_08473	-2.11	1.14E-16	down	siderophore iron transporter mirA
19886678	BBA_03666	-2.08	4.64E-17	down	ABC bile acid transporter, putative
19886530	BBA_03518	-2.02	1.99E-19	down	major facilitator superfamily transporter
19883780	BBA_00768	-1.81	4.09E-36	down	major facilitator superfamily transporter
19890148	BBA_07136	-1.75	9.20E-06	down	major facilitator superfamily transporter
19890798	BBA_07786	-1.75	2.02E-33	down	calcium-translocating P-type ATPase
19888853	BBA_05841	-1.74	3.09E-34	down	ABC transporter, putative

19884513	BBA_01501	-1.72	4.54E-19	down	major facilitator superfamily transporter
19892135	BBA_09123	-1.69	8.40E-10	down	multidrug resistance protein MDR, putative
19883837	BBA_00825	-1.69	1.07E-35	down	Major Facilitator Superfamily protein
19888637	BBA_05625	-1.69	1.54E-18	down	metal ion transporter metal ion transporter
19885837	BBA_02825	-1.69	7.65E-10	down	proline transporter
19885385	BBA_02373	-1.68	2.49E-08	down	major facilitator superfamily transporter
19889351	BBA_06339	-1.58	9.48E-18	down	major facilitator superfamily transporter
19892348	BBA_09336	-1.56	4.65E-07	down	MFS quinate transporter, putative
19893068	BBA_10056	-1.52	1.02E-25	down	Amino acid/polyamine transporter I
19889873	BBA_06861	-1.51	4.32E-08	down	inner membrane transport protein yfaV
19892688	BBA_09676	-1.46	1.82E-06	down	MFS allantoate transporter
19890673	BBA_07661	-1.46	1.49E-23	down	potassium/sodium efflux P-type ATPase
19883956	BBA_00944	-1.46	1.59E-11	down	nitrite transporter
19884409	BBA_01397	-1.44	8.75E-04	down	ABC multidrug transporter, putative
19883195	BBA_00183	-1.44	2.63E-07	down	major facilitator superfamily transporter
19889168	BBA_06156	-1.36	2.70E-20	down	sulfate transporter
19884348	BBA_01336	-1.33	1.33E-17	down	bicyclomycin resistance protein
19889082	BBA_06070	-1.32	6.59E-05	down	ABC multidrug transporter
19886416	BBA_03404	-1.32	2.73E-06	down	MFS multidrug transporter
19886455	BBA_03443	-1.29	1.58E-05	down	ABC transporter
19884224	BBA_01212	-1.29	1.59E-18	down	sodium/calcium exchanger protein
19889287	BBA_06275	-1.29	4.41E-19	down	general amino acid permease AGP2
19887836	BBA_04824	-1.27	2.17E-11	down	ABC transporter with duplicated ATPase domains
19889432	BBA_06420	-1.25	1.54E-18	down	oligopeptide transporter
19892364	BBA_09352	-1.25	3.05E-15	down	MFS allantoate transporter, putative
19891320	BBA_08308	-1.25	4.98E-18	down	OPT oligopeptide transporter
19888623	BBA_05611	-1.24	1.65E-16	down	copper-transporting ATPase
19884326	BBA_01314	-1.22	3.30E-08	down	peptide transporter MTD1
19884942	BBA_01930	-1.22	3.75E-17	down	OPT peptide transporter Mtd1
19891632	BBA_08620	-1.21	1.53E-13	down	drug resistance protein
19888406	BBA_05394	-1.20	7.38E-14	down	C4-dicarboxylate transporter/malic acid transporter
19886578	BBA_03566	-1.19	9.18E-18	down	transmembrane amino acid transporter
19888748	BBA_05736	-1.19	8.86E-05	down	MFS monosaccharide transporter (Hxt8)
19888398	BBA_05386	-1.16	2.93E-12	down	major facilitator superfamily transporter
19885607	BBA_02595	-1.12	1.52E-07	down	major facilitator superfamily transporter
19885827	BBA_02815	-1.12	1.53E-08	down	ABC transporter
19886191	BBA_03179	-1.11	2.32E-10	down	calcium-transporting ATPase
19891518	BBA_08506	-1.08	1.26E-14	down	multidrug and toxin extrusion protein
19887969	BBA_04957	-1.08	1.38E-09	down	potassium/sodium efflux P-type ATPase
19883860	BBA_00848	-1.06	4.38E-10	down	copper homeostasis protein cutC
19890561	BBA_07549	-1.05	7.77E-07	down	MFS transporter
19885612	BBA_02600	-1.05	3.73E-04	down	MFS multidrug transporter
19889480	BBA_06468	-1.04	7.62E-09	down	ABC transporter
19889572	BBA_06560	-1.02	1.54E-11	down	ABC transporter
19888839	BBA_05827	-1.02	2.13E-11	down	siderophore iron transporter
19891800	BBA_08788	-1.00	6.72E-06	down	MFS sugar transporter, putative
19885917	BBA_02905	1.00	4.18E-04	up	protein transport protein YOS1
19889843	BBA_06831	1.01	1.06E-08	up	major facilitator superfamily transporter
19890760	BBA_07748	1.01	1.00E-10	up	ABC transporter

19890579	BBA_07567	1.02	6.62E-12	up	nonspecific lipid-transfer protein
19889954	BBA_06942	1.02	1.53E-05	up	MFS multidrug transporter, putative
19889435	BBA_06423	1.03	3.91E-14	up	cation transport ATPase
19886596	BBA_03584	1.06	2.37E-09	up	ABC transporter
19887508	BBA_04496	1.07	3.23E-06	up	putative sugar transporter
19887052	BBA_04040	1.08	1.13E-10	up	MFS monocarboxylate transporter
19886472	BBA_03460	1.08	1.25E-19	up	phosphatidylinositol transfer protein CSR1
19884295	BBA_01283	1.09	1.11E-19	up	major facilitator superfamily transporter
19886487	BBA_03475	1.09	2.36E-07	up	bicyclomycin resistance protein
19884705	BBA_01693	1.09	1.03E-19	up	major facilitator superfamily transporter
19888284	BBA_05272	1.16	8.31E-21	up	ABC transporter
19888678	BBA_05666	1.16	3.77E-16	up	MFS transporter
19891483	BBA_08471	1.18	2.51E-08	up	MFS allantoate transporter, putative
19891575	BBA_08563	1.19	1.71E-19	up	ABC transporter, putative
19886013	BBA_03001	1.20	1.85E-07	up	ABC-type Fe3+ transport system
19884572	BBA_01560	1.22	3.79E-11	up	triose-phosphate transporter
19884492	BBA_01480	1.29	6.00E-23	up	major facilitator superfamily transporter
19883563	BBA_00551	1.30	5.72E-23	up	major facilitator superfamily transporter
19888752	BBA_05740	1.30	6.81E-10	up	major facilitator superfamily transporter
19883456	BBA_00444	1.31	2.63E-24	up	nucleoside transporter, putative
19883295	BBA_00283	1.32	1.60E-06	up	membrane transporter
19890727	BBA_07715	1.34	1.73E-12	up	major facilitator superfamily transporter
19890161	BBA_07149	1.41	1.22E-07	up	major facilitator superfamily transporter
19892203	BBA_09191	1.41	8.88E-10	up	MIP transporter
19889774	BBA_06762	1.46	6.94E-25	up	ctr copper transporter
19886122	BBA_03110	1.47	9.35E-17	up	OPT oligopeptide transporter
19885773	BBA_02761	1.48	1.18E-27	up	Major facilitator superfamily, general substrate transporter
19886303	BBA_03291	1.49	1.44E-34	up	MFS monocarboxylate transporter
19885303	BBA_02291	1.52	1.21E-06	up	V-type ATPase
19886063	BBA_03051	1.58	8.85E-16	up	polyamine transporter 3
19892731	BBA_09719	1.62	1.91E-26	up	MFS toxin efflux pump (AflT)
19890397	BBA_07385	1.64	3.19E-37	up	MFS transporter
19886245	BBA_03233	1.66	5.10E-27	up	GDP-mannose transporter
19892758	BBA_09746	1.67	8.24E-08	up	major facilitator superfamily transporter
19884115	BBA_01103	1.68	2.74E-15	up	ABC transporter
19886885	BBA_03873	1.69	2.13E-30	up	calcium/proton exchanger
19890293	BBA_07281	1.80	2.54E-16	up	urea active transporter
19887045	BBA_04033	1.88	9.91E-25	up	General substrate transporter
19891189	BBA_08177	2.01	1.20E-19	up	cation transporter
19890132	BBA_07120	2.06	1.06E-43	up	major facilitator superfamily transporter
19885211	BBA_02199	2.19	1.84E-32	up	neutral amino acid transporter
19886590	BBA_03578	2.21	5.16E-55	up	siderophore iron transporter mirB
19891440	BBA_08428	2.26	8.94E-36	up	efflux pump antibiotic resistance protein
19893278	BBA_10266	2.66	1.15E-30	up	sugar transporter
19888332	BBA_05320	3.12	9.95E-81	up	plasma membrane zinc ion transporter, putative
19886224	BBA_03212	3.17	9.30E-119	up	phthalate transporter
19888779	BBA_05767	3.24	2.62E-126	up	tetracycline efflux protein (otrb)
19885944	BBA_02932	3.25	1.76E-58	up	General substrate transporter
19892313	BBA_09301	3.28	7.23E-12	up	amino acid transporter

19889285	BBA_06273	3.32	4.12E-73	up	OPT oligopeptide transporter
19883811	BBA_00799	3.92	7.77E-170	up	phosphate transporter
19886260	BBA_03248	12.05	0.00E+00	up	major facilitator superfamily transporter
**Involved in transcriptional regulation**					
19890648	BBA_07636	-6.98	4.40E-66	down	C6 transcription factor
19885603	BBA_02591	-3.39	1.84E-93	down	C6 zinc finger domain protein
19888302	BBA_05290	-2.93	7.01E-45	down	fungal specific transcription factor
19887098	BBA_04086	-2.91	5.36E-52	down	Zn2C6-type transcription factor
19892310	BBA_09298	-2.81	9.66E-47	down	Zn(II)2Cys6 transcription factor
19890066	BBA_07054	-2.75	2.32E-57	down	C6 zinc finger domain-containing protein
19883871	BBA_00859	-2.41	2.63E-65	down	Putative Zn(II)2Cys6 transcription factor
19883474	BBA_00462	-2.37	1.81E-51	down	APSES transcription factor Xbp1
19887174	BBA_04162	-2.33	7.95E-46	down	ZIP zinc/iron transporter
19891193	BBA_08181	-2.27	2.23E-26	down	fungal specific transcription factor
19883856	BBA_00844	-2.17	1.08E-44	down	fungal specific transcription factor
19889132	BBA_06120	-2.10	5.23E-43	down	C2H2 type zinc finger domain protein
19884113	BBA_01101	-1.87	5.09E-10	down	C6 zinc finger domain-containing protein
19892936	BBA_09924	-1.86	2.27E-10	down	zinc finger protein
19888235	BBA_05223	-1.77	1.32E-17	down	fungal specific transcription factor
19891535	BBA_08523	-1.77	4.21E-13	down	fungal specific transcription factor domain protein
19885579	BBA_02567	-1.74	7.05E-37	down	nitrogen assimilation transcription factor nirA
19883251	BBA_00239	-1.60	1.00E-13	down	transcription factor Domain protein family
19890903	BBA_07891	-1.56	1.15E-20	down	bZIP transcription factor
19887334	BBA_04322	-1.53	3.06E-19	down	transcription factor
19892563	BBA_09551	-1.52	6.33E-12	down	C6 transcription factor, putative
19890721	BBA_07709	-1.46	2.53E-13	down	transcription factor RfeG
19887342	BBA_04330	-1.45	3.06E-21	down	BZIP-type transcription factor
19888832	BBA_05820	-1.41	1.16E-14	down	C6 transcription factor, putative
19884242	BBA_01230	-1.39	2.90E-19	down	ZIP Zinc transporter
19890143	BBA_07131	-1.37	8.00E-06	down	C3HC4 type (RING finger) zinc finger containing protein
19887970	BBA_04958	-1.37	7.70E-18	down	RadR putative transcriptional regulator
19887772	BBA_04760	-1.36	3.80E-08	down	Putative Zn(II)2Cys6 transcription factor
19890413	BBA_07401	-1.29	7.79E-10	down	fungal specific transcription factor
19890083	BBA_07071	-1.29	1.24E-14	down	C6 zinc finger domain-containing protein
19892424	BBA_09412	-1.29	1.47E-18	down	trascription factor
19888974	BBA_05962	-1.28	2.30E-16	down	fungal specific transcription factor, putative
19889069	BBA_06057	-1.27	7.27E-20	down	Fungal transcriptional regulatory protein
19889294	BBA_06282	-1.26	1.03E-17	down	fungal specific transcription factor
19883724	BBA_00712	-1.25	1.88E-11	down	putative C6-zinc finger TF, regulator of conidiation
19886189	BBA_03177	-1.22	5.84E-05	down	fungal specific transcription factor
19884514	BBA_01502	-1.21	6.18E-12	down	fungal specific transcription factor
19883740	BBA_00728	-1.20	5.97E-18	down	zinc-binding dehydrogenase
19885362	BBA_02350	-1.20	8.32E-09	down	zinc finger protein 76 (expressed in testis)
19889076	BBA_06064	-1.20	1.29E-08	down	GATA zinc finger protein
19888310	BBA_05298	-1.14	2.94E-07	down	DNA repair and transcription factor Ada, putative
19884453	BBA_01441	-1.14	1.16E-07	down	zinc finger protein SFP1
19886026	BBA_03014	-1.13	1.39E-09	down	bZIP transcription factor
19888635	BBA_05623	-1.13	1.02E-05	down	zinc finger protein

19883117	BBA_00105	-1.13	4.91E-03	down	Putative Zn(II)2Cys6 transcription factor
19886476	BBA_03464	-1.09	1.39E-12	down	C6 finger domain protein
19890939	BBA_07927	-1.06	1.84E-12	down	C2 domain-containing protein
19887315	BBA_04303	-1.02	4.63E-07	down	LYAR-type C2HC zinc finger protein
19890312	BBA_07300	-1.02	1.96E-04	down	C6 transcription factor
19891664	BBA_08652	-1.01	2.63E-08	down	transcription factor tfiiic complex subunit sfc6
19892345	BBA_09333	-1.01	6.17E-04	down	C2H2 finger domain protein, putative
19890105	BBA_07093	-1.01	1.55E-04	down	RING finger protein
19892415	BBA_09403	1.05	4.78E-04	up	bZIP transcription factor
19892709	BBA_09697	1.05	4.65E-08	up	C2H2 transcription factor
19885519	BBA_02507	1.06	7.12E-20	up	fungal specific transcription factor
19892061	BBA_09049	1.21	1.41E-17	up	RING finger domain protein
19884690	BBA_01678	1.27	3.85E-14	up	ZIP Zinc transporter family protein
19892016	BBA_09004	1.41	8.99E-21	up	Putative C2H2 finger domain transcription factor
19893237	BBA_10225	1.42	5.80E-19	up	C6 finger domain protein, putative
19890430	BBA_07418	1.47	4.26E-04	up	fungal specific transcription factor
19884713	BBA_01701	1.50	3.65E-21	up	C6 finger domain protein
19889362	BBA_06350	1.68	3.96E-17	up	C6 transcription factor
19887307	BBA_04295	1.85	2.53E-37	up	C2 domain-containing protein
19887101	BBA_04089	2.42	6.78E-49	up	zinc-binding dehydrogenase
19892448	BBA_09436	2.72	8.67E-47	up	zinc finger-like protein
19885023	BBA_02011	2.85	1.96E-13	up	zinc finger protein Nv-ZicA
19892449	BBA_09437	3.06	5.65E-30	up	C2H2 finger domain protein, putative
19892640	BBA_09628	3.23	5.65E-21	up	C6 zinc finger domain-containing protein
19891451	BBA_08439	3.23	2.56E-96	up	zinc-binding dehydrogenase
19892966	BBA_09954	3.42	3.45E-122	up	C2H2 finger domain protein, putative
19888690	BBA_05678	3.54	4.54E-59	up	nitrogen assimilation transcription factor nirA
19891979	BBA_08967	3.73	1.28E-92	up	fungal zinc cluster transcription factor
19885778	BBA_02766	3.74	6.90E-49	up	BTB domain transcription factor
19890354	BBA_07342	3.79	1.65E-19	up	C6 transcription factor
19892447	BBA_09435	5.36	1.22E-190	up	C6 zinc finger domain-containing protein
**Involved in postranslational modifications and chromatin remodeling**					
19892473	BBA_09461	-3.29	1.20E-121	down	methyltransferase-like protein
19889340	BBA_06328	-2.99	1.24E-85	down	methyltransferase-like protein
19892474	BBA_09462	-2.72	4.54E-85	down	methyltransferase-like protein
19888404	BBA_05392	-2.65	2.26E-65	down	O-methyltransferase-like protein
19891513	BBA_08501	-2.25	3.21E-58	down	putative SAM-dependent methyltransferase
19887670	BBA_04658	-2.16	6.78E-56	down	methyltransferase-like protein
19886129	BBA_03117	-2.08	4.71E-21	down	Acyl-CoA N-acyltransferase
19885406	BBA_02394	-2.01	2.10E-18	down	methyltransferase-like protein
19892472	BBA_09460	-1.91	2.11E-19	down	SAM dependent carboxyl methyltransferase
19886450	BBA_03438	-1.82	4.43E-35	down	Acyl-CoA N-acyltransferase
19883946	BBA_00934	-1.79	2.78E-23	down	sterigmatocystin 8-O-methyltransferase
19885171	BBA_02159	-1.77	1.59E-28	down	homoserine O-acetyltransferase
19884161	BBA_01149	-1.61	2.74E-21	down	alpha-1,3-mannosyltransferase CMT1
19892887	BBA_09875	-1.49	1.05E-02	down	O-methyltransferase, family 3
19891312	BBA_08300	-1.44	7.25E-13	down	arylamine N-acetyltransferase 1
19889339	BBA_06327	-1.39	4.03E-03	down	methyltransferase-like protein

19885193	BBA_02181	-1.38	1.31E-24	down	ornithine carbamoyltransferase
19883054	BBA_00042	-1.37	3.51E-23	down	homoserine acetyltransferase family protein
19885007	BBA_01995	-1.36	3.10E-22	down	methyltransferase-like protein
19893069	BBA_10057	-1.28	4.95E-16	down	Acyl-CoA N-acyltransferase
19889315	BBA_06303	-1.25	3.85E-17	down	methyltransferase domain-containing protein
19891551	BBA_08539	-1.20	8.69E-04	down	putative SAM-dependent methyltransferase
19887674	BBA_04662	-1.19	1.23E-16	down	SAM-dependent methyltransferase
19891262	BBA_08250	-1.19	1.91E-15	down	methyltransferase-like protein
19887972	BBA_04960	-1.15	7.57E-04	down	Methyltransferase type 11
19889573	BBA_06561	-1.14	8.42E-13	down	histone-lysine N-methyltransferase
19884431	BBA_01419	-1.11	1.23E-14	down	adenine phosphoribosyltransferase
19888070	BBA_05058	-1.10	1.69E-08	down	acyltransferase-like protein
19891505	BBA_08493	-1.06	6.83E-07	down	Acyl-CoA N-acyltransferase
19890647	BBA_07635	-1.04	2.08E-08	down	SAM-dependent methyltransferase
19888922	BBA_05910	-1.02	6.68E-10	down	isoprenylcysteine carboxyl methyltransferase
19888459	BBA_05447	1.08	8.96E-07	up	S-adenosylmethionine-dependent methyltransferase, putative
19891590	BBA_08578	1.08	8.64E-09	up	Acyl-CoA N-acyltransferase
19890856	BBA_07844	1.09	5.72E-15	up	O-methyltransferase, family 3
19891937	BBA_08925	1.12	1.50E-09	up	O-methyltransferase, family 3
19886592	BBA_03580	1.13	4.27E-21	up	FkbM family methyltransferase
19885374	BBA_02362	1.13	2.07E-18	up	C-5 cytosine-specific DNA methylase
19892756	BBA_09744	1.31	4.14E-09	up	O-methyltransferase-like protein
19883450	BBA_00438	1.35	2.03E-09	up	GNAT family acetyltransferase, putative
19885925	BBA_02913	1.47	1.05E-22	up	methyltransferase domain-containing protein
19891579	BBA_08567	1.54	5.78E-22	up	methyltransferase domain-containing protein
19888129	BBA_05117	1.55	1.15E-06	up	SET domain-containing protein
19892510	BBA_09498	1.56	2.64E-07	up	FkbM family methyltransferase
19883105	BBA_00093	1.66	9.43E-09	up	methyltransferase domain-containing protein
19886594	BBA_03582	1.68	7.52E-40	up	O-methyltransferase family protein
19892450	BBA_09438	2.21	3.87E-08	up	Methyltransferase type 11
19888160	BBA_05148	2.24	2.67E-26	up	Acyl-CoA N-acyltransferase
19893156	BBA_10144	3.02	2.18E-41	up	Methyltransferase type 11
19893267	BBA_10255	4.74	8.96E-116	up	Methyltransferase type 11
**Involved in DNA splicing, repair, reverse transcription and translation**					
19890679	BBA_07667	-5.07	2.79E-49	down	reverse transcriptase
19893368	BBA_10356	-1.68	1.47E-15	down	reverse transcriptase
19884676	BBA_01664	-1.60	1.71E-28	down	DNA photolyase
19883598	BBA_00586	-1.53	8.19E-26	down	DNA polymerase POL4, putative
19892217	BBA_09205	-1.50	1.06E-11	down	reverse transcriptase
19890784	BBA_07772	-1.43	2.92E-08	down	reverse transcriptase
19884028	BBA_01016	-1.35	1.02E-21	down	Ribonuclease/ribotoxin
19885436	BBA_02424	-1.31	8.08E-14	down	DASH family cryptochrome
19885818	BBA_02806	-1.30	8.81E-21	down	splicing factor Spf30
19888310	BBA_05298	-1.14	2.94E-07	down	DNA repair and transcription factor Ada, putative
19889890	BBA_06878	-1.12	7.69E-14	down	DNA repair protein (Rad57), putative
19884262	BBA_01250	-1.08	4.86E-05	down	DNA polymerase II large subunit-like protein
19893365	BBA_10353	-1.06	2.29E-06	down	reverse transcriptase
19893308	BBA_10296	1.06	7.55E-10	up	reverse transcriptase

19887384	BBA_04372	1.08	1.01E-16	up	DNA repair protein (Rad57), putative
19884046	BBA_01034	1.14	1.73E-16	up	DNA photolyase
19883204	BBA_00192	1.21	1.64E-07	up	Ribonuclease H1
19892435	BBA_09423	1.69	4.05E-08	up	translation factor (SUA5)
19887116	BBA_04104	3.79	1.48E-98	up	DNA-binding protein
**Involved in cellular signaling**					
19888236	BBA_05224	-7.64	2.89E-60	down	protein phosphatase 2C
19893363	BBA_10351	-3.18	1.85E-28	down	serine/threonine protein kinase Japonica Group
19891255	BBA_08243	-2.78	1.15E-75	down	calcineurin-like phosphoesterase
19886774	BBA_03762	-2.67	4.88E-20	down	protein kinase subdomain-containing protein
19886234	BBA_03222	-2.60	2.57E-30	down	serine/threonine protein kinase
19892247	BBA_09235	-2.31	5.39E-54	down	putative dual specificity protein kinase pom1
19892930	BBA_09918	-1.88	2.40E-23	down	protein kinase domain-containing protein
19893252	BBA_10240	-1.70	2.03E-28	down	histidine acid phosphatase
19890942	BBA_07930	-1.68	1.22E-18	down	protein kinase subdomain-containing protein
19893088	BBA_10076	-1.67	1.55E-11	down	CAMK family protein kinase
19885329	BBA_02317	-1.60	8.98E-20	down	serine/threonine-protein kinase nak1
19887082	BBA_04070	-1.50	4.38E-12	down	calcium/calmodulin dependent protein kinase C, putative
19893364	BBA_10352	-1.49	2.02E-13	down	protein kinase domain-containing protein
19885628	BBA_02616	-1.49	1.34E-07	down	receptor-interacting serine/threonine-protein kinase
19888600	BBA_05588	-1.42	2.85E-18	down	MAP kinase kinase skh1/pek1
19884107	BBA_01095	-1.27	3.02E-16	down	MAP kinase kinase 1
19889919	BBA_06907	-1.26	6.14E-19	down	histidine acid phosphatase
19885572	BBA_02560	-1.24	1.31E-04	down	dual specificity phosphatase Yvh1, putative
19888798	BBA_05786	-1.19	8.59E-07	down	protein kinase domain-containing protein
19885080	BBA_02068	-1.12	1.51E-13	down	calcineurin-like phosphoesterase
19886503	BBA_03491	-1.08	3.35E-13	down	tousled-like kinase
19889532	BBA_06520	-1.04	1.14E-06	down	calcineurin-like phosphoesterase
19885115	BBA_02103	1.06	4.03E-07	up	spindle assembly checkpoint kinase
19890578	BBA_07566	1.10	7.86E-16	up	histidinol phosphate phosphatase HisJ family protein
19890596	BBA_07584	1.21	2.21E-13	up	MAP kinase kinase skh1/pek1
19885004	BBA_01992	1.27	4.72E-26	up	calcineurin-like phosphoesterase
19889610	BBA_06598	1.32	8.22E-08	up	histidine acid phosphatase
19892972	BBA_09960	1.52	7.39E-18	up	Rho-associated protein kinase, putative
19890791	BBA_07779	1.52	2.27E-13	up	serine-threonine protein kinase
19887733	BBA_04721	2.03	4.91E-19	up	serine protein kinase
19892055	BBA_09043	2.72	9.75E-38	up	mitogen-activated protein kinase sty1
